# Rapid detection of neurons in widefield calcium imaging datasets after training with synthetic data

**DOI:** 10.1038/s41592-023-01838-7

**Published:** 2023-04-01

**Authors:** Yuanlong Zhang, Guoxun Zhang, Xiaofei Han, Jiamin Wu, Ziwei Li, Xinyang Li, Guihua Xiao, Hao Xie, Lu Fang, Qionghai Dai

**Affiliations:** 1 Department of Automation, Tsinghua University, Beijing, China; 2 Institute for Brain and Cognitive Sciences, Tsinghua University, Beijing, China; 3 IDG/McGovern Institute for Brain Research, Tsinghua University, Beijing, China; 4 Beijing Laboratory of Brain and Cognitive Intelligence, Beijing Municipal Education Commission, Beijing, China; 5 Shanghai Artificial Intelligence Laboratory, Shanghai, China; 6 School of Information Science and Technology, Fudan University, Shanghai, China; 7 Department of Electronic Engineering, Tsinghua University, Beijing, China

**Keywords:** Fluorescence imaging, Machine learning, Image processing, Neuroscience, Mouse

## Abstract

Widefield microscopy can provide optical access to multi-millimeter fields of view and thousands of neurons in mammalian brains at video rate. However, tissue scattering and background contamination results in signal deterioration, making the extraction of neuronal activity challenging, laborious and time consuming. Here we present our deep-learning-based widefield neuron finder (DeepWonder), which is trained by simulated functional recordings and effectively works on experimental data to achieve high-fidelity neuronal extraction. Equipped with systematic background contribution priors, DeepWonder conducts neuronal inference with an order-of-magnitude-faster speed and improved accuracy compared with alternative approaches. DeepWonder removes background contaminations and is computationally efficient. Specifically, DeepWonder accomplishes 50-fold signal-to-background ratio enhancement when processing terabytes-scale cortex-wide functional recordings, with over 14,000 neurons extracted in 17 h.

## Abstract (web summary)

DeepWonder removes background signals from widefield calcium recordings and enables accurate and efficient neuronal segmentation with high throughput.

## Main

Optical microscopy technologies^1–3^ and genetically encoded calcium indicators^4^ help researchers study brain function in various behavioral tasks^5,6^. During image acquisition within the scattering brain, researchers have to contend with fundamental limitations imposed by serial and parallel acquisition schemes^7^. Serial acquisition approaches such as two-photon laser-scanning microscopy (TPLSM) provide optical sectioning and robustness to scattering^8^, but have low temporal resolution across millimeter-scale field-of-view (FOV)^9,10^. Although multiplexing methods substantially increase the TPLSM frame rate across large cortical areas, the necessary high power dosage in the animal brain^11^ could result in heat-induced damage^12^. With regard to the spatial scale, TPLSM has been pushed to a FOV of ~5 mm in diameter^9,10,13–16^, but this typically requires temporal subsampling of calcium dynamics for a cortex-wide region-of-interest (ROI). On the other hand, parallel schemes such as widefield microscopy^7,17,18^, combined with the growing gamut of array sensors, provide neuroscientists with a practical tool capable of video-rate acquisition over multi-millimeter-scaled ROIs at single-cell resolution^19^. With the help of an optimized optical setup and computational tools, widefield microscopy has enabled recordings of large neuron populations across tens of mammalian brain regions in a 10 × 8 mm^2^ FOV at a pixel size of 0.8 µm (ref. ^20^), with potential to record millions of neurons simultaneously^17^. However, scattering-induced crosstalk and background contaminations challenge widefield functional microscopy. Since the widefield microscope illuminates and detects the whole volume of the sample, neurons away from the focal plane contribute ambiguous background signals^21^. Light scattering in opaque tissue further deterioriates fluorescent signals originating from the focal plane and distorts information about neuron locations and activities. To reduce these effects, researchers typically have to sacrifice imaging speed^22^ or even sample health^23^.

Computational approaches can separate neuronal signals from background contamination in widefield microscopy. The constrained nonnegative matrix factorization (CNMF-E) approach models the strong background signals with prior knowledge of the spatiotemporal signal properties^24^. However, refining the background model for widefield imaging concomitantly requires sophisticated parameter tuning and is computationally demanding, precluding its use for cortex-scale neuronal processing^25^. Online processing with a lightweight version of the algorithm partially alleviates the speed problem, but at the expense of performance^26^. Other methods^25,27,28^ without explicit modeling of the fluctuating background could achieve higher processing speed, but commonly face the risks of residual background contaminations^26^. Thus, analyzing widefield calcium recordings in scattering mammalian brains by established computational methods is far from optimal in terms of jointly achieving both high speed and considerable performance.

Artificial neural networks have achieved breakthroughs in neuronal image processing tasks such as image enhancement^29^, neuronal segmentation^30,31^ and spike inference^32^. With proper training, deep-learning-based neuronal activity inference in TPLSM data can achieve an order-of-magnitude-faster speed with no compromise in performance^30^. However, little attention has been paid to leveraging deep learning for background removal in widefield neuronal recordings, given the lack of paired widefield and background-free data for training. Methods that convert background models into trainable convolutional filters alleviate the requirement of paired data, but need per-sample retraining and compromise in performance compared with alternative neuron extraction methods^26^.

**Fig. 1:**
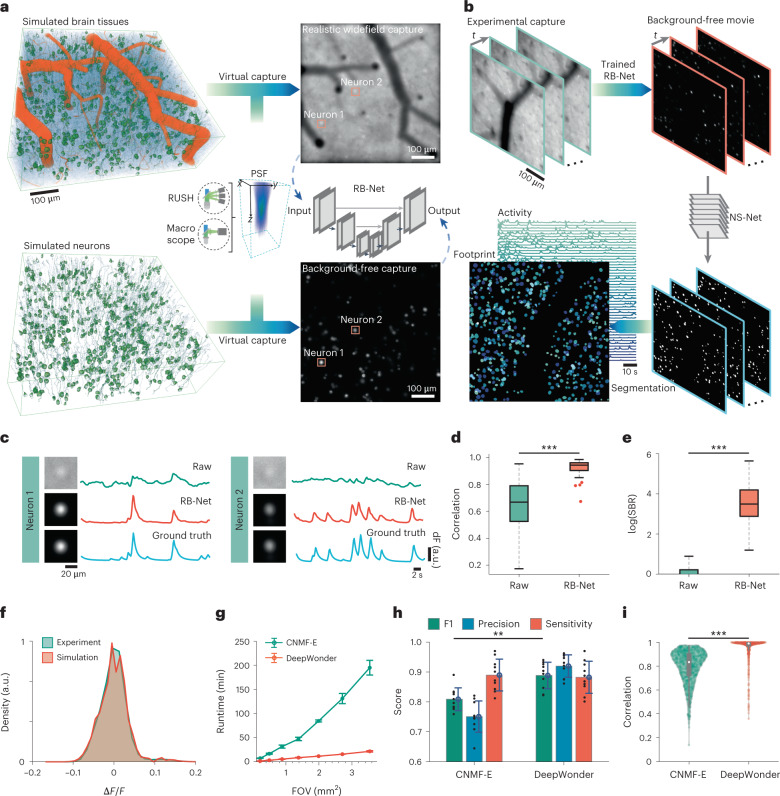
Principle of deep-learning-enhanced widefield neuron finder (DeepWonder). **a**, Training stage of RB-Net in DeepWonder. On the basis of microscope and imaging parameters, the widefield simulator generates synthetic recordings with high similarities to experimental data as inputs to RB-Net. Simultaneously, recordings with similar neuron distributions but without background contaminations as labels are generated. Both inputs and labels are used to train RB-Net such that it can restore background-free neuronal images from background-contaminated images. **b**, Illustration of the application of DeepWonder on experimental recordings. The trained RB-Net in DeepWonder removes the background of experimental recordings. A NS-Net then segments neurons and extracts neuronal signals from the movies without background. **c**, Restoration of calcium transients (corresponding to neurons labeled in **a** from the raw data (green) by DeepWonder (red). Traces without background contamination serve as ground truth (blue) for comparison. Fluorescence change (dF) scaled for clarity. a.u., arbitrary units. **d**, Neuronal signal correlations with ground truth in raw movie (green) and in DeepWonder-processed movie (red). ***P < 1 × 10^−50^, two-sided Wilcoxon signed-rank test, *n* = 901 neurons from six simulated recordings. Central black mark: median. Bottom and top edges: 25th and 75th percentiles. Whiskers extend to extreme points excluding outliers (1.5 times above or below the interquartile range). **e**, SBR ratio of raw movie (green) and DeepWonder-processed movie (red). ***P < 1 × 10^−50^, two-sided Wilcoxon signed-rank test, *n* = 901 neurons from six simulated recordings. Box plot elements as in **d**. **f**, Δ*F*/*F* distributions of recordings simulated with NAOMi1p (red) and of experimental recordings (green). **g**, Runtime comparisons of CNMF-E and DeepWonder across different FOVs. The plot shows mean ± s.d. runtime, averaged over *n* = 5 recordings from two mice. In FOV of 3.54 mm^2^, the runtime reduction by DeepWonder is 9.10 ± 0.68 (mean ± s.d. across *n* = 5 recordings). **h**, F1, precision and sensitivity scores of segmentation by CNMF-E are 0.81 ± 0.03, 0.75 ± 0.05 and 0.89 ± 0.04, respectively. F1, precision and sensitivity scores of segmentation by DeepWonder are 0.90 ± 0.03, 0.92 ± 0.03 and 0.88 ± 0.04, respectively. Statistical scores are shown in mean ± s.d. across *n* = 10 simulated recordings. For F1 scores, **P = 0.002, two-sided Wilcoxon signed-rank test. Height of bars: mean. Error bars: s.d. Black dots: *n* = 10 simulated recordings. **i**, Correlation with ground truth of DeepWonder (red, 0.96 ± 0.09, mean ± s.d. across *n* = 714 neurons from five simulated recordings) and CNMF-E (green, 0.79 ± 0.14, mean ± s.d. across *n* = 5 recordings). ***P < 1 × 10^−50^, two-sided Wilcoxon signed-rank test. White circle: median. Thick gray vertical line: interquartile range. Thin vertical lines: upper and lower proximal values. Transparent disks: data points. Transparent violin-shaped areas: kernel density estimate of data distribution. Scale bars, 100 µm and 10 s (**a**,**b**) and 20 µm and 2 s in (**c**). Source data

**Extended Data Fig. 1:**
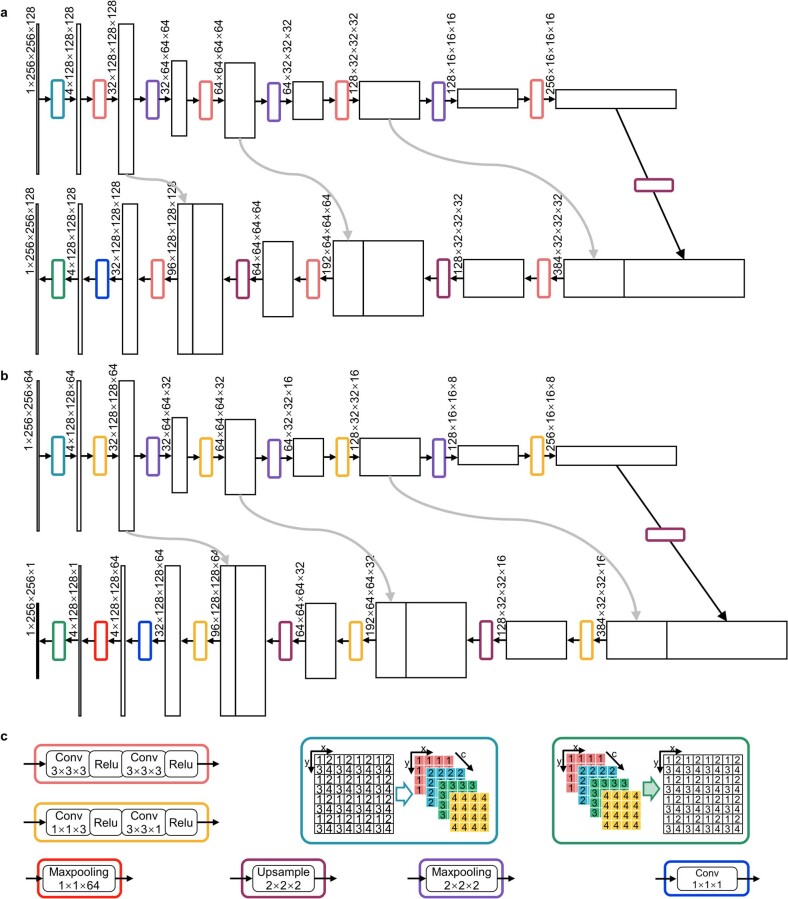
Network architectures of DeepWonder. **a**. The basic structure of removing background network (RB-Net), which consists of a 3D encoding path (top) and a 3D decoding path (bottom). The RB-Net has 4 million parameters. The detailed structures of each colored box in the network diagram are explained in **c**. **b**. The basic structure of neuron segmentation network (NS-Net), which is a 3D Unet but with different channels compared to **a**. In NS-Net, a couple of lightweight convolutional operators supersede the 3×3×3 convolution in RB-Net. A maxpooling layer at the end of NS-Net compresses the image sequence into a mask for segmentation. The NS-Net has 0.9 million parameters. The detailed structures of each colored box in the network diagram are explained in **c**. **c**. Detailed structures of each network part distinguished by different colors. Specially, we introduce “spatial to channel” operator (blue-green)^1^ and “channel to spatial” operator (green) at the beginning and end of RB-Net (**a**) and NS-Net (**b**), respectively, for making full utilization of the computing potential of these two networks. All feature maps are 4D tensors and the order of dimensions marked in the figure is (channel, x, y, t).

**Extended Data Fig. 2:**
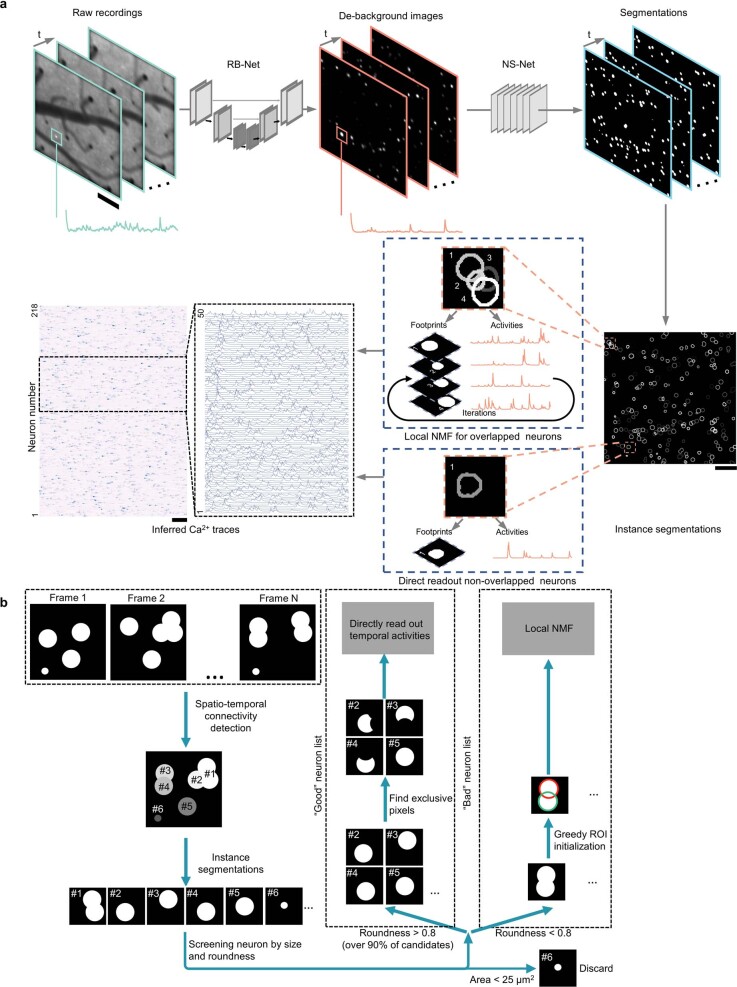
DeepWonder processing pipeline in new data. **a**. Processing pipeline of DeepWonder. The captured raw movie is firstly sent to RB-Net to be converted into a background-free movie, and NS-Net further segments neurons. The segmented neurons are then formed instances, and overlapped neurons are further demixed through non-negative matrix factorization. Final temporal activities of recorded neurons are output through reading the non-overlapped pixels of each neuron. **b**. Processing details of the DeepWonder segmentation module. Output frames from NS-Net are firstly spatiotemporally separated into disconnected neuron candidates, where those spatially overlapped but temporally distinguished candidates are separated (for example #1 and #2). Then neuron candidates are classified by area and roundness. Candidates with an area smaller than 25 µm^2^ will be discarded (for example #6). Candidates with a larger area will be kept but further divided into two groups: the “good” neuron group within which each neuron has roundness ≥ 0.8 (for example #2, #3, #4, and #5), and the “bad” neuron group is with smaller roundness (for example #1). The candidates in the “good” neuron list will be sent out for directly reading out temporal activities based on exclusive pixels (bottom dashed box in the second row of **a**). For each candidate in the “bad” neuron group, greedy ROI initialization is performed^2^ and then the candidate will be sent for local NMF to be further demixed (top dashed box in the second row of **a**). Scale bar: 100 µm and 10 seconds.

In this Article, we developed a deep-learning-based widefield neuron finder (DeepWonder), an efficient widefield neuronal extraction technique with an order-of-magnitude-faster speed and improved performance compared with alternative approaches. By leveraging a hyperrealistic simulation of brain tissue^33^ to generate optical system-specific paired synthetic recordings with and without background, we circumvented the need for contamination-free, experimentally acquired ground truth labels. We then developed an artificial neural network to separate neuronal signals from the scattered background (Fig. 1a, Extended Data Fig. 1 and Supplementary Video 1), as the first stage of DeepWonder. In the second stage, we then applied a lightweight convolutional neural network to quickly segment the cleaned data into neurons to retrieve spatial footprints and temporal signals (Fig. 1b and Extended Data Fig. 2). Using both simulated and experimental data, we demonstrate a nearly tenfold processing speed acceleration and performance improvement with DeepWonder compared with the CNMF-E algorithm. We further validated the accuracy of DeepWonder on a hybrid system with simultaneous widefield and TPLSM recordings of diverse cortical areas across multiple animals in vivo. We deployed DeepWonder on multiple widefield calcium recording systems, including the terabyte-scale real-time, ultra-large-scale, high-resolution (RUSH) system^20^ covering over 14,000 neurons, a large-FOV macroscope^19^, and a widefield hippocampal imaging system^34^. DeepWonder is available as a Python package.

## Results

### Removing background contamination through synthetic data-driven deep learning

**Extended Data Fig. 3:**
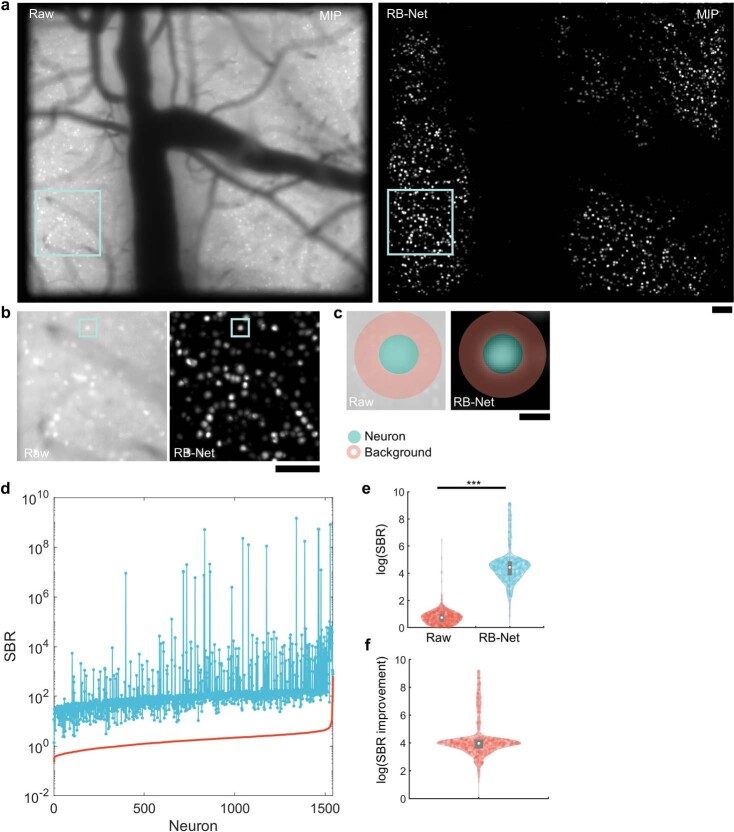
RB-Net in DeepWonder largely increases signal-to-background ratio (SBR). **a**. Comparison of raw widefield recordings (left, with the RUSH system) and DeepWonder enhanced recordings (right). The images shown here were calculated through time-channel-wise maximum intensity projection (MIP). The mean background value for widefield and DeepWonder was 0.61 and 0.01, respectively (normalized by the maximum value). **b**. Zoom-in panel in **a**. **c**. Zoom-in panel in **b**, with schematics of SBR calculation. The SBR is calculated by the ratio of maximum activity across the green area (that is soma mask) over the maximum activity across the pink area (that is background mask). Note each background mask does not overlap with other soma masks (Methods). **d**. SBR in logarithmic scale of all neurons in **a** (n = 1543 neurons from a single recording). Blue dots are for RB-Net, and red dots are for raw captures. **e**. Violin plot of SBR of raw image (red) and RB-Net enhanced image (blue) in logarithmic scale. ***P < 1×10^-50^, two-sided Wilcoxon signed-rank test, n = 1543 neurons from a single recording. White circle: Median. Thick grey vertical line: Interquartile range. Thin vertical lines: Upper and lower proximal values. Transparent disks: Data points. Transparent violin-shaped areas: Kernel density estimate of data distribution. **f**. Violin plot of SBR improvement from raw image to DeepWonder. n = 1543 neurons from a single recording. Violin plot elements as in **e**. Scale bar: 100 µm in a and b, 10 µm in c.

Background contamination, which is mixed with crosstalk among neurons, neuropil and background fluorescence from out-of-focus depths, limits the achievable neuron detection sensitivity and signal extraction quality in widefield microscopy. In DeepWonder, we removed these confounds by establishing an artificial neural network that converts background-contaminated recordings into background-free ones (Fig. 1a). We synthesized hyperrealistic widefield calcium imaging data by modeling vessels, neurons and background dendrites and axons with a specific widefield microscope model^33^, yielding synthetic recordings with hyperrealistic pixel distribution, Δ*F*/*F* distribution and spatial frequency distribution (Supplementary Figs. 1 and 2). As a counterpart, background-free recordings were synthesized by modeling only fluorescent neurons and nonfluorescent vessels in the tissue along with the same microscope model. Paired synthetic recordings were thus generated and fed to our removing background network (RB-Net; Extended Data Fig. 1a and Supplementary Fig. 3), to learn the mapping between background-contaminated experimental data and background-free synthetic data. The trained RB-Net in DeepWonder outputs high-contrast images and realistic neuronal activity without contamination (Fig. 1c and Supplementary Fig. 4). Compared with raw data, DeepWonder significantly enhanced correlation scores with the ground truth signals (Fig. 1d, *n* = 901 neurons, ***P < 1 × 10^−50^, two-sided Wilcoxon signed-rank test) and signal-to-background ratios (SBRs) in test datasets that have never been seen by the network (Fig. 1e, *n* = 901 neurons, ***P < 1 × 10^−50^, two-sided Wilcoxon signed-rank test). Compared with other state-of-the-art background removal methods^25,26^, RB-Net in DeepWonder achieved superior performance in terms of SBRs (Supplementary Fig. 5f), correlation scores (Supplementary Fig. 5g) and neuron finding scores on the same datasets (Supplementary Fig. 5h and Supplementary Note 1), while spending almost eightfold shorter time in removing background (Supplementary Fig. 5i). Owing to the high similarity between the synthetic data and the real recordings (Fig. 1f), the RB-Net entrained by synthetic data in DeepWonder effectively removes backgrounds in experimental data (Fig. 1b, Extended Data Fig. 3 and Supplementary Video 1). With RB-Net we obtained an SBR improvement of more than 50-fold in experimental recordings compared with raw data across 1,543 neurons (Extended Data Fig. 3).

After separating neuronal signals from background contamination, we then used a neuron segmentation network (NS-Net) that efficiently segmented neurons from background-decontaminated data (Fig. 1b). The NS-Net started with a lightweight convolutional neural network that segmented neurons from RB-Net output at a high speed (Extended Data Fig. 1b). Neurons were further semantically segmented on the basis of their spatio-temporal connectivity into mostly exclusive segments. The temporal activities of the individual neurons were directly read out since there was no inter-neuron crosstalk (Extended Data Fig. 2a). Neurons that were tiled and overlapped were further demixed by a local nonnegative matrix factorization (NMF)^35^ algorithm to eliminate activities crosstalk (Extended Data Fig. 2b). NS-Net reliably demixed neurons that were as close as 0.3 of the neuron diameter, yielding a temporal similarity over 0.9 and a spatial similarity over 0.85 (Supplementary Fig. 6). Our NS-Net outperformed state-of-the-art neuron segmentation techniques such as CaImAn batch^36^, STNeuroNet^31^ and SUNS^30^ with the highest sensitivity and F1 score in the background-decontaminated datasets (Supplementary Figs. 7e and 8b,d). The processing speed of NS-Net is eight times faster than CaImAn batch, five times faster than STNeuroNet and comparable to SUNS (Supplementary Figs. 7f and 8e).

**Extended Data Fig. 4:**
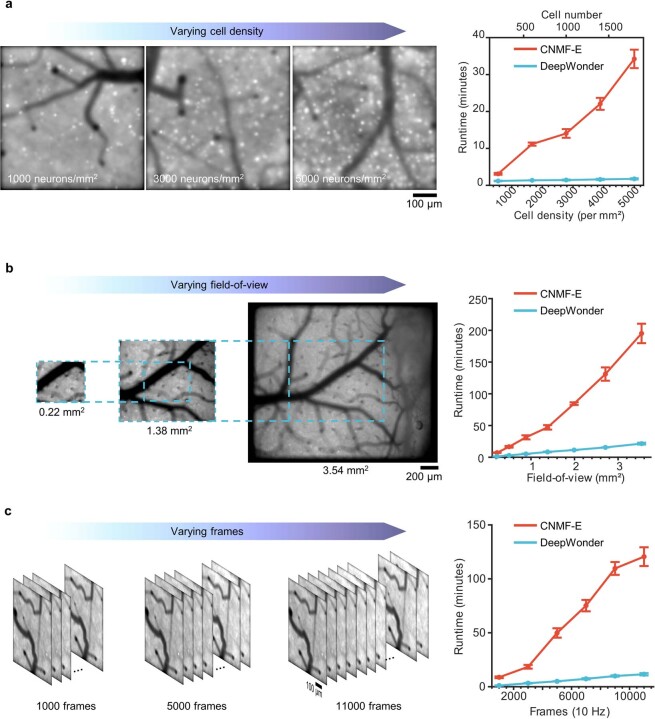
Runtime comparisons of DeepWonder and CNMF-E. **a**. Runtime comparison of DeepWonder and CNMF-E, with increased cell densities from 1000 neurons/mm^2^ to 5000 neurons/mm^2^ in NAOMi1p datasets. Left, examples of maximum intensity projection (MIP) images of simulated recordings in 1000, 3000, and 5000 neurons/mm^2^. Right, runtime comparison between CNMF-E (red) and DeepWonder (blue) in an area of 600 ×600 µm^2^ at fixed 1000 frames with varied neuron density. **b**. Runtime comparison of DeepWonder and CNMF-E, with increased FOV size from 0.22 mm^2^ to 3.54 mm^2^ in RUSH datasets. Left, examples of MIP images of RUSH recordings across FOVs of 0.22, 1.38, and 3.54 mm^2^. Right, runtime comparison between CNMF-E (red) and DeepWonder (blue) in varied FOVs at fixed 2000 frames. **c**. Runtime comparison of DeepWonder and CNMF-E, with increased recording session length in RUSH datasets. Left, examples of RUSH recordings in a FOV of 600 ×600 µm^2^ with 1000, 5000, and 110000 frames. Right, runtime comparison between CNMF-E (red) and DeepWonder (blue) at varied frames in a fixed FOV. All plots show mean ± SD runtime, averaged over n = 5 different recordings. **a** were simulated recordings. **b** and **c** were experimental recordings taken from 2 mice. Scale bar: 100 µm in **a** and **c**, 200 µm in **b**.

By combining the optimized RB-Net and NS-Net into one framework, our DeepWonder achieves a processing speed improvement of nearly tenfold (Fig. 1g and Extended Data Fig. 4) compared with the CNMF-E technique (Supplementary Note 1). DeepWonder additionally improves segmentation and activity inference accuracy, as illustrated by 11.1% improvement in F1 scores (Fig. 1h) and 21.5% improvement in temporal correlation scores (Fig. 1i). The RB-Net in DeepWonder circumvents the time-consuming background modeling process in CNMF-E and achieves background elimination through a single-shot workflow, where the processing speed is only affected by the scale of datasets. The processing speed compares even more favorably when cell density and cell number are higher, typically reaching nearly 20-fold improvement when the neuron density reaches 5,000 cells mm^−2^ (Extended Data Fig. 4a). When processing calcium recordings of over 10,000 frames at 10 Hz, CNMF-E takes over 2 h on average, while DeepWonder takes only 11 min (Extended Data Fig. 4c). DeepWonder is also robust to noise and reaches F1 scores of 0.60 and temporal correlation scores of 0.77 in a condition with a low post-objective excitation power of 0.3 mW mm^−2^, which is 9-fold and 1.6-fold higher than CNMF-E, respectively (Supplementary Fig. 9). In moderately low excitation power situations (0.7 mW mm^−2^), DeepWonder still outperforms CNMF-E in accuracy with an F1 score of 0.82 relative to 0.66 with CNMF-E.

### Validation of DeepWonder through simultaneously acquired functional ground truth

**Fig. 2:**
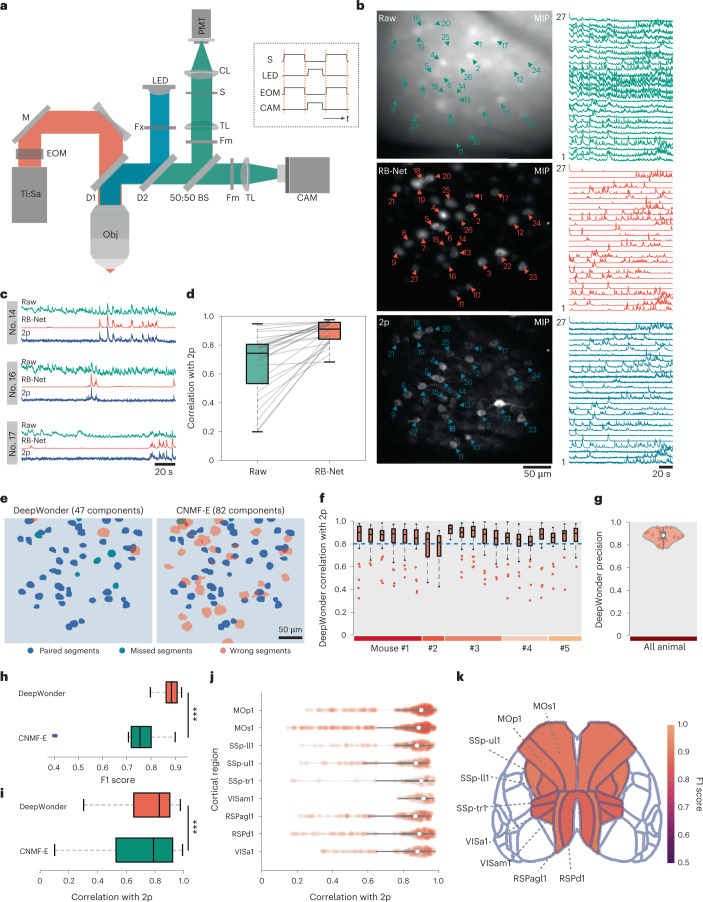
DeepWonder achieves accurate neuron segmentation and activity inference validated by two-photon (2p) microscopy. **a**, The hybrid 1p–2p microscope setup. LED, light-emitting diode light source; Ti:Sa, titanium:sapphire laser; M, mirror; DM, dichroic mirror; BS, beam splitter; Fm, emission filter; Fx, excitation filter; CL, collection lens; TL, tube lens; S, triggerable shutter; CAM, sCMOS camera; Obj, objective. Right box: control signals of the shutter, LED, EOM and camera exposure. **b**, Maximum intensity projection (MIP) of widefield (top), RB-Net in DeepWonder processed widefield movie (middle) and two-photon movie (bottom). Triangles mark neurons, and the corresponding temporal activities are plotted on the right side. The mean background value for widefield, DeepWonder and 2p movies are 0.64, 0.001 and 0.12, respectively (normalized by maximum value). **c**, Zoom-in plots of temporal activities of neurons 14, 16 and 17 in the widefield raw movie (green), RB-Net processed movie (red) and 2p movie (blue.) **d**, Temporal correlations of 27 representative neurons, from raw data imaged with with 2p (0.68 ± 0.22, mean ± s.d.) and after processing with RB-Net (0.89 ± 0.08, mean ± s.d.). ***P = 3.55 × 10^–6^, two-sided Wilcoxon signed-rank test. Central black mark: median. Bottom and top edges: 25th and 75th percentiles. Whiskers extend to extreme points excluding outliers (1.5 times above or below the interquartile range). **e**, DeepWonder (left) and CNMF-E segmentation results (right). Blue masks represent correct segments, green masks represent missed segments and pink masks represent false segments. The precision, sensitivity and F1 score of DeepWonder are 0.94, 0.88, and 0.91, while for CNMF-E are 0.56, 0.96, and 0.73. **f**, Correlations of DeepWonder-extracted neuron activities with activities imaged with 2p across five animals and 20 recordings. Box plot elements as in **d**. **g**, Precision scores of DeepWonder segmented neurons with 2p dataset as the reference across five animals and 20 recordings reach 0.88 ± 0.05 (mean ± s.d.). White circle: median. Thick gray vertical line: interquartile range. Thin vertical lines: upper and lower proximal values. Transparent disks: data points. Transparent violin-shaped areas: Kernel density estimate of data distribution. **h**, F1 scores of DeepWonder (red, 0.88 ± 0.03, mean ± s.d.) and CNMF-E (blue, 0.73 ± 0.13, mean ± s.d.) across five animals and 20 recordings. ***P = 1.38 × 10^–6^, two-sided Wilcoxon signed-rank test. Box plot elements as in **d**. **i**, Temporal correlation of activities obtained with DeepWonder (red, 0.84 ± 0.15, median ± median absolute deviation) or CNMF-E (0.79 ± 0.21, median ± median absolute deviation) with activities derived from 2p across five animals and 20 recordings (*n* = 1,570 neurons). ***P = 9.79 × 10^–23^, two-sided Wilcoxon signed-rank test. Box plot elements as in **d**. **j**, Distribution of temporal correlation between DeepWonder-extracted traces and corresponding 2p ground truth in different brain regions from different animals. The correlation scores for primary motor area layer 1 (MOp1), secondary motor area layer 1 (MOs1), primary somatosensory area lower limb layer 1 (SSp-ll1), primary somatosensory area upper limb layer 1 (SSp-ul1), primary somatosensory area trunk layer 1 (SSp-tr1), anteromedial visual area layer 1 (VISam1), retrosplenial area lateral agranular part layer 1 (RSPagl1), retrosplenial area dorsal part layer 1 (RSPd1) and anterior area layer 1 (VISa1) are 0.85 ± 0.15, 0.81 ± 0.19, 0.84 ± 0.14, 0.80 ± 0.17, 0.82 ± 0.22, 0.90 ± 0.05, 0.80 ± 0.18, 0.80 ± 0.21 and 0.82 ± 0.16, respectively (mean ± s.d.). *n* = 40 sites are recorded from four animals. Violin plot elements as in **g**. **k**, Spatial distribution of neuron detection accurate scores (F-score) achieved by DeepWonder overlaid with Allen CCF atlas^42^. *n* = 40 sites are recorded from four animals. Scale bars, 50 µm and 20 s (**b**), 20 s (**c**) and 50 µm (**e**). Source data

**Extended Data Fig. 5:**
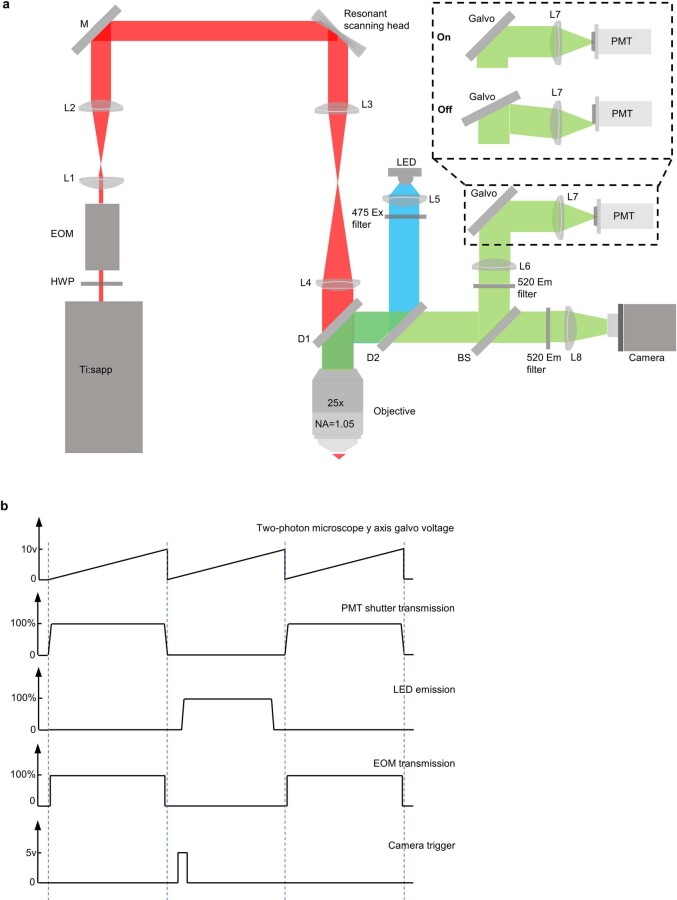
Simultaneous two-photon and widefield neuronal recording system. **a**. Hybrid one-photon and two-photon microscope setup. Ti:sapp: titanium-sapphire laser with tunable wavelength; HWP: half-wave plate; EOM: Electro-Optic Modulator; M: mirror; L1, L2, L3, L4, L5, L6, L7, L8: lens; D1: long-pass dichroic mirror to separate fluorescence signals (centered at 520 nm, green path) and widefield excitation signals (centered at 470 nm, blue path) from two-photon excitation light (centered at 920 nm, red path); D2: long-pass dichroic mirror to separate fluorescence signals (centered at 520 nm, green path) from widefield excitation signals (centered at 470 nm); BS: 50:50 (reflectance: transmission) non-polarizing plate beam splitter; PMT: photomultiplier tubes. The black dashed box in the right shows a galvo mirror which acts as an optical shutter and protects PMT from overexposure when widefield fluorescence is excited. **b**. Control signals and optical transmissions for two-photon microscope y-galvo, the galvo mirror in PMT path (as an optical shutter), the LED emission, EOM, and camera trigger.

To evaluate the inference accuracy of DeepWonder trained with simulated datasets, we next verify its performance with a standard two-photon microscope as the reference. We built a hybrid microscopic device capable of both two-photon and widefield detection modalities. We sequentially switched the co-axis aligned two-photon and one-photon lightpath by timing control of a gated electrical optical modulator (EOM), light-emitting diode (LED) excitation and photon-sensitive photomultiplier tube (PMT) shutter in 30 Hz (Fig. 2a). The shutter was used to protect the sensitive PMT when strong widefield fluorescence was excited (Extended Data Fig. 5). We reduced the two-photon excitation numerical aperture (NA) to 0.27 such that the same neuron population could be detected by both the widefield and two-photon modalities (Supplementary Fig. 10). After image registration, we achieved 15 Hz widefield neuronal recordings and paired 15 Hz two-photon recordings served as functional ground truth (Supplementary Note 2). We found the RB-Net in DeepWonder effectively mapped background-overwhelmed widefield data into sharp ones similar to the two-photon recordings in both spatial profiles (Fig. 2b) and temporal activities (Fig. 2b,c). The correlation scores of DeepWonder output with two-photon signals reached 0.89 ± 0.08 (mean ± standard deviation (s.d.)), significantly outperforming the raw signals (*n* = 27 neurons, ***P = 3.55 × 10^–6^, two-sided Wilcoxon signed-rank test; Fig. 2d and Supplementary Figs. 11 and 12). With DeepWonder, we detected 47 neurons, with 44 of them matching active neurons from two-photon data, leading to an F1 score of 0.91 compared with 0.73 by CNMF-E (Fig. 2e). By analyzing 20 datasets from five mice, DeepWonder achieved over 0.8 median correlation scores in each of the datasets (Fig. 2f), and 0.88 ± 0.05 (mean ± s.d.) precision scores (Fig. 2g) across all datasets, indicating that DeepWonder provides accurate neuronal segmentation and activity inference in mouse recordings. The high temporal fidelity of DeepWonder was further verified by a hybrid system that acquired high-NA two-photon ground truth (correlation scores of 0.83 ± 0.02, mean ± s.d., *n* = 1,545 neurons; two-photon excitation NA 0.6; Supplementary Figs. 13 and 14). Further compared with CNMF-E, DeepWonder achieves both higher accuracy (F1 score 0.88 ± 0.03 of DeepWonder compared with F1 score 0.73 ± 0.13 of CNMF-E, mean ± s.d., *n* = 20; Fig. 2h) and higher signal correlations with two-photon ground truth (Fig. 2i).

**Extended Data Fig. 6:**
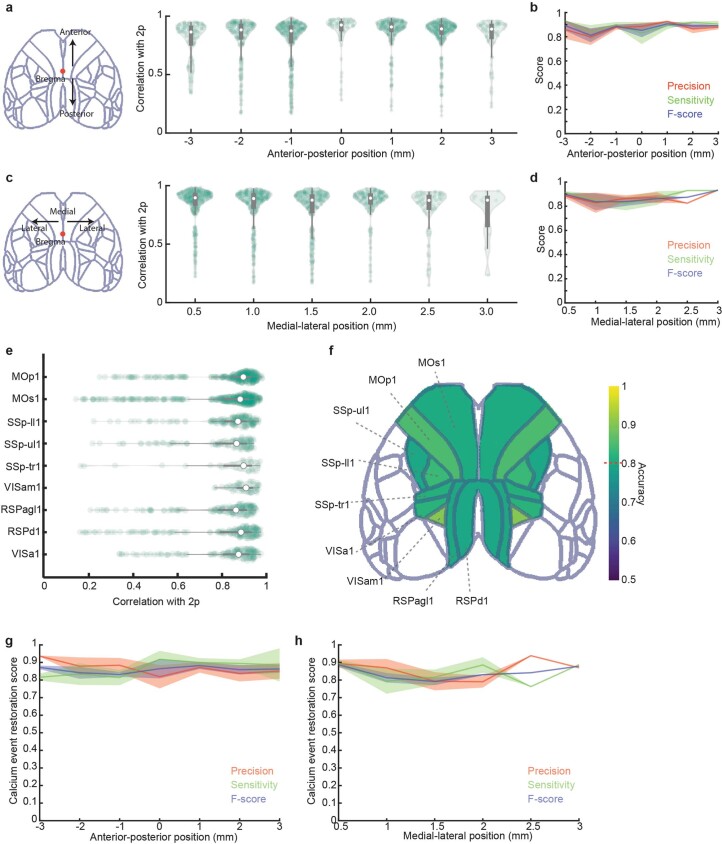
Validation of DeepWonder across brain-wide positions. **a**. Distributions of temporal correlation between DeepWonder-extracted traces and corresponding 2p ground truth traces, as a function of anterior-posterior (AP) positions. White circle: Median. Thick grey vertical line: Interquartile range. Thin vertical lines: Upper and lower proximal values. Transparent disks: Data points. Transparent violin-shaped areas: Kernel density estimate of data distribution. The bregma point (red point) is the zero point, where positions on the anterior side are positive and positions on the posterior side are negative. Data in **a**–**g** is based on a total of 40 recordings from 5 mice. **b**. Neuron detection scores (precision, sensitivity, and F-score) achieved by DeepWonder on experimental functional verification datasets as a function of AP positions. Shaded areas: mean ± SD. **c**. Distributions of temporal correlation between DeepWonder-extracted traces and corresponding 2p ground truth traces, as a function of medial-lateral (ML) positions. Violin plot elements as in **a**. The bregma point (red point) is the zero point, where positions on lateral sides are positive. **d**. Neuron detection scores (precision, sensitivity, and F-score) achieved by DeepWonder on experimental functional verification datasets as a function of ML positions. Shaded areas: mean ± SD. **e**. Distribution of temporal correlation between DeepWonder-extracted traces and corresponding 2p ground truth traces in different brain regions. Violin plot elements as in **a**. **f**. Spatial distribution of neuron detection accurate scores (F-score) achieved by DeepWonder on experimental functional verification datasets overlaid with Allen CCF atlas. For all covered brain regions, the accurate scores are higher than 0.8. **g**. Calcium event detection scores (precision, sensitivity, and F1 score) achieved by DeepWonder on experimental functional verification datasets as a function of anterior-posterior positions. Shaded areas: mean ± SD. To evaluate the detection scores, we firstly deconvolved the calcium traces of DeepWonder-processed widefield recordings and CaImAn-processed 2p recordings using OASIS^6^, then matched the DeepWonder spikes and 2p spikes in a window of 300 ms (Methods). **h**. The same as **g** but as a function of medial-lateral positions.

**Extended Data Fig. 7:**
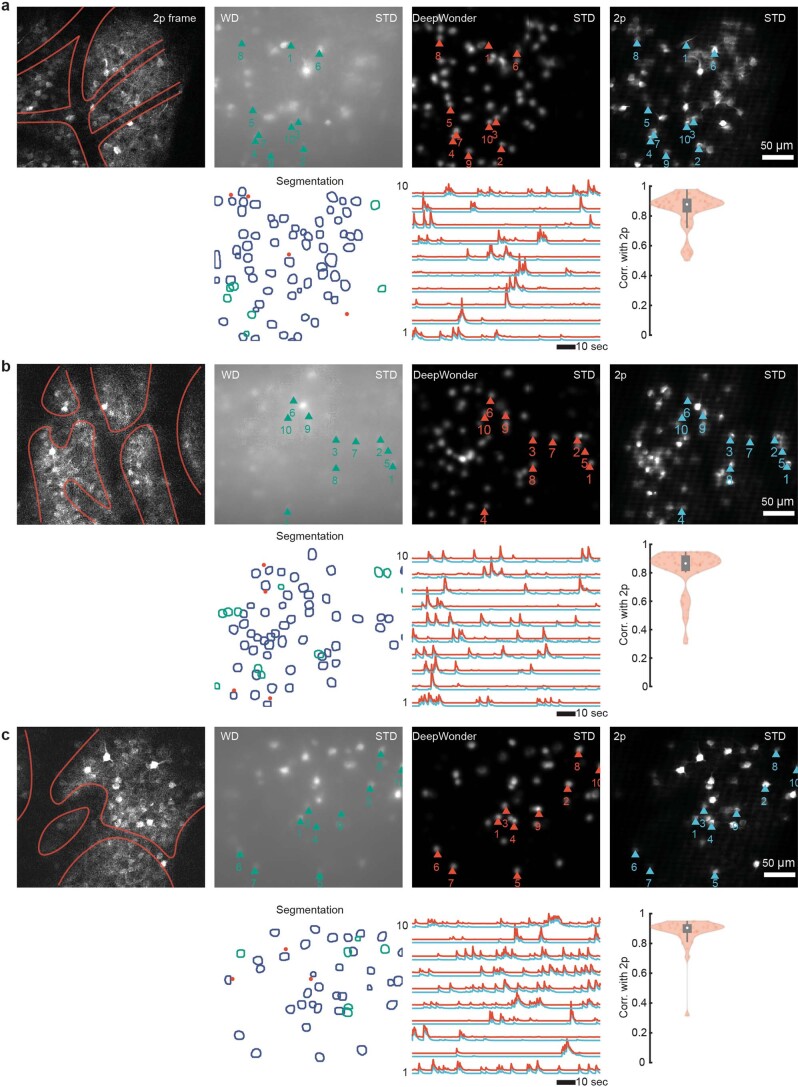
Validation of DeepWonder near complex vessel structures. **a**. Left to right in the first row: an exemplary frame from 2p recordings where blood vessels are outlined in red, standard deviation (STD) in temporal dimension of raw widefield movie (WD), DeepWonder processed movie, and 2p movie. Randomly selected 10 neurons are labeled by arrows and numbers in each panel, where green for raw movie, red for DeepWonder movie, and blue for 2p movie. Left to right in the second row: neuron segmentation by DeepWonder compared to 2p ground truth, neuronal activity traces corresponding to arrows in STD panels as used for performance quantifications, and distributions of temporal correlations between DeepWonder-extracted traces and corresponding traces in 2p ground truth (0.84 ± 0.11, mean ± SD, n = 64 neurons in a single recording). For the segmentation panel, blue circles indicate neurons that are found in both DeepWonder and 2p movies. Green circles indicate neurons that are found only in DeepWonder movie. Red dots are missed neurons found in 2p movie but not in DeepWonder movie. For trace comparison panel, red traces for DeepWonder movie and blue traces for 2p movie, and DeepWonder traces are offset vertically for clarity. For plots of temporal correlation distributions, white circle: Median; Vertical thick grey bar: interquartile range; Thin vertical lines: Upper and lower proximal values; Transparent disks: Data points; Transparent violin-shaped areas: Kernel density estimate of data distribution. **b** and **c** are the same with **a** but from different recordings. n = 59 neurons for **b** and n = 35 neurons for **c**. Scale bar: 50 µm and 10 seconds.

**Extended Data Fig. 8:**
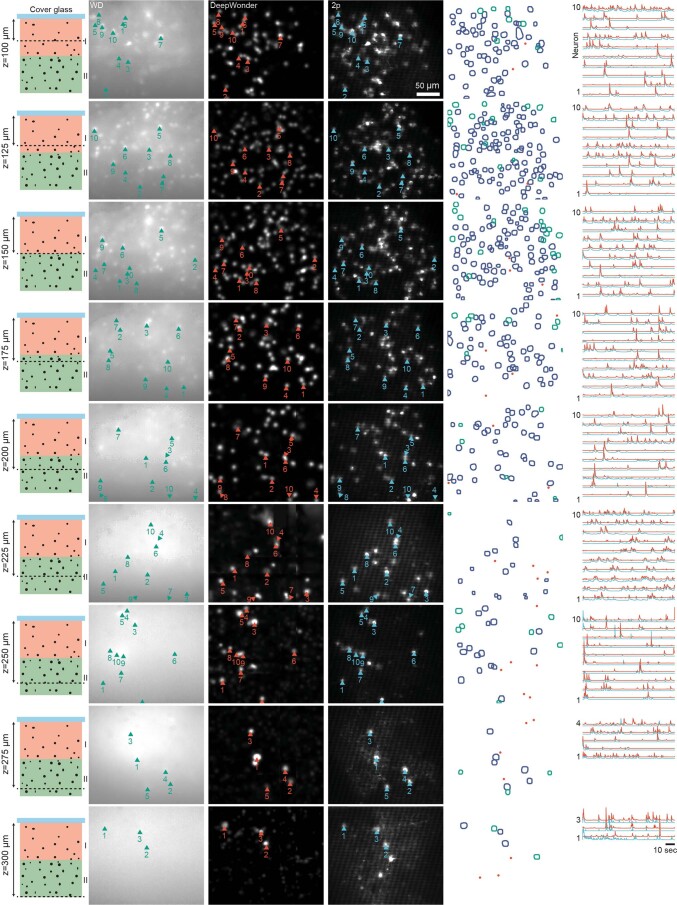
Validation of DeepWonder on different cortical depths. From top to bottom: 8 different cortical depths (100 µm to 300 µm) were accessed by the hybrid widefield-2p detection system and processed by DeepWonder. The first column labels the location of the imaging focal plane (dashed black line) with the imaging depth labeled on the left side. The Roman numerals I, II indicate layer I, II of the dorsal cortex, respectively. The second to the fourth columns show the standard deviation (STD) image across temporal frames of the raw widefield movie, DeepWonder processed movie, and 2p movie, respectively. Randomly selected neurons are labeled by arrows and numbers in each panel, where green for raw movie, red for DeepWonder movie, and blue for 2p movie. The fifth column shows neuron segmentation by DeepWonder compared to 2p ground truth. Blue circles indicate neurons that are found in both DeepWonder and 2p movies. Green circles indicate neurons that are only found in DeepWonder movie. Red dots are missed neurons found in 2p movie but not in DeepWonder movie. The sixth column shows neuronal activity traces corresponding to arrows in STD panels as used for performance quantifications. Red traces for DeepWonder movie and blue traces for 2p movie, and DeepWonder traces are offset vertically for clarity. Scale bar: 50 µm and 10 seconds.

**Extended Data Fig. 9:**
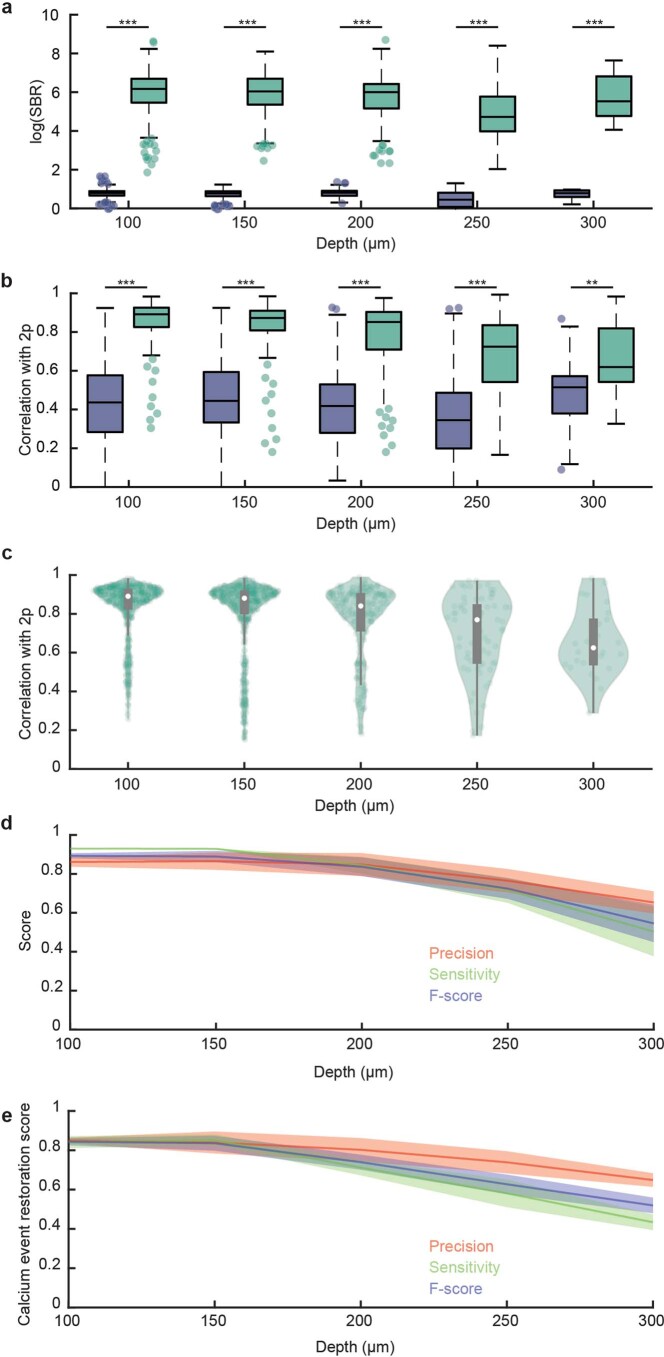
Quantification of DeepWonder across 100-300 µm cortical depth. **a**. Logarithm of signal-to-background ratio (SBR) of neurons in raw movie (blue) and DeepWonder movie (green) from 100 µm to 300 µm imaging depth under dura. ***P < 1×10^-50^, ***P < 1×10^-50^, ***P < 1×10^-50^, ***P = 3.86×10^-24^, ***P = 6.55×10^-10^ for depth 100, 150, 200, 250, and 300 µm, respectively, two-sided Wilcoxon signed-rank test. Data in **a**–**e** is based on a total of 40 recordings from 4 mice. Central black mark: Median. Bottom and top edges: 25th and 75th percentiles. Whiskers extend to extreme points excluding outliers (1.5 times above or below the interquartile range). **b**. Boxplot of correlation scores of neurons in raw movie (blue) and DeepWonder movie (green) with 2p ground truth, from 100 µm to 300 µm imaging depth under dura. ***P < 1×10^-50^, ***P < 1×10^-50^, ***P < 1×10^-50^, ***P = 3.98×10^-11^, ***P = 0.0011 for depth 100, 150, 200, 250, and 300 µm, respectively, two-sided Wilcoxon signed-rank test. Box plot elements as in **a**. **c**. Distributions of temporal correlation between DeepWonder-extracted traces and corresponding 2p traces as a function of depth. Mean ± SD of correlation scores are 0.83 ± 0.15, 0.80 ± 0.18, 0.77 ± 0.20, 0.68 ± 0.21, 0.67 ± 0.18 from 100 to 300 µm imaging depth at a step of 50 µm, respectively. White circle: Median. Thick grey vertical line: Interquartile range. Thin vertical lines: Upper and lower proximal values. Transparent disks: Data points. Transparent violin-shaped areas: Kernel density estimate of data distribution. **d**. Neuron detection scores (precision, sensitivity, and F-score) achieved by DeepWonder on experimental functional verification datasets as a function of depths. Mean ± SD of precision scores are 0.86 ± 0.07, 0.87 ± 0.14, 0.85 ± 0.19, 0.76 ± 0.17, 0.65 ± 0.10 from 100 to 300 µm imaging depth at a step of 50 µm. Mean ± SD of sensitivity scores are 0.93 ± 0.02, 0.92 ± 0.05, 0.84 ± 0.14, 0.72 ± 0.18, 0.50 ± 0.22 from 100 to 300 µm imaging depth at a step of 50 µm. Mean ± SD of F scores are 0.89 ± 0.04, 0.89 ± 0.09, 0.84 ± 0.15, 0.72 ± 0.15, 0.55 ± 0.17 from 100 to 300 µm imaging depth at a step of 50 µm. **e**. Calcium event detection scores (precision, sensitivity, and F-score; Methods) achieved by DeepWonder on experimental functional verification datasets as a function of depths. Mean ± SD of precision scores are 0.85 ± 0.04, 0.84 ± 0.18, 0.80 ± 0.19, 0.74 ± 0.16, 0.65 ± 0.06 from 100 to 300 μm imaging depth at a step of 50 μm. Mean ± SD of sensitivity scores are 0.84 ± 0.09, 0.85 ± 0.09, 0.71 ± 0.12, 0.58 ± 0.20, 0.43 ± 0.07 from 100 to 300 μm imaging depth at a step of 50 μm. Mean ± SD of F scores are 0.84 ± 0.06, 0.84 ± 0.12, 0.74 ± 0.12, 0.63 ± 0.14, 0.52 ± 0.07 from 100 to 300 μm imaging depth at a step of 50 μm. Shaded areas: mean ± SD.

**Extended Data Fig. 10:**
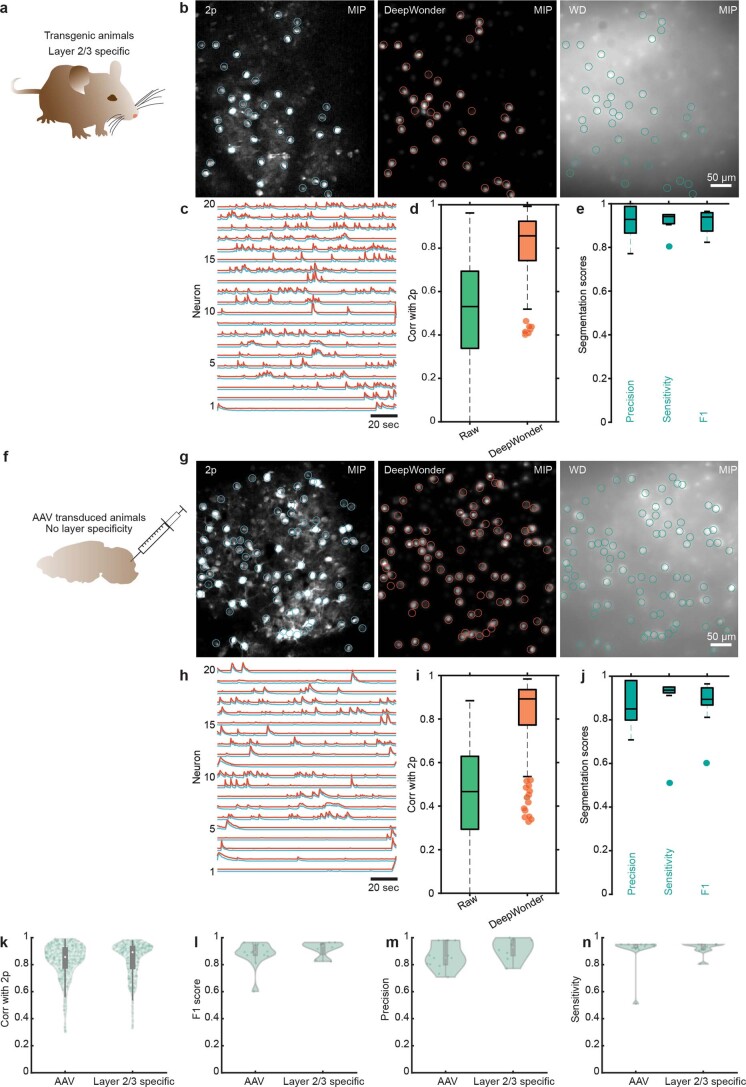
Validation of DeepWonder on layer 2/3-specific animals and AAV-transduced animals without layer 2/3-specificity. **a**. Simplified schematics of imaging transgenic animals with only layer 2/3 labeled. **b**. Left to right, maximum intensity projection (MIP) image across temporal frames of 2p movie, DeepWonder processed movie, and raw widefield movie. Paired active neurons co-detected in both 2p and DeepWonder movies are labeled by circles in each panel, where blue for 2p movie, red for DeepWonder movie, and green for raw movie. **c**. Neuronal activity traces of randomly selected 20 neurons in MIP panels as used for performance quantifications. Red traces for DeepWonder movie and blue traces for 2p movie, and DeepWonder traces are offset vertically for clarity. **d**. Temporal correlations of detected neurons with 2p from DeepWonder output movie (0.84 ± 0.13, mean ± SD, red) and raw movie (0.45 ± 0.21, mean ± SD, green), across n = 481 neurons from 8 recordings over 2 mice. Central black mark: Median. Bottom and top edges: 25th and 75th percentiles. Whiskers extend to extreme points excluding outliers (1.5 times above or below the interquartile range). **e**. Precision, sensitivity, and F1 scores of segmentation by DeepWonder are 0.92 ± 0.08, 0.92 ± 0.05, and 0.92 ± 0.06 across n = 8 recordings from 2 mice, respectively. Box plot elements as in **d**. **f**. Simplified schematics of imaging AAV-transduced animals without layer 2/3-specificity. **g**, **h** are the same as **b**, **c** but from AAV-transduced imaging data. **i**. Temporal correlations of detected neurons with 2p from DeepWonder output movie (0.83 ± 0.13, mean ± SD, red) and raw movie (0.50 ± 0.25, mean ± SD, green), across n = 650 neurons from 13 recordings over 3 mice. Box plot elements as in **d**. **j**. Precision, sensitivity, and F1 scores of segmentation by DeepWonder are 0.86 ± 0.10, 0.91 ± 0.12, and 0.88 ± 0.10 across n = 13 recordings from 3 mice, respectively. **k-n**. Distributions of temporal correlation, F1, precision, and sensitivity scores of DeepWonder-extracted traces in AAV-transduced animals (left) and layer 2/3-specific transgenic animals (right), respectively. Statistical scores are shown in mean ± SD. Data is collected from **d**, **e**, **i**, and **j**. White circle: Median. Thick grey vertical line: Interquartile range. Thin vertical lines: Upper and lower proximal values. Transparent disks: Data points. Transparent violin-shaped areas: Kernel density estimate of data distribution. Scar bar: 50 μm and 20 seconds.

To demonstrate that DeepWonder generalizes well in brain-wide widefield imaging, we used our hybrid microscopy strategy to evaluate the performance of DeepWonder across multiple cortical regions, structures and depths in multiple animals. DeepWonder achieved equally high correlation scores, segmentation scores and calcium event detection F1 scores across positions spread out over 5 mm in anterior–posterior direction (Extended Data Fig. 6a,b,g and Supplementary Fig. 15) and across similarly spread out medial–lateral positions (Extended Data Figs. 6c,d,h and Supplementary Fig. 16) from five animals. The mean correlation scores of DeepWonder across nine cortical regions was 0.82 ± 0.18 (mean ± s.d., *n* = 9 cortical regions; Fig. 2j), and the accuracy (F1 score) of neuron segmentation was 0.89 ± 0.02 (mean ± s.d., *n* = 9 cortical regions; Fig. 2k). DeepWonder also has reliable performance near vessels (correlation score 0.83 ± 0.09, mean ± s.d., *n* = 121 neurons; Supplementary Fig. 17) or complex vascular structures (correlation score 0.84 ± 0.11, mean ± s.d., *n* = 64 neurons; Extended Data Fig. 7). Across cortical depths, DeepWonder achieved correlation scores of 0.81 ± 0.18 (mean ± s.d., *n* = 1,483 neurons), calcium event detection F1 scores of 0.81 ± 0.11 (mean ± s.d., *n* = 1,483 neurons), and segmentation F1 scores of 0.87 ± 0.11 (mean ± s.d., *n* = 29 recordings) in *z* = 100–200 µm under the cranial window (Extended Data Figs. 8 and 9). Even in *z* = 250 µm, DeepWonder achieved passable performance with correlation scores of 0.68 ± 0.21 (mean ± s.d., *n* = 69 neurons), which was significantly better than CNMF-E (0.58 ± 0.26, mean ± s.d.; **P = 0.03, two-sided Wilcoxon signed-rank test; Supplementary Fig. 18). In data acquired from densely labeled tissue through virus transduction, DeepWonder also showed no compromise in performance with correlation scores of 0.83 ± 0.13 (mean ± s.d., *n* = 650 neurons) and segmentation F1 scores of 0.88 ± 0.10 (mean ± s.d., *n* = 13 recordings, Extended Data Fig. 10). Compared with an end-to-end artificial neural network that is trained to directly map widefield frames to background-free frames using data from the proposed hybrid system, DeepWonder driven by virtual calcium recordings exhibits higher correlation scores (0.85 ± 0.12 compared with 0.74 ± 0.22, mean ± s.d., *n* = 260 neurons; Supplementary Fig. 24).

### DeepWonder effectively removes background contamination in multi-region recordings

**Fig. 3:**
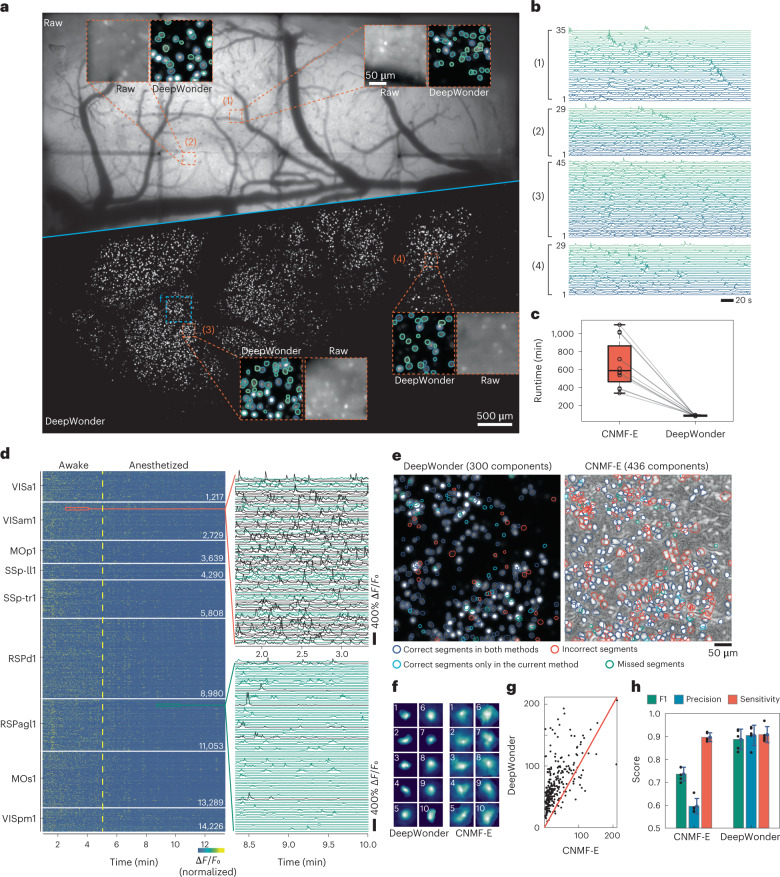
DeepWonder realizes high-speed processing of widefield neuronal recordings at terabytes scale. **a**, MIP of raw RUSH video (top) and background-corrected movie by DeepWonder (bottom). Orange dashed boxes mark zoom-in four areas in the cortex, with DeepWonder segmentation overlaid. The mean background value for widefield and DeepWonder are 0.58 and 0.0004, respectively (normalized by maximum value). **b**, Inferred calcium activities from four different areas marked by the dashed box with neuron number labeled. **c**, Runtime comparisons between CNMF-E and DeepWonder across 12 FOVs in RUSH over 8,000 frames recordings. Each dot shows the processing time for each FOV. Central black mark: median. Bottom and top edges: 25th and 75th percentiles. Whiskers extend to extreme points excluding outliers (1.5 times above or below the interquartile range). Black circles: *n* = 12 FOVs. **d**, Temporal activity rendering of 14,226 neurons inferred by DeepWonder in a 13.5 min recording. Two zoom-in panels show example traces (each with 100 traces). The dashed yellow line indicates the dosage of 2% isoflurane for anesthesia at 5th minute after the secession start. **e**, The contour plot of all neurons detected by DeepWonder (left) and CNMF-E (right) superimposed on the s.d. of background-corrected images and correlation image, respectively. Compared with manual segmentation, deep blue circles mark correct segments in both methods, red circles mark incorrect segments in each of the methods, green circles mark missed segments, and shallow blue circles mark correct segments that appear only in the current method. **f**, Spatial components of ten example neurons detected by both DeepWonder (left) and CNMF-E (right). **g**, The SNR of all neurons detected by DeepWonder (vertical axis) and CNMF-E (horizontal axis) in **e**. **h**, F1, precision and sensitivity scores of segmentation in **e** by CNMF-E are 0.74 ± 0.06, 0.58 ± 0.07 and 0.88 ± 0.04, respectively. F1, precision and sensitivity scores of segmentation by DeepWonder are 0.87 ± 0.10, 0.91 ± 0.09 and 0.88 ± 0.07, respectively. Statistical scores are shown in mean ± s.d. across *n* = 5 recordings in a single animal. Height of bars: mean. Error bars: s.d. Black dots: *n* = 5 recordings. Scale bars, 500 µm (**a**), 50 µm (zoom-in areas of **a**), 20 s (**b**) and 50 µm (**e**). Source data

The computational efficiency of DeepWonder enables us to process cortex-wide neuronal recording within acceptable time frames, which we demonstrate on data acquired with the terabytes-scale RUSH system^20^. The RUSH system consists of tens of scientific complementary metal-oxide semiconductor (sCMOS) cameras, with a total of 14,800 × 15,200 pixels across 10 × 8 mm^2^ FOV in 0.8 µm sampling size at video rate, allowing population-scale neuron connection inference. We simulated lifelike neuron recordings based on optical parameters of the RUSH system (Supplementary Fig. 1), and trained DeepWonder for the data modality of the RUSH system. With DeepWonder, neurons that were hidden in highly fluctuating backgrounds were clearly discernible (Fig. 3a and Supplementary Video 3), and high-contrast calcium transients were uncovered (Fig. 3b) thanks to effective background suppression (Extended Data Fig. 3 and Supplementary Fig. 19a,b). The high data throughput by the RUSH system yielded over 1 TB of data in a 13.5 min imaging session at 10 Hz. Processing such a dataset with the CNMF-E technique took over 5 days to fully demix neuron activities (132.4 h in total, without counting the loading time; Fig. 3c). In contrast, with DeepWonder, data of the same scale can be analyzed and inferred within 17 h. Up to 14,226 neurons across nine cortical areas were found with clearly discernible activities (Fig. 3d), showing potential for interrogating behavior-related neuron population response spanning over multiple cortical regions. When the awake mouse was anesthetized in the fifth minute with 2% isoflurane^37^, we observed that neurons gradually became inactive across different cortical regions with different dynamics (Fig. 3d and Supplementary Fig. 20). We further manually annotated neurons in a small FOV (~450 µm × 450 µm), and found DeepWonder achieved superior neuron segmentation compared with CNMF-E (Fig. 3e). The neurons segmented by DeepWonder were more concentrated in round shapes compared with those segmented by CNMF-E (Fig. 3f and Supplementary Fig. 19i), and the extracted calcium activities exhibited higher signal-to-noise ratios (SNRs; Fig. 3g and Supplementary Fig. 19h). DeepWonder achieved 0.87 ± 0.10 F1 scores in finding valid neurons compared with 0.74 ± 0.06 (mean ± s.d., *n* = 5 recordings; Fig. 3h and Supplementary Fig. 19e) by CNMF-E.

DeepWonder is also designed to be a general technique that can be compatible with various widefield calcium imaging systems. In a macroscope with a photographic lens as the objective^19^, neurons were largely undersampled by ~5 × 5 pixels laterally as a tradeoff for achieving a multi-millimeter FOV. We simulated hyperrealistic neuron recordings based on magnification, NA and other optical parameters of the macroscope system (Supplementary Fig. 2) to train DeepWonder. We found DeepWonder effectively reduced fluctuating backgrounds and segmented neurons efficiently (Supplementary Fig. 21a,b and Supplementary Video 4). DeepWonder achieved 0.88 F1 scores compared with 0.81 by CNMF-E with manual labeling as ground truth (Supplementary Fig. 21c,d). Neurons found by DeepWonder exclusively showed high-contrast calcium dynamics and compact shapes (Supplementary Fig. 21f). We further conducted hippocampal imaging through a glass pillar that periscoped the CA1 surface area to the objective focal plane for detection^34^ in the hybrid widefield and two-photon system (Supplementary Fig. 22a). DeepWonder faithfully uncovered neurons that were largely blurred in the raw widefield movie but detected by two-photon microscope (Supplementary Fig. 22b). Even though the CA1 neurons have different morphology compared with cortical neurons that were used to train DeepWonder, DeepWonder accomplished neuron detection and extraction with 0.81 ± 0.19 (mean ± s.d., *n* = 232 neurons) correlation scores compared with 0.60 ± 0.22 by the raw movie (Supplementary Fig. 22d), and 0.89 ± 0.07 F1 score in segmentation (Supplementary Fig. 22e). The demonstrations across multiple modalities illustrate the potential of DeepWonder in analyzing various widefield neuronal recordings. To aid researchers, we further supply a pretrained DeepWonder model that can be quickly adapted to different conditions without compromise in performance (Supplementary Fig. 23 and Supplementary Note 3).

## Discussion

While the proposed hybrid system allows for an artificial neural network to be trained to transform widefield frames to two-photon frames, DeepWonder trained by virtual calcium recordings outperformed it for manifold reasons. Firstly, pixel-level alignment of widefield and background-free recording is crucial for algorithm training, which is readily guaranteed using synthetic data but difficult to achieve using two-photon data as labels. Secondly, shot-noise-contaminated two-photon ground truth pollutes training labels and degrades performance. On the other hand, synthetic datasets based on hyperrealistic tissue simulation and real imaging model remove noise from labels and make the algorithm more stable. More importantly, the cross-modality training approach requires a hybrid imaging system as described in the article that is complicated to build, cost unfriendly and even inapplicable in certain situations (for example, head-mounted microscope), whereas DeepWonder can be applied to any widefield system.

The synthetic data-fueled training scheme in DeepWonder can be generalized into various applications. By modifying the synthetic recordings, the DeepWonder concept is also positioned to analyze the functional signals acquired with other indicators^38^. Analogously, reinforcing DeepWonder with volumetric imaging models such as light-field microscopy^39^ and multifocus microscopy^40^ enables inferring volumetric neuronal activities at high speed. On the other hand, utilizing generative adversarial networks for enhancing the virtual data generation holds potential to further improve the performance of DeepWonder^41^. We anticipate that our method lowers the barrier of processing neuronal data by high-throughput and large-scale widefield microscope, and promotes whole brain and million-level neuronal recordings and analysis.

## Methods

### One-photon and two-photon joint validation

To valid our algorithms in achieving correct neuronal activities, we built a joint two-photon and widefield detection system. The system was based on standard TPLSM, while we further added a 470-nm-centered widefield illumination path and a camera detection path in the system. The schematic of the custom-built two-photon microscope is shown in Extended Data Fig. 5. A titanium-sapphire laser system (MaiTai HP, Spectra-Physics) served as the two-photon excitation source (920 nm central wavelength, pulse width <100 fs, 80 MHz repetition rate). A half-wave plate (AQWP10M-980, Thorlabs) and an EOM (350-80LA-02, Conoptics) were used to modulate the excitation power. A 4f system (AC508-200-B and AC508-400-B, Thorlabs) with a 2× magnification was used to expand the laser beam to a resonant scanner (8315K/CRS8K, Cambridge Technology). The scanned beam went through a scan lens (SL50-2P2, Thorlabs) and a tube lens (TTL200MP, Thorlabs) and formed a tight focus through a high-NA water immersion objective (25×/1.05 NA, XLPLN25XWMP2, Olympus). A high-precision piezo actuator (P-725, Physik Instrumente) drove the objective for fast axial scanning. To match the two-photon excitation range with the widefield detection range, we reduced the beam size at the back aperture of the objective with an iris. The effective excitation NA was about 0.27 in our imaging experiments, yielding ~20 µm axial range (Supplementary Fig. 10). A long-pass dichroic mirror (DMLP650L, Thorlabs) was used to separate fluorescence signals from femtosecond laser beam by reflecting the fluorescence signals and transmitting the infrared laser light.

For the widefield excitation path, a long-pass dichroic (DMLP505L, Thorlabs) in the original detection path of TPLSM was used to send blue LED light (M470L4-C1 and MF475-35, Thorlabs) to the objective. To jointly record widefield excitation and two-photon excitation, a 50:50 (reflectance:transmission) nonpolarizing plate beam splitter (BSW27, Thorlabs) was placed after the widefield dichroic to separate fluorescent signals for PMT (PMT1001, Thorlabs) and camera (Zyla 4.2, Andor), respectively. A pair of fluorescence filters (MF525-39, Thorlabs; ET510/80M, Chroma) was configured in front of both the PMT and the camera to fully block both femtosecond laser and widefield excitation beam. The back aperture of the objective was optically conjugated to the detection surface of the PMT with a 4f system (TTL200-A and AC254-050-A, Thorlabs).

To avoid excitation crosstalk and protect PMT from high-flux widefield emission photons, we added a linear galvo that served as an optical shutter for the PMT detection path, which deflected widefield fluorescent photons when LED was on (Extended Data Fig. 5a). We further configured the EOM to be blocked during widefield imaging. The LED (M470L4-C1) was in trigger mode with a typical rising and falling time less than 1 ms, with further reduced duration time to avoid PMT overexposure (Extended Data Fig. 5b). To further validate the correctness of DeepWonder signals, we modified our hybrid system with high-NA two-photon excitation (NA 0.6). The optical setup was similar to the low NA (NA 0.27) in Extended Data Fig. 5, but the beam expansion after the EOM was increased for achieving high-NA point spread function (PSF). Both high-NA two-photon and widefield captures were at 15 Hz. The calibrated high-NA two-photon excitation had a 1/e axial PSF width of 5 µm, compared with 25 µm by a NA 0.27 excitation PSF.

### Realistic widefield capture generation

To synthesize a realistic cortical tissue and generate corresponding widefield capture, we referred to the Neural Anatomy and Optical Microscopy (NAOMi)^15^ package. Using NAOMi, a brain tissue volume was populated with multiple blood vessels, as well as with neuron somata, axons and dendrites. Neurons and dendrites were assigned synthesized fluorescence activity that reflected their calcium dynamics. A tissue-specific PSF was generated by layer-to-layer Fresnel propagations from deep tissue to the camera sensor.

While original NAOMi was used to simulate two-photon excitations, here we modified the original NAOMi pipeline such that it could faithfully simulate data acquisition of one-photon excitations, which was termed as NAOMi1p. We changed the excitation wavelength from the near-infrared range into the visible range. In two-photon microscope, scattering-induced aberrations in the excitation beam instead of the emission beam affect the imaging quality due to the point-scanning manner. Contrastingly, in widefield microscope, scattering-induced aberrations cause troubles in emission paths instead of excitation paths due to the planar collection from different camera pixels. We thus modified the optical PSF generation on the basis of the propagation of the emission beam instead of the excitation beam through the tissue. We further replaced the two-photon absorption process with one-photon absorption process in a model of power density, fluorescent concentration, extinction coefficient, quantum yield and fluorescent protein expression level^43^. The final simulated recordings have three contributors: fluorescence from active neurons, fluorescence from dendrites and axons in the background, and fluorescence from out-of-focus backgrounds. The assembly of all three parts faithfully generates a virtual capture of widefield recordings, while using fluorescence from active neurons only generates a background-free label. Especially, for soma target indicators^44^ it is recommended to let only the soma fire. The above tools are summarized as the NAOMi1p toolbox and are open to all the community. To accommodate different imaging systems, NAOMi1p opens multiple parameters including the acquisition NA, camera pixel size, magnifications, illumination power, FOV and indicator types for users to adjust. To control the distributions of the pixel histogram of the NAOMi1p output to be similar with experimental data, the number and the peak activity of neuropils were adjusted, which effectively modulates histograms but did not disturb neuronal dynamics. A linear mapping was further conducted such that the position and spread of the histograms were similar to the experimentally captured data. To equalize the distribution of Δ*F*/*F* between experimental data and NAOMi1p output, we firstly calculated the background Δ*F*/*F* histogram of an experimental video (MATLAB function histogram) as a reference. We then controlled the spike number of each neuropil candidate to match that reference histogram. With these adjustments, the output Δ*F*/*F* and also the maximum Δ*F*/*F* value distributions could be highly similar to experimental data.

We notice that some cortical regions have rich vascular populations, which might disturb neuronal extraction by DeepWonder^45^. At the statistical level, we have proven that DeepWonder achieves satisfactory performance on regions that contain blood vessels (Extended Data Fig. 7 and Supplementary Fig. 17). However, we found that there was a slight drop of correlation scores when the neuron–vessel distance is smaller than 20 µm (0.74 ± 0.15 correlation score when neuron–vessel distance is near zero, compared with 0.85 ± 0.13 correlation score when neuron–vessel distance is 30 µm; Supplementary Fig. 25c). Note the portion of neurons that are within that affected ranges are small in the all inferred neuron populations (36/492 ≈ 7.3%, summarized from *n* = 4 datasets). On the other hand, to further increase the performance of DeepWonder in the conditions that neurons are very close to vessels (for example, neuron–vessel distance is smaller than 20 µm), we incorporated hemodynamics modeling in NAOMi1p. We introduced random dilations of vessels during virtual widefield capture simulation (Supplementary Fig. 25d,e). The dilations are varied in different FOV positions to mimic the physical vessel movement. The vessel-aware NAOMi1p model enables DeepWonder to achieve better performance on neurons that are close to vessels (Supplementary Fig. 25g), but takes longer time to generate training data.

With NAOMi1p, we can faithfully generate virtual widefield recordings as well as their background-free counterpart. We then picked up neurons that were within the range of axial PSF diameter (Gaussian beam, 1/e^2^ size) and registered their positions and activities as ground truth for simulation comparisons among different analysis algorithms (Supplementary Note 1).

### Noise simulation

Imaging sensors (for example, sCMOS, CMOS and charge-coupled device) have different quantum efficiency and noise response, which is also highly coupled with the expression level of calcium indicators in neurons. We thus simulated the NAOMi1p data with a range of noise to cover those situations. The number of fluorescence photons generated in a unit area of the samples is^43^

$$N_p \propto Q \cdot {\it{\epsilon }} \cdot F\left( {x,y} \right) \cdot P \cdot \tau$$

where *Q* is the quantum efficiency of fluorophores with an extinction coefficient ${\it{\epsilon }}$, *F*(*x*,*y*) is the local fluorophore concentration, *P* is power density and *τ* is the integration time of the camera. The signal of a camera can be further interpreted as^46^

$$Z_p = \gamma _p{{{\mathrm{Pois}}}}\left\{ {N_p} \right\} + N\left( {0,\;\sigma _R} \right) + \beta _p$$

where *γ*_*p*_ is a multiplicative factor which is applied to the Poisson distribution (Pois) as the camera gain. $\beta _p$ is a bias during analog-to-digital conversion. $N(0,\sigma _R)$ is the Gaussion-distributed readout noise with zero mean and $\sigma _R$ standard deviation. For a typical sCMOS, $\gamma _p$ is ~2.2, $\beta _p$ is ~100 and $\sigma _R$ is ~200 (ref. ^47^).

### Widefield imaging setups and recordings

#### RUSH recordings

In the RUSH system^20^, a 5 × 7 customized field lens array was mounted on a spherical surface for full correction of field curvature of a 10 mm × 12 mm FOV. The customized objective provides 0.35 NA across the centimeter-scale FOV, supporting submicron resolution observation. The pixel resolution of each camera in RUSH system is 2,560 × 2,160, yielding 6.3 GB data per minute at 10 Hz. A mouse with a 7 mm cranial window takes 12 sub-FOVs of RUSH, and a 13.5 min recording take over 1 TB of data (Fig. 3 and Supplementary Figs. 19 and 20). To generate virtual recordings for DeepWonder training, we fed the following typical parameters to the data generator: system magnification 10, NA 0.35, pixel size 0.8 µm, frame rate 10 Hz and illumination power density 0.8 mW mm^−2^. We evaluated the similarity of generated data with raw recordings in terms of pixel histogram, functional fluctuation histogram and spatial frequency distribution (Supplementary Fig. 1).

#### Macroscope recordings

We used a 50 mm camera lens (Canon EF 50mmf/1.4 USM) as the objective lens and a 100 mm camera lens (MINILTA AF 100mmf/2.8) as the tube lens to set up the widefield macroscope. The illumination was provided by a collimated blue LED (SOLIS-470C, Thorlabs) with an excitation filter (FESH0500, Thorlabs). The beam was focused by a lens (AC508-100-A, Thorlabs), reflected by a dichroic mirror (DMLP505L, Thorlabs), passed through the objective lens and excited the sample. The fluorescence was collected by the same objective lens and refocused on the sCMOS camera (Zyla 5.5, Andor) by the tube lens. An emission filter (MF525-39, Thorlabs) was placed before the camera to eliminate the excitation light. The FOV of the system was approximately 9.2 mm × 7.7 mm, and each pixel in the sCMOS corresponded to 3.6 µm on the image plane. To generate virtual recordings for DeepWonder training, we fed the following typical parameters to the data generator: system magnification 1.8, NA 0.3, pixel size 3.6 µm, frame rate 10 Hz and illumination power density 0.8 mW mm^−2^. We evaluated the similarity of generated data with raw recordings in terms of pixel histogram, functional fluctuation histogram and spatial frequency distributions (Supplementary Fig. 2).

### Network architecture and training

#### RB-Net

The main structure of the RB-Net is 3D Unet. The encoding path and decoding path consist of three convolutional blocks (Extended Data Fig. 1a). For accelerating removing background process, we added a ‘spatial to channel’ downsampling operator^48^ at the beginning of RB-Net for reshaping the input image of size *W* × *H* × *C* into *W*/2 × *H*/2 × 4*C* (*W* for filter width, *H* for filter height and *C* for filter channels; Extended Data Fig. 1c). We also introduced a ‘channel to spatial’ upsampling operator at the end of RB-Net for realigning pixels (Extended Data Fig. 1c). With these two operators, the pixel number of an input image processed by RB-Net can be increased by four times at almost the same graphics processing unit (GPU) memory cost (Supplementary Fig. 3a,b). We utilized a linear transformation of raw input images *x* for data augmentation as

$$y = \gamma \cdot \left( {x + \beta } \right),$$

where *y* is input images for RB-Net, *γ* and *β* are random number ($0.2 < \gamma < 2$,$0 < \beta < {\mathrm{max}}(x)$). The stride size *l* of ‘spatial to channel’ and ‘channel to spatial’ operators is crucial for performance, where *l* × *l* pixels from the input are realigned into *l*^2^ channels. The stride size *l* was optimized to achieve the best performance (*l* = 2; Supplementary Fig. 3c,f). We found that the ‘spatial to channel’ operator had a superior performance compared with a large convolutional filter with a large stride, even computing time of both approaches are similar (Supplementary Fig. 26). Data augmentation was constructive to the generalization ability and transfer learning ability of RB-Net.

We synthesized 23 sets of background-removed data by the NAOMi1p algorithm and randomly split them into 4,000 paired patches for training RB-Net. The input raw videos were mean subtracted. It took 48 h to train RB-Net for 30 epochs with a Geforce RTX 3080 GPU. The running speed for RB-Net is usually 40 ms per 750 × 750-pixel frame tested in an RTX 3080 GPU.

#### NS-Net

The main structure of the NS-Net is 3D Unet, which has the similar structure with the RB-Net but with different channels (Extended Data Fig. 1b). On the other hand, because neuron segmentation in background-free data is simpler than removing background, we utilized the combination of a 1 × 1 × 3 filter and a 3 × 3 × 1 filter in NS-Net to replace two 3 × 3 × 3 filters for reducing network parameters and computational consumption.

The training data for NS-Net were directly generated from NAOMi1p generator, where neuron soma that was within the range of axial PSF diameter (Gaussian beam, 1/e^2^ size) was binarized as the segmentation label. We simulated 45 sets of neuron segmentation data and randomly generated 4,000 paired patches for training NS-Net. We spent 8 h training NS-Net for 30 epochs with a Geforce GTX 1080TI GPU.

### Processing of widefield calcium data

Widefield calcium recordings were firstly sent to trained RB-Net to get a de-background clean movie, then the background-free movie was further sent to trained NS-Net for acquiring neuron candidate masks (Extended Data Fig. 2a). We grouped and merged candidates from all frames into connected regions to form unique segments. We then conducted the connectivity analysis for every candidate of the mask sequence spatio-temporally and extracted every separated neuron to compose a neuron candidate list. Those spatially overlapped but temporally separated (for example, neuron segments appear in different frames) were registered as different candidates. With the neuron candidate list, we classified these neurons by neuron morphology metrics related to area and roundness $\theta = 4\pi \cdot \frac{s}{{p^2}}$, where *s* is the area of neuron and *p* is the perimeter of neuron. We abandoned the neuron candidates that were smaller than the 25 µm^2^ threshold. Since the roundness *θ* is a good indicator to judge if the candidate consists of a single neuron or multiple neurons, we further classified neuron candidates whose roundness were higher than the standard roundness of a single neuron (typically *θ* = 0.8) to form a ‘good’ neuron list, and others into a ‘bad’ neuron list (Extended Data Fig. 2b). The candidates in the ‘good’ neuron list were sent out for directly reading out temporal activities from the background-removed movie based on values of exclusive pixels (Extended Data Fig. 2a). For each candidate in the ‘bad’ neuron group, the candidate was initialized by greedy methods^24^ and then sent to local NMF for further demixing. If we marked the local area surrounding the candidate as $Y \in R^{d_1 \times d_2 \times T}$, and the candidate was estimated to be consisted by *K* neurons, the local NMF model was then

$$\mathop {{\min }}\limits_{A,C} Y - AC_F^2 + \alpha _AA_1 + \alpha _CC_1,$$

where $A \in R^{d_1 \times d_2 \times K}$ and $C \in R^{K \times T}$ represented the spatial and temporal footprints, respectively^24^. We solved the above optimization problem through the hierarchical alternating least squares algorithm^35^. Finally, we merged neurons by clustering components with high temporal correlations and spatial overlap ratios. We compared DeepWonder with various widefield neuron extraction and activity inference methods (parameter settings can be found in Supplementary Note 1). All methods were run in the same machine, which has an Intel I9-9980Xe central processing unit, 128 GB random-access memory and an RTX 3080 GPU.

### Mouse preparation and calcium imaging

All animal experiments were performed following institutional and ethical guidelines for animal welfare and have been approved by the Institutional Animal Care and Use Committee of Tsinghua University. Mice were housed in cages (24 °C, 50% humidity) in groups of one to five under a reverse light cycle. Both male and female mice were used without randomization or blinding.

We used Rasgrf2-2A-dCre mice (JAX 022864) crossed with Ai148 (TIT2L-GC6f-ICL-tTA2)-D (JAX 030328) transgenic mice for most of cortical functional imaging. Adult transgenic mice at 8–12 postnatal weeks were anesthetized with 1.5% isoflurane, and craniotomy surgeries were conducted with a stereotaxic instrument (68018, RWD Life Science) under a bright-field binocular microscope (77001S, RWD Life Science). A custom-made coverslip fitting the shape of the cranial window was cemented to the skull. A biocompatible titanium headpost was then cemented to the skull for stabilization during imaging. The edge of the cranial window was enclosed with dental cement to hold the immersion water of the objective. After the surgery, trimethoprim was injected into the mice intraperitoneally for inducing the expression of GCaMP6f in layer 2/3 neurons (0.25 mg g^−1^). To reduce potential inflammation, 5 mg kg^−1^ (body weight) of ketoprofen was injected subcutaneously. Each mouse was housed in a separate cage for 1–2 weeks of postoperative recovery.

We used Rasgrf2-2A-dCre mice (JAX 022864) crossed with Ai148 (TIT2L-GC6f-ICL-tTA2)-D (JAX 030328) transgenic mice with adeno-associated virus injection for both cortical and hippocampal imaging (Supplementary Fig. 22). We prepared the mice using the same procedures as above, except (1) the cortex matter (1.5 mm distant from the sagittal suture and 2 mm distant from the lambdoid suture) was aspirated via a 0.9-mm-diameter (19 gauge) blunt needle that was connected to a vacuum pump^34^; (2) we injected cocktail of AAV2/9-hSyn-FLEX-GCaMP6f-WPRE-pA and AAV2/9-hSyn-Cre-WPRE-pA into the hippocampal area; (3) a chronic window with a glass pillar (0.9 mm in thickness and 2 mm in diameter) attached was implanted above the cortex, and the pillar sit directly above the hippocampal CA1 area^34^ instead of the flat coverslip.

We used adeno-associated virus transduced C57BL/6J mice for verification of the generalization ability of DeepWonder (Extended Data Fig. 10). We prepared the mice using the same procedures as the above transgenic mice, except (1) expression was achieved through injection of a genetically expressed calcium indicator adeno-associated virus (AAV1-hSyn1-GCaMP6f) at ~1–2 weeks before cranial window implantation (ten sites with 400 μm spacing at a depth of 300 µm below the dura, 25 nl for each site, titer ~10^12^ viral particles ml^−1^); (2) no trimethoprim was injected into the mice for inducing layer-2/3-specific expression.

Imaging experiments were carried out when the cranial window became clear and no inflammation occurred. Mice were first rapidly anesthetized with 3.0% isoflurane and then fixed onto a custom-made holder by the headpost. A precision three-axis translation stage (M-VP-25XA-XYZL, Newport) carried the mice for a proper ROI. For two-photon validation experiments, the correction ring of the 25× water immersion objective was adjusted to compensate for the coverslip thickness and eliminate spherical aberrations. The highest excitation power of two-photon microscope after the objective was under ~100 mW to avoid heat damage. During the imaging session, gaseous anesthesia was turned off and the mouse was kept awake. For widefield acquisitions, the excitation power density in the cranial window area was no more than 1.5 mW mm^−2^. Before running further analysis, we ran calcium movie registrations with open-source NormCorre algorithm^49^ to cancel motion artifacts. In cortex-wide brain imaging, we aligned the recorded brain area into Allen CCF atlas on the basis of the recorded position of the cranial window by the stereotaxic instrument when applying brain surgery.

### Performance metrics

#### Correlation score

We used Pearson’s correlation coefficient as the temporal metric to monitor the similarity between inferred neuronal activities and ground truths. The ground truth activities were available for simulation data, while for joint one-photon and two-photon validation data, the ground truth activities were established by running CaImAn^36^ on two-photon datasets (Supplementary Note 2).

#### Neuron finding scores

It is necessary to establish ground truth segmentation for comparing the neuron finding scores. In simulation data, the ground truth segmentation was readily available. In joint one-photon and two-photon validation data, the ground truth segmentation was established on the basis of CaImAn processed two-photon data (Supplementary Note 2). In widefield experimental data, we manually labeled the neurons on the basis of their positions and activities. We firstly calculated the correlation images of the raw recordings^36^, and worked over every structure that was different from the background and matched the neuron size (typically ~10–15 µm in diameter). We rejected candidates that with weak and noisy activities in the raw movie. We outlined each cell of interest with the ROI manager in ImageJ, and imported the zipped ROIs into MATLAB as ground truths for comparison with other methods.

After achieving segmentation ground truth, a customized script in MATLAB automatically evaluated segmentation by the following rules: a candidate is a correct segment (true positive, TP) if the minimal distance between this candidate with any ground truth segments is less than 8 µm, and the Intersect over Union score between this candidate and that ground truth segment is larger than 0.2. Otherwise, the segmentation candidate will be rejected as a false positive (FP). Segments appear in ground truth labeling but are not recognized by the algorithm will be marked as false negatives (FN). The segmentation accuracy (F score, F1) is defined as

$${\mathrm{F}}1 = \frac{{2{\mathrm{TP}}}}{{2{\mathrm{TP}} + {\mathrm{FP}} + {\mathrm{FN}}}}.$$

The segmentation precision score is defined as

$${\mathrm{Precision}} = \frac{{{\mathrm{TP}}}}{{{\mathrm{TP}} + {\mathrm{FP}}}}.$$

The segmentation sensitivity score is defined as

$${\mathrm{Sensitivity}} = \frac{{{\mathrm{TP}}}}{{{\mathrm{TP}} + {\mathrm{FN}}}}.$$

#### SBR

We calculated the SBR of a neuron by computing the maximum activity of the neuron area over the maximum activity of its neighboring area (Extended Data Fig. 3c) across all temporal frames. The neuron area was defined by a circle that had a radius of 10 µm and was centered at the centroid of a segment. A neighboring area was defined by a ring with an inner radius of 10 µm and an outer radius of 20 µm at the same center of the corresponding neuron area while masking out all other neuron areas.

#### SNR

We computed the SNR of inferred cellular traces to quantitatively compare the temporal inference quality^24^. We calculated the denoised trace *c* of each inferred activity *y* using OASIS^50^, and the SNR was computed through

$${\mathrm{SNR}} = \frac{{c_2^2}}{{y - c_2^2}}.$$

#### Calcium event detection scores

To achieve the quantification of calcium event restoration ability in experiments, we firstly deconvolved the calcium traces of DeepWonder-processed widefield recordings and CaImAn-processed 2p recordings using OASIS^50^, which output a spike train based on an autoregressive (AR) model:

$$c_t = \mathop {\sum }\limits_{i = 1}^p \gamma _ic_{t - i} + s_t,$$

where *c*_*t*_ is the calcium fluorescence intensity, *p* is the order AR model (*p* = 1 in this research), *γ*_*i*_ are AR parameters and *s*_*t*_ represents spikes. We labeled a 2p transient *s*_*t*_ was restored by DeepWonder if there was a DeepWonder spike near the 2p spike in a window of 300 ms, considering the relatively slow kinetics of the calcium indicator. We then calculated the precision, sensitivity and F1 score of calcium event restoration as defined above.

### Reporting summary

Further information on research design is available in the Nature Portfolio Reporting Summary linked to this article.

## Online content

Any methods, additional references, Nature Portfolio reporting summaries, source data, extended data, supplementary information, acknowledgements, peer review information; details of author contributions and competing interests; and statements of data and code availability are available at 10.1038/s41592-023-01838-7.

## Supplementary information

Supplementary Information Supplementary Figs. 1–26 and Notes 1–3.

Reporting Summary

Supplementary Software 1 DeepWonder and NAOMi1p computational pipeline, with inline documentation, and demo scripts. See https://github.com/yuanlong-o/Deep_widefield_cal_inferecefor for future updates.

Supplementary Video 1 Principles of DeepWonder. In DeepWonder, brain tissues are intensively simulated and controlled. By virtually generating background-contaminated and background-free captures, we train a neural network that separates neuronal signals from scattered background and neuropil signals. Guaranteed by the high similarity between virtual and real recordings, the trained DeepWonder network can effectively remove backgrounds of experimental recordings. Neurons are further segmented from background-removed movie, and temporal activities are inferred.

Supplementary Video 2 Validation of DeepWonder with joint one-photon and two-photon acquisitions. DeepWonder massively suppresses the background in widefield neuronal imaging, and is validated with joint two-photon capture as ground truth. Two example datasets across different animals are shown here.

Supplementary Video 3 Applying DeepWonder in RUSH recordings. DeepWonder effectively removes background in RUSH recordings in an FOV of 7 × 6 mm^2^ at 0.8 µm pixel size and segments 14,226 neurons in total.

Supplementary Video 4 Applying DeepWonder in macroscope recordings. DeepWonder effectively removes background in macroscope brain recordings in an FOV of 1.6 × 1.6 mm^2^ at 3.6 µm pixel size and segments 1,345 neurons in total.

## Data Availability

We have mounted our demo data and codes in Google Colab. A demo script with full processing of DeepWonder on several demo datasets (including NAOMi1p virtual datasets, cropped RUSH datasets and two-photon validation datasets) is available through Colab via https://colab.research.google.com/drive/15TvsyEYgE1iGpaNWkq3flXOw52I51mVa. Over 50 Gb paired 2p and widefield data have been made publicly available through https://drive.google.com/drive/folders/1OBcQUY-vsIPljSBChFfn-zqAYtYvDZ4A?usp=sharing. The Allen CCF atlas is available at http://atlas.brain-map.org. Source data are provided with this paper.

## Source data

Source Data Fig. 1 Statistical source data.
Source Data Fig. 2 Statistical source data.
Source Data Fig. 3 Statistical source data.

## Code availabIlity

Our DeepWonder with realistic widefield imaging simulators can be found at https://github.com/yuanlong-o/Deep_widefield_cal_inferece and is available in Supplementary Software 1 under an open source license permitting not-for-profit research use (see file LICENSE.txt). An archived version of DeepWonder packages is available through https://pypi.org/project/DWonder. Source data are provided with this paper.
