# Supplementary material for: Rapid detection of neurons in widefield calcium imaging datasets after training with synthetic data
Source: Nat Methods. 2023 Apr 1;20(5):747–54. doi: 10.1038/s41592-023-01838-7 (PMC10172132; doi:10.1038/s41592-023-01838-7)
Supplement: Supplementary file 1 — Supplementary Figs. 1–26 and Notes 1–3. [file 41592_2023_1838_MOESM1_ESM.pdf]

nature methods

Article

https://doi.org/10.1038/s41592-023-01838-7

Rapid detection of neurons in widefield calcium imaging datasets after training with synthetic data

In the format provided by the authors and unedited

Tables of contents

Supplementary Figures

**Supplementary Figure 1**	Comparison of simulated data to recordings of mouse motor cortex with the RUSH system.
**Supplementary Figure 2**	Comparison of simulated data to recordings of mouse motor cortex with a macroscope system.
**Supplementary Figure 3**	Evaluation of DeepWonder with different strides of S2C operation.
**Supplementary Figure 4**	Visualize the removing background process of RB-Net in DeepWonder.
**Supplementary Figure 5**	RB-Net in DeepWonder outperforms both ring-CNN and min1pipe.
**Supplementary Figure 6**	Local NMF in segmentation stage effectively resolves overlapped neurons.
**Supplementary Figure 7**	NS-Net in DeepWonder outperforms both CaImAn-Batch and SUNS in background-removed neuronal segmentation.
**Supplementary Figure 8**	Comparison of NS-Net in DeepWonder with STNeuroNet.
**Supplementary Figure 9**	DeepWonder functions robustly across different noise scales.
**Supplementary Figure 10**	Calibration of the joint 2p-widefield detection system.
**Supplementary Figure 11**	DeepWonder retrieves accurate calcium inference with joint widefield-2p detection (mouse #2).
**Supplementary Figure 12**	DeepWonder retrieves accurate calcium inference with joint widefield-2p detection (mouse #3).
**Supplementary Figure 13**	Validation of DeepWonder functional signals with high-NA ground truth.
**Supplementary Figure 14**	Validation of DeepWonder on brain wide with hybrid widefield and high-NA recordings.
**Supplementary Figure 15**	Validation of DeepWonder on different anterior-posterior positions in mouse cortex.
**Supplementary Figure 16**	Validation of DeepWonder on different medial-lateral positions in mouse cortex.
**Supplementary Figure 17**	Validation of DeepWonder encountering hemodynamic responses.
**Supplementary Figure 18**	Comparison of DeepWonder and CNMF-E in 200-300 μm deep mouse cortical imaging.
**Supplementary Figure 19**	Comparisons of DeepWonder and CNMF-E in RUSH dataset.
**Supplementary Figure 20**	Various wake-to-sleep latency distributions across multiple brain area.
**Supplementary Figure 21**	Comparisons of DeepWonder and CNMF-E in macroscope dataset.
**Supplementary Figure 22**	DeepWonder in cortical and hippocampal imaging.
**Supplementary Figure 23**	Fast fine tuning DeepWonder in new datasets through a pretrained model.
**Supplementary Figure 24**	Comparisons of a neural network trained by experimental paired hybrid data with DeepWonder.

**Supplementary Figure 25**	Incorporating hemodynamics modeling in NAOMi1p model.
**Supplementary Figure 26**	Comparison of S2C with a large convolutional filter and a large stride.

Supplementary Notes

**Supplementary Note 1**	Other widefield neuron extraction and activity inference methods.
**Supplementary Note 2**	Joint one-photon and two-photon data acquisition.
**Supplementary Note 3**	Utilizing pre-trained DeepWonder model for quickly adapting to other datasets.

Supplementary Videos

**Supplementary Video 1**	Principles of DeepWonder. In DeepWonder, brain tissues are intensively simulated and controlled. By virtually generating background contaminated and background-free captures, we train a neural network that separates neuronal signals from scattered background and neuropil signals. Guaranteed by the high similarity between virtual and real recording, the trained DeepWonder network can effectively remove backgrounds of experimental recordings. Neurons are further segmented from background removed movie and temporal activities are inferred.
**Supplementary Video 2**	Validation of DeepWonder with joint one-photon and two-photon acquisitions. DeepWonder massively suppresses the background in widefield neuronal imaging, and is validated with joint two-photon capture as ground truth. Two example datasets across different animals are shown here.
**Supplementary Video 3**	Applying DeepWonder in RUSH recordings. DeepWonder effectively removes background in RUSH recordings in a FOV of 7 x 6 mm^2 at 0.8 μm pixel size and segments 14226 neurons in total.
**Supplementary Video 4**	Applying DeepWonder in macroscope recordings. DeepWonder effectively removes background in macroscope brain recordings in a FOV of 1.6 x 1.6 mm^2 at 3.6 μm pixel size and segments 1345 neurons in total.

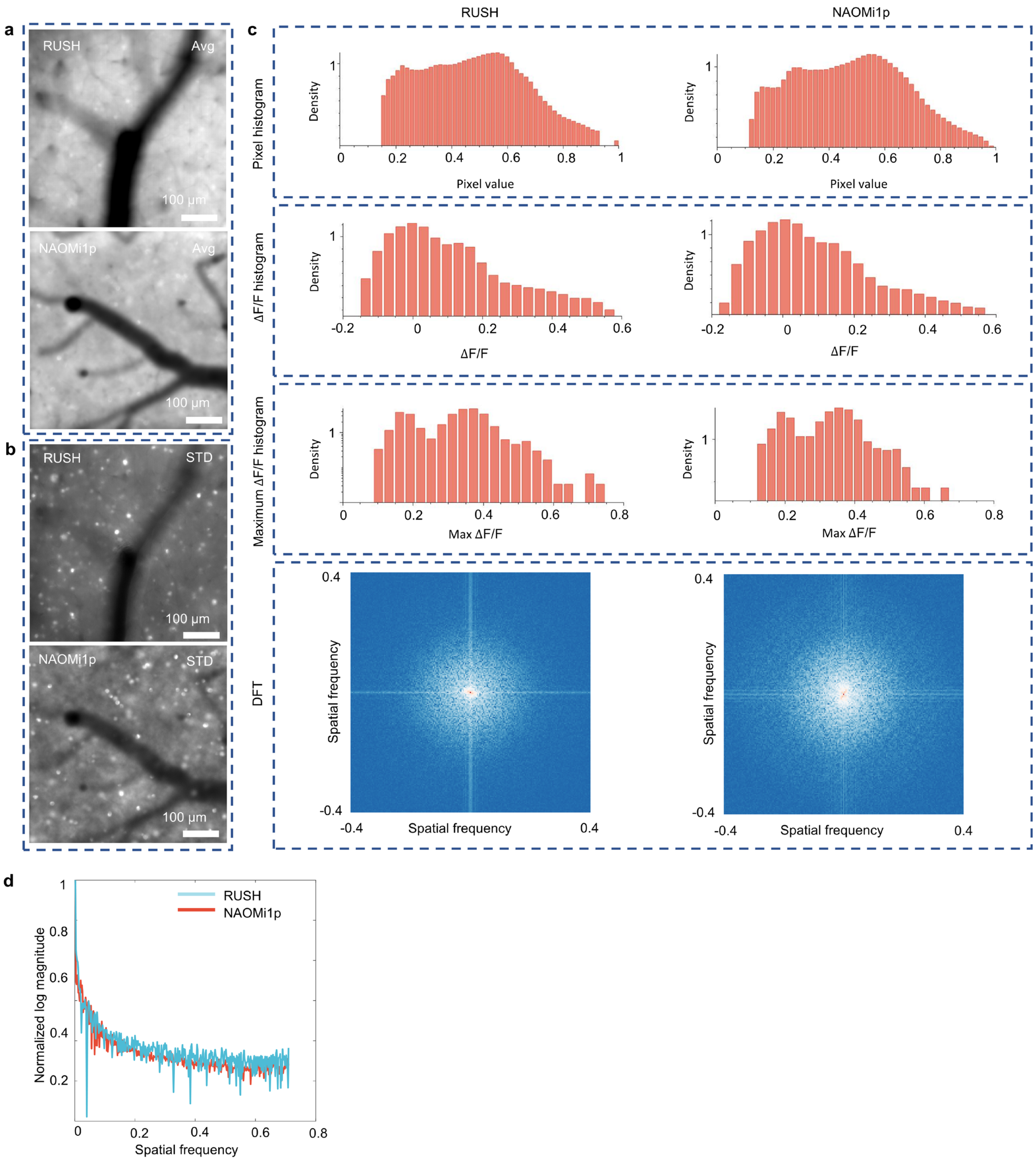

Supplementary Figure 1

Comparison of simulated data to recordings of mouse motor cortex with the RUSH system.

a. The averaged image (avg) for mouse recordings with the RUSH system (top) and simulated data with the proposed NAOMi1p (bottom).

b. The standard deviation image (STD) across time axis for mouse recordings with RUSH (top) and simulated data with proposed NAOMi1p (bottom).

c. Comparisons of pixel value distributions, ΔF/F values distributions, maximum ΔF/F values distributions, and spatial frequency distributions of the averaged image by RUSH recordings and simulated data by the proposed NAOMi1p. The histograms are all plotted in logarithmic scale.

d. The overall contributions at different spatial frequencies to the mean activity match between the recording (blue) and the simulation (red).

Scale bar: 100 μm.

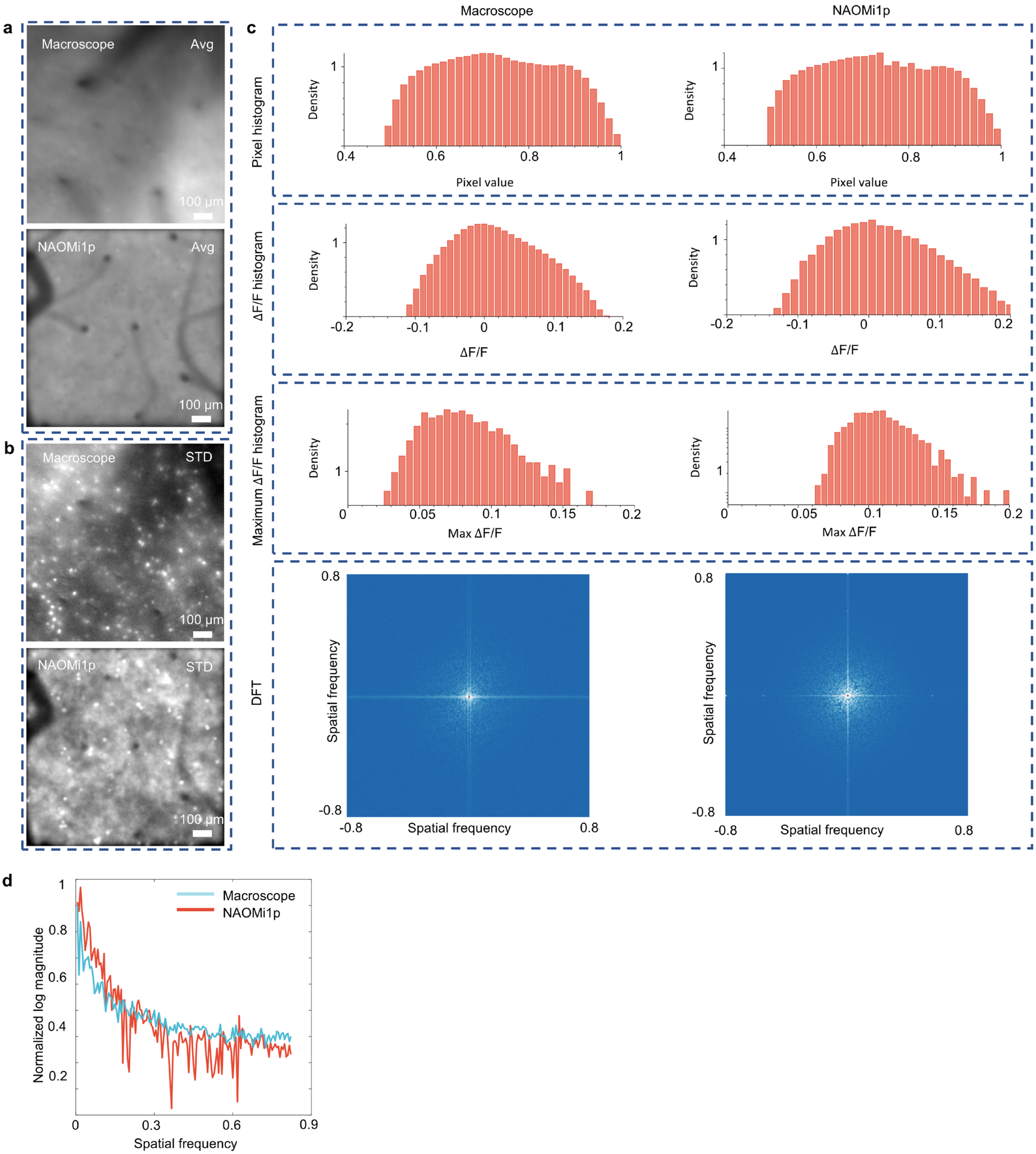

Supplementary Figure 2

Comparison of simulated data to recordings of mouse motor cortex with a macroscope system.

a. The averaged image (avg) for mouse recordings with a macroscope (top) and simulated data with the proposed NAOMi1p (bottom).

b. The standard deviation image (STD) across time axis for mouse recordings with a macroscope (top) and simulated data with proposed NAOMi1p (bottom).

c. Comparisons of pixel value distributions, ΔF/F values distributions, maximum ΔF/F values distributions, and spatial frequency distributions of the averaged image by widefield microscope recordings and simulated data by the proposed NAOMi1p. The histograms are plotted in logarithmic scale.

d. The overall contributions at different spatial frequencies to the mean activity match between the recording (blue) and the simulation (red).

Scale bar: 100 μm.

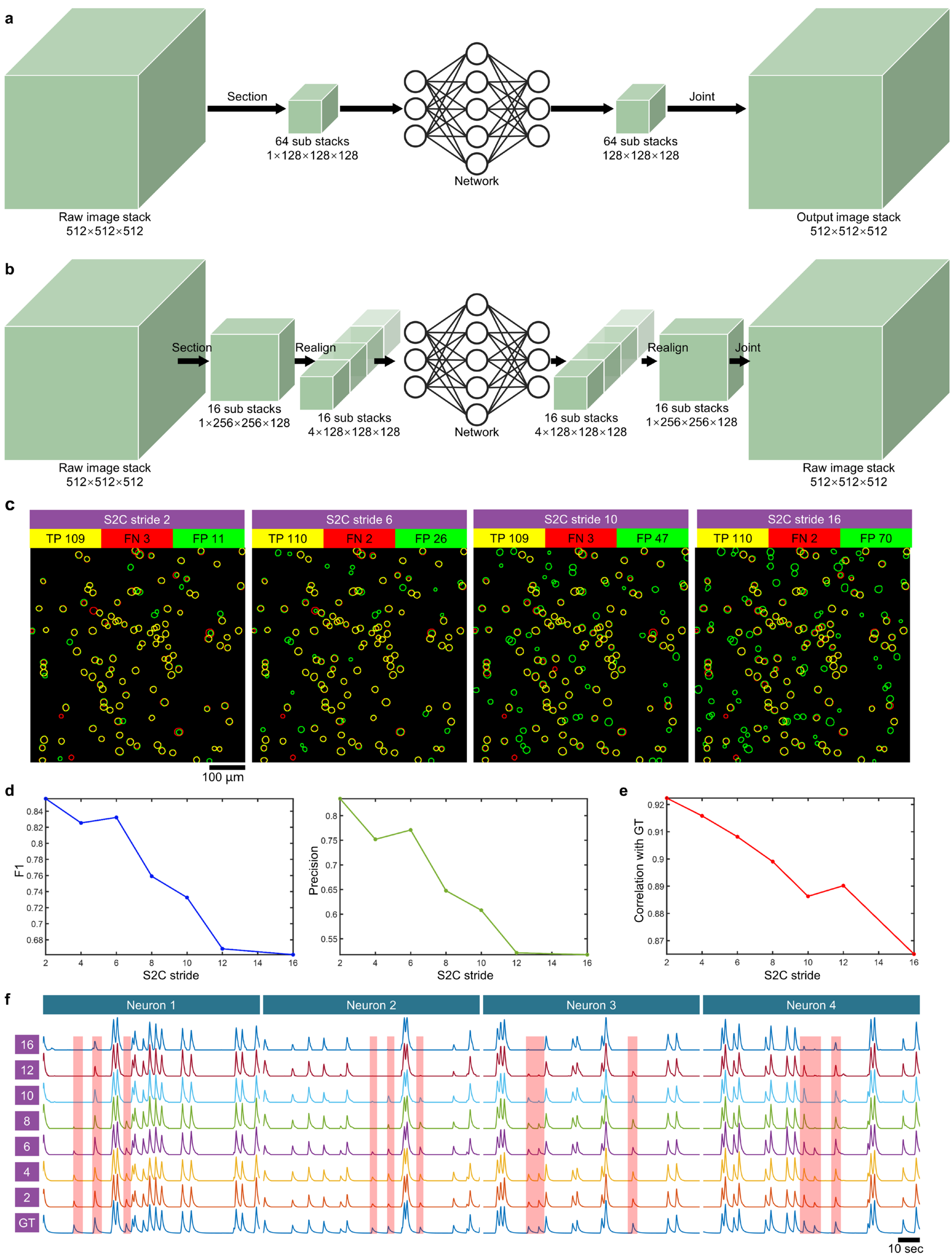

a
Section
Joint
Raw image stack
512×512×512
64 sub stacks
1×128×128×128
Network
64 sub stacks
128×128×128
Output image stack
512×512×512
b
Section
Realign
Realign
Joint
Raw image stack
512×512×512
16 sub stacks
1×256×256×128
16 sub stacks
4×128×128×128
Network
16 sub stacks
4×128×128×128
16 sub stacks
1×256×256×128
Raw image stack
512×512×512
c
S2C stride 2
TP 109
FN 3
FP 11
S2C stride 6
TP 110
FN 2
FP 26
S2C stride 10
TP 109
FN 3
FP 47
S2C stride 16
TP 110
FN 2
FP 70
100 μm
d
F1
S2C stride
Precision
S2C stride
e
Correlation with GT
S2C stride
f
Neuron 1
Neuron 2
Neuron 3
Neuron 4
16
12
10
8
6
4
2
GT
10 sec

Supplementary Figure 3

Evaluation of DeepWonder with different strides of S2C operation.

a. A test RB-Net module in DeepWonder without the spatial to channel (S2C) operation, where an input raw image stack of size 512×512×512 needs to be split into 64 subimage stacks with size 128×128×128. The core processing network thus has an input size of 1x128x128x128.

b. A test RB-Net module in DeepWonder with the S2C operation, where an input raw image stack of size 512×512×512 needs to be split into 16 subimage stacks of size 256×256×128. The core processing network thus has an input size of 4x128x128x128, but it has almost the same runtime compared to the core processing network in **a**. Thus, the processing time of RB-Net module in **b** is 4 times shorter than that in **a**.

c. The footprints of neuron segmentation change with S2C stride size. The stride size l determines realigning spatial $l \times l$ pixels on l^2 channels at the input.

d. Cell-level neuron segmentation statistics changes with S2C stride size (F score and precision score are calculated).

e. The correlation scores between DeepWonder output and corresponding ground truth data change with different S2C strides.

f. Exemplary 4 DeepWonder output traces change with different S2C strides. Red shaded area labels errors.

Scale bar: 100 μm in **c** and 10 seconds in **f**.

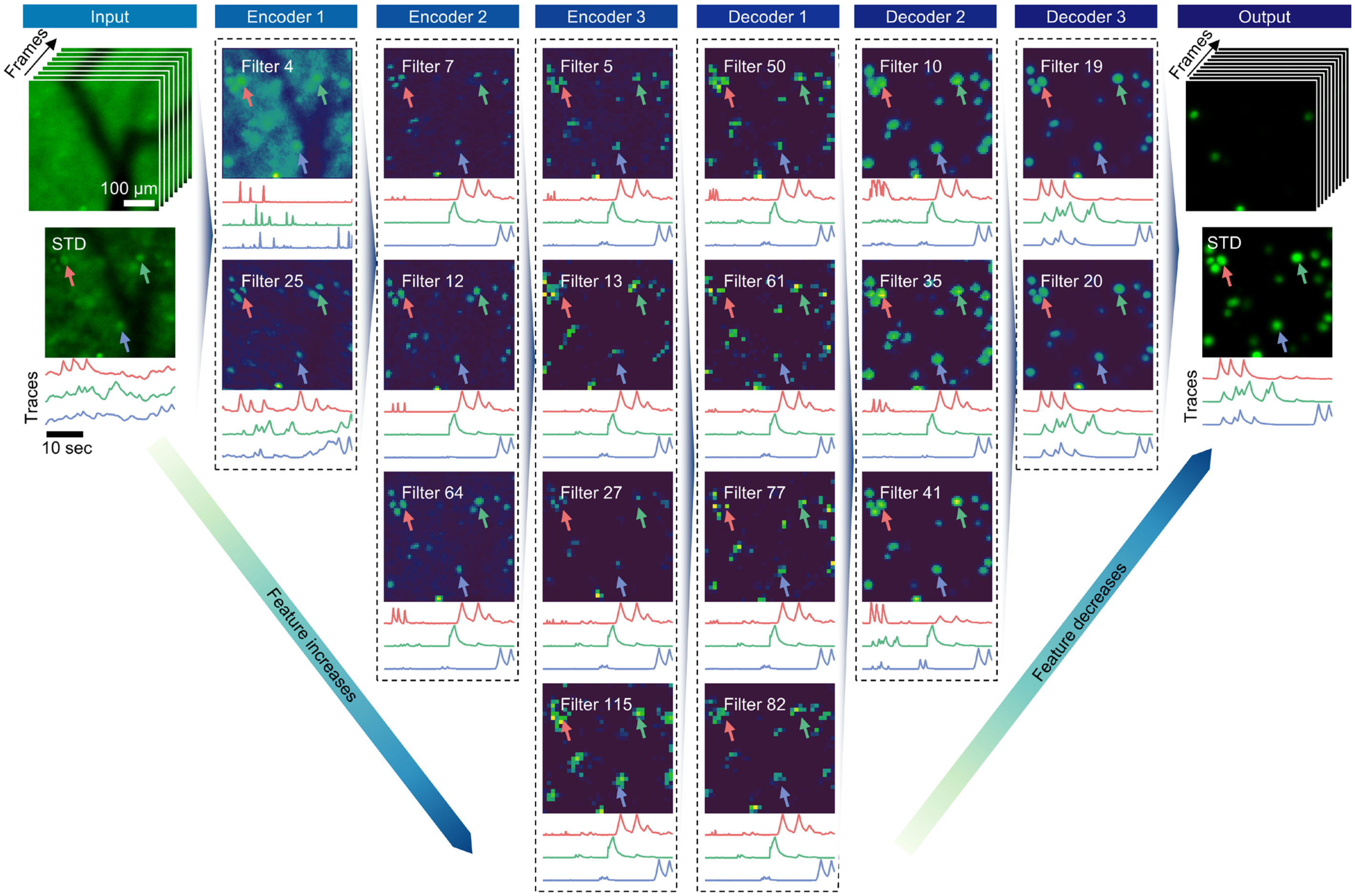

Supplementary Figure 4

Visualize the removing background process of RB-Net in DeepWonder.

The RB-Net is composed of three encoders (Encoder1, Encoder2, Encoder3) and three decoders (Decoder1, Decoder2, Decoder3) to implement background removal. We visualize the output features of three encoders and three decoders in this figure. The figure shows variance projection for feature visualization[1]. It can be noticed that as the data flows through the RB-Net, the neurons are highlighted while the background is removed. We selected three typical neurons (marked with red, green, and blue arrows) and displayed their time traces under the variance projection. With the removal of the background, the calcium signal traces become accurate and the background contamination signal is reduced.

Scale bar: 100 μm and 10 seconds.

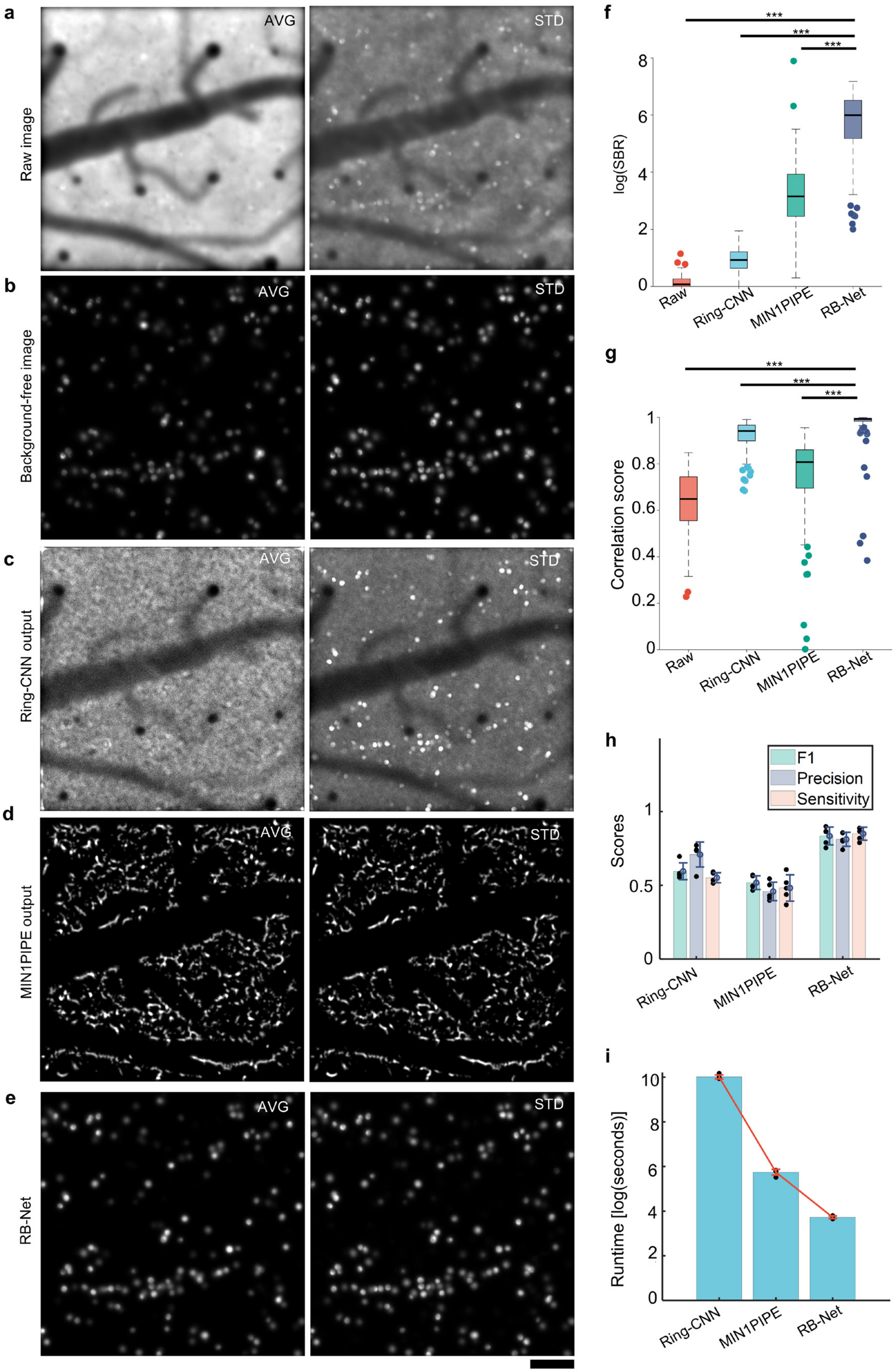

Supplementary Figure 5

RB-Net in DeepWonder outperforms both ring-CNN and MIN1PIPE in removing backgrounds.

a. Average (AVG, left) and standard deviation (STD, right) images of a simulated movie (10Hz, 1000 frames) by NAOMi1p with 600 x 600 μm FOV and 0.8 μm pixel size.

b. Average and standard deviation images of the background-free movie as ground truth, corresponding to **a**.

c-e. Average and standard deviation images of processed movies by ring-CNN, MIN1PIPE, and our RB-Net approach in DeepWonder, respectively.

f. Logarithm of signal-to-background ratio (SBR) of neurons in raw movie, ring-CNN movie, MIN1PIPE movie, and RB-Net movie. ***$P = 3.64\times10^{-43}$ between RB-Net and raw movie, ***$P = 3.64\times10^{-43}$ between RB-Net and ring-CNN movie, ***$P = 1.27\times10^{-28}$ between RB-Net and MIN1PIPE movie, two-sided Wilcoxon signed-rank test, n = 127 neurons from a single recording. Central black mark: Median. Bottom and top edges: 25th and 75th percentiles. Whiskers extend to extreme points excluding outliers (1.5 times above or below the interquartile range).

g. Boxplot of correlations of neurons in raw movie, ring-CNN movie, MIN1PIPE movie, and RB-Net movie. ***$P = 3.17\times10^{-39}$ between RB-Net and raw movie, ***$P = 4.27\times10^{-28}$ between RB-Net and ring-CNN movie, ***$P = 2.98\times10^{-37}$ between RB-Net and MIN1PIPE movie, two-sided Wilcoxon signed-rank test, n = 127 neurons from a single recording. Box plot elements as in **f**.

h. Precision, sensitivity, and F1 scores of neuron segmentation after applying CaImAn-Batch on the de-background movies from ring-CNN, MIN1PIPE, and RB-Net, respectively. The CaImAn-Batch parameters are the same across comparisons. Statistical scores are shown in mean ± SD across n = 5 simulated recordings. Height of bars: Mean. Error bars: SD. Black dots: n = 5 simulated recordings.

i. Runtimes of ring-CNN, MIN1PIPE, and RB-Net methods. Runtimes show in logarithmic scale, and the runtimes for ring-CNN, MIN1PIPE, and RB-Net are 22832 ± 1764, 341 ± 18, and 41 ± 3 seconds (mean ± SD) in n = 5 simulated recordings, respectively. Bar plot elements as in **h**.

Scale bar: 100 μm.

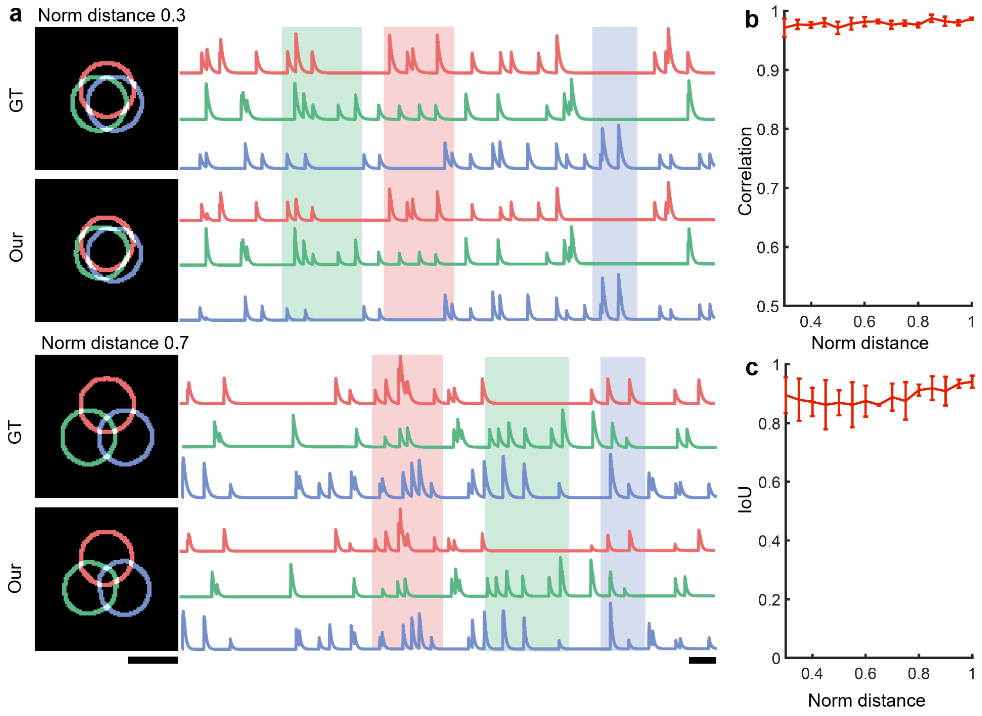

Supplementary Figure 6

Local NMF in segmentation stage effectively resolves overlapped neurons.

a. Segmentation results generated by NMF in segmentation module of DeepWonder, compared with ground truth. We simulate three Gaussian-shaped neurons with the same diameters. Top two rows: The footprints of three neurons with a center distance of 0.3 neuron diameter and the calcium signal traces extracted by NMF. Bottom two rows: The results of three neurons with a center distance of 0.7 neuron diameter. All the footprints and calcium transients match well with the ground truth. The neuronal distance is calculated by the distance between the center of mass of two neurons. The normalized distance (norm distance) is defined by the ratio of the neuronal distance over the neuron diameter.

b. The correlation scores of calcium signals change with increasing neuronal distance.

c. The Intersect over Union (IoU) of footprints in the presence of increasing neuron center distance.

Statistical scores are shown in mean ± SD across n = 3 simulated recordings.

Scale bar: 10 μm and 10 seconds.

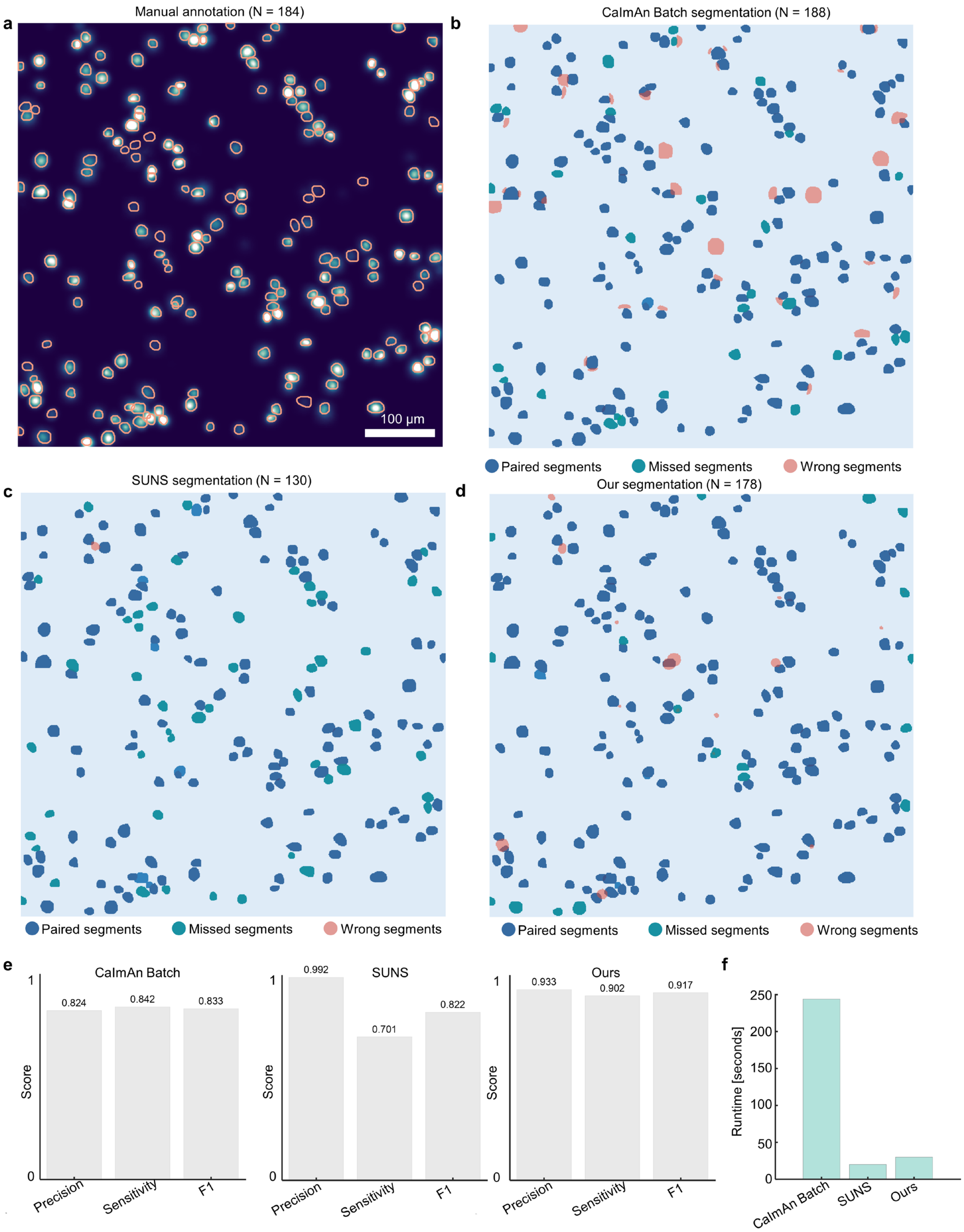

Supplementary Figure 7

NS-Net in DeepWonder outperforms both CaImAn Batch and SUNS in background-removed neuronal segmentation.

a. Ground truth segmentation of NAOMi1p data.

b-d. Results of CaImAn Batch, SUNS, and our segmentation method (NS-Net), respectively. Blue masks represent corrected segments, green masks represented missed segments by current methods, and pink masks represent wrong segments by current methods.

e. Precision, sensitivity, and F1 scores of CaImAn Batch, SUNS, and our method in segmentation.

f. Runtimes of CaImAn Batch, SUNS, and our method.

Scale bar: 100 μm.

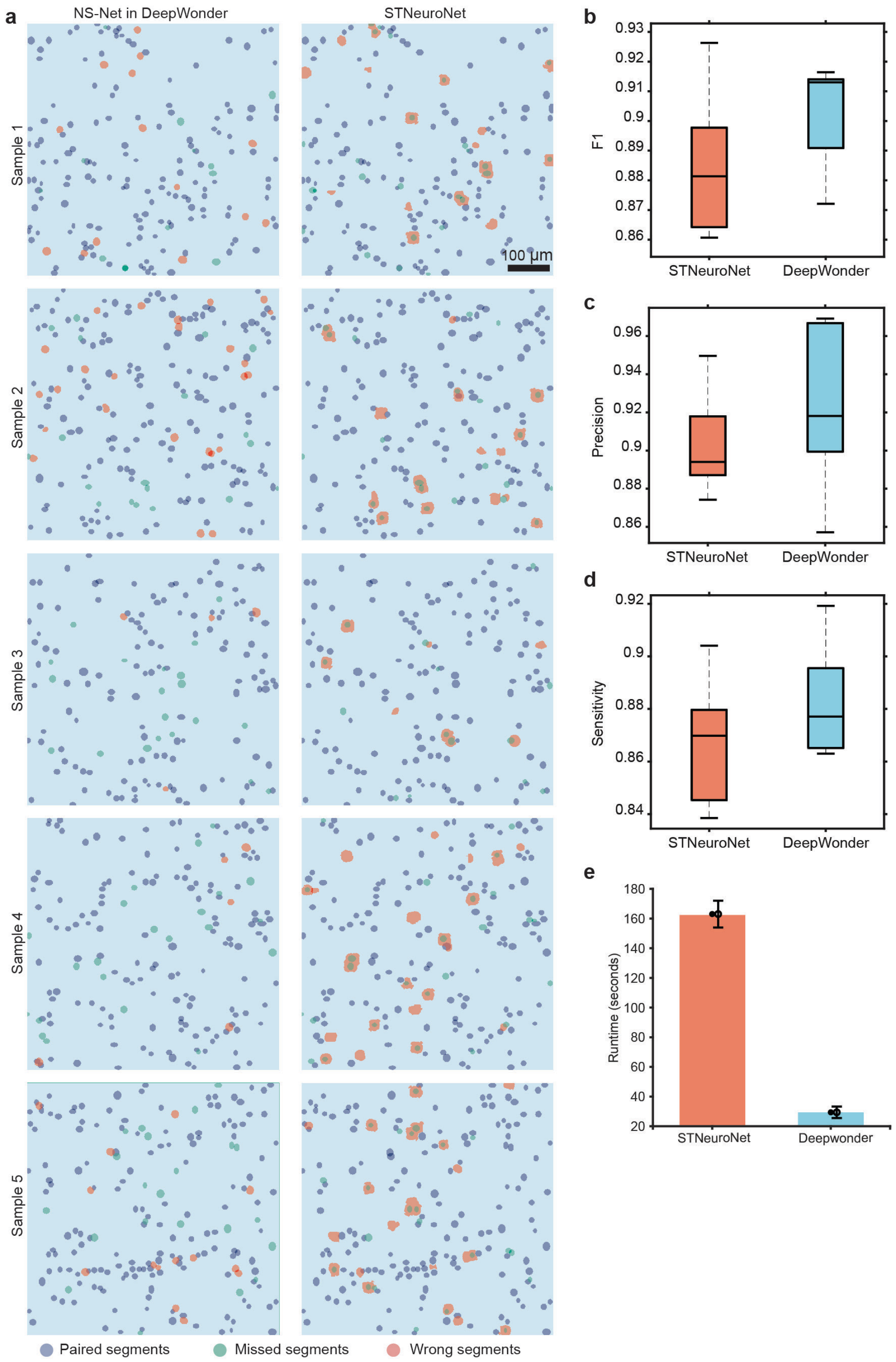

Supplementary Figure 8

Comparison of NS-Net in DeepWonder with the segmentation module in STNeuroNet.

a. Segmentation results by NS-Net in DeepWonder (the first column) and by STNeuroNet[2] (the second column). Blue masks represent corrected segments, green masks represent missed segments by current methods, and pink masks represent false segments by the current method.

b. F1 scores of NS-Net in DeepWonder (blue) and by STNeuroNet (red) in segmentation. Mean ± SD of F1 scores are 0.90 ± 0.02 for NS-Net in DeepWonder, and 0.88 ± 0.03 for STNeuroNet (n = 5 simulated recordings). Central black mark: Median. Bottom and top edges: 25th and 75th percentiles. Whiskers extend to extreme points excluding outliers (1.5 times above or below the interquartile range).

c. Precision scores of NS-Net in DeepWonder (blue) and by STNeuroNet (red) in segmentation. Mean ± SD of precision scores are 0.92 ± 0.05 for NS-Net in DeepWonder, and 0.90 ± 0.03 for STNeuroNet (n = 5 simulated recordings). Box plot elements as in **b**.

d. Sensitivity scores of NS-Net in DeepWonder (blue) and by STNeuroNet (red) in segmentation. Mean ± SD of sensitivity scores are 0.88 ± 0.02 for NS-Net in DeepWonder, and 0.87 ± 0.03 for STNeuroNet (n = 5 simulated recordings). Box plot elements as in **b**.

e. Runtimes of NS-Net in DeepWonder (blue) and STNeuroNet (red). Mean ± SD of runtime are 29.4 ± 3.85 seconds for NS-Net in DeepWonder, and 163 ± 9.06 seconds for STNeuroNet (n = 5 simulated recordings, sample size of 750 x 750 pixels and 1000 frames). Height of bars: Mean. Error bars: SD. Black dots: n = 5 simulated recordings.

Scale bar: 100 μm.

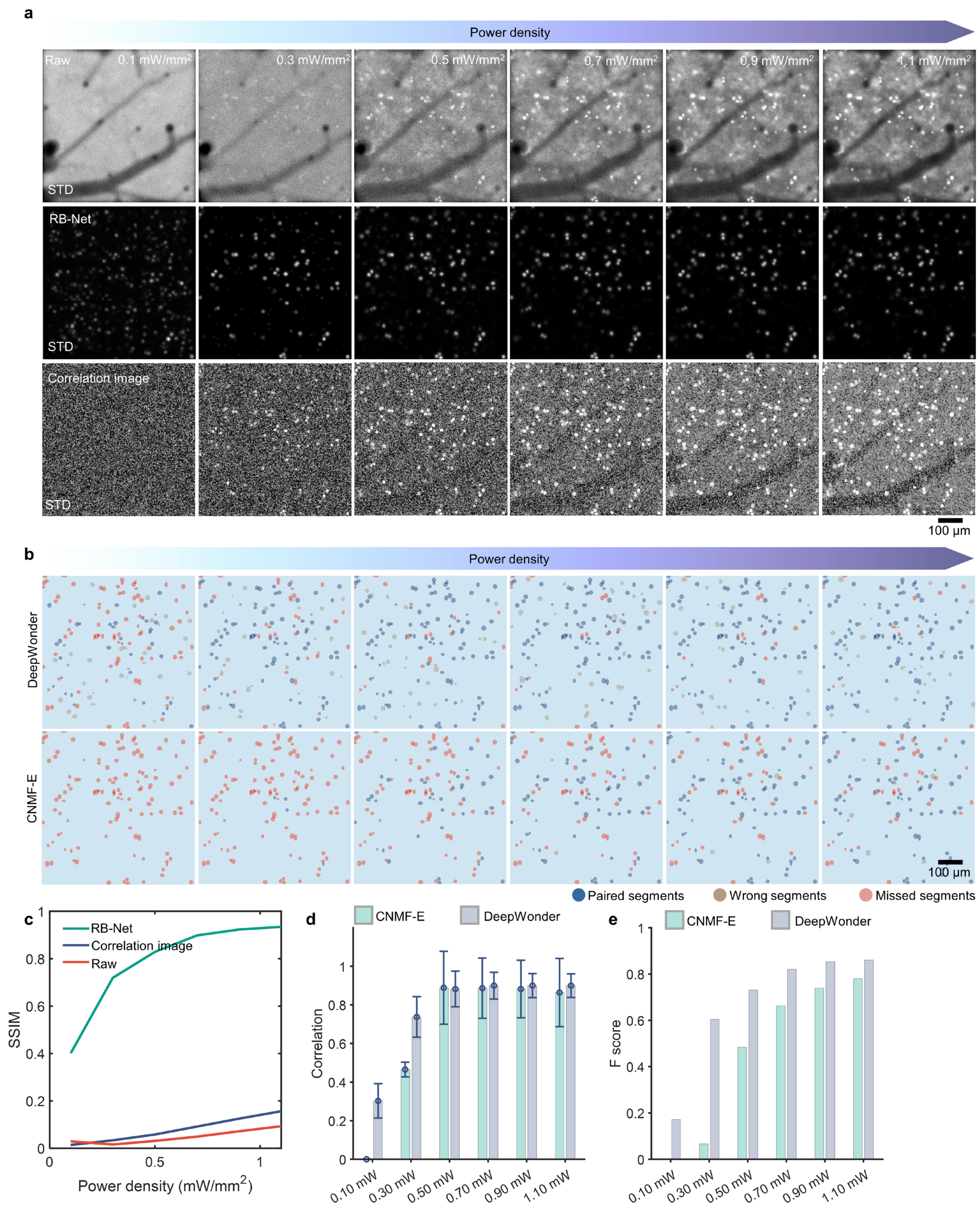

Supplementary Figure 9

DeepWonder functions robustly across different noise scales.

a. First row, standard deviation (STD) of 1000 simulated frames at 10 Hz by NAOMi1p, from 0.1 to 1.1 mW/mm^2 illumination power density. Second row, STD of the de-background images by RB-Net. Third row, correlation image of the virtual captures by CNMF-E.

b. First row, segmentation results of **a** by DeepWonder, from 0.1 to 1.1 mW/mm^2 illumination power. Blue masks represent corrected segments compared to ground truth, red masks represented missed segments, and brown masks represent wrong segments. Second row, segmentation results by CNMF-E.

c. Red curve: structure similarity index (SSIM) of STD of raw captures from 0.1 to 1.1 mW/mm^2, with STD of raw captures at 1.5 mW/mm^2 as the reference. Blue curve: the SSIM across the same power density range but by correlation image from CNMF-E. Green curve: the SSIM across the same power density range but by STD of RB-Net de-background recordings.

d. Correlation scores of CNMF-E (green) and DeepWonder (blue) change from 0.1 to 1.1 mW/mm^2 (mean ± SD). Height of bars: Mean. Error bars: SD. n = 6 simulated recordings.

e. Segmentation accuracy (F score) of CNMF-E (green) and DeepWonder (blue) changes from 0.1 to 1.1 mW/mm^2.

Scale bar: 100 μm.

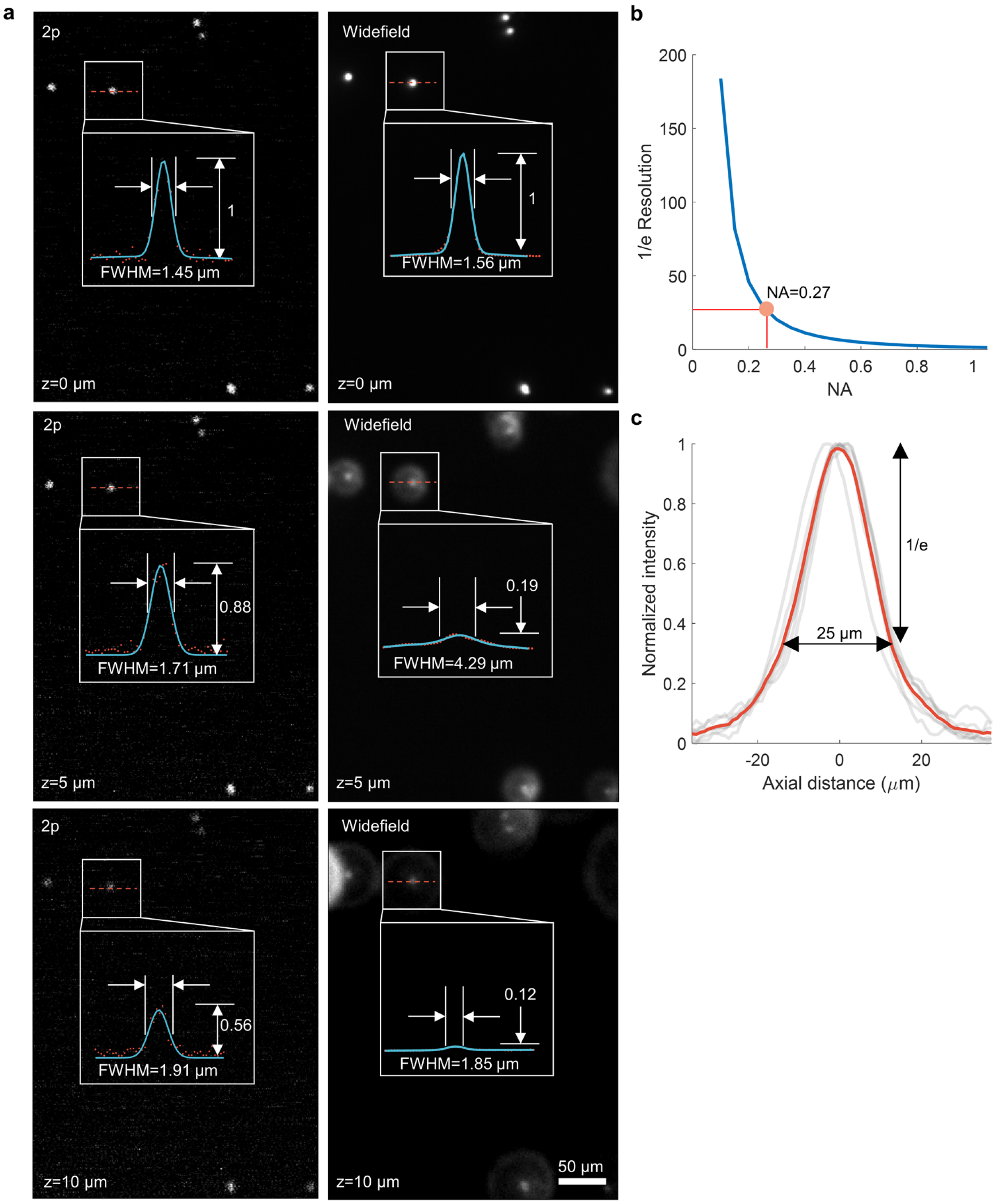

Supplementary Figure 10

Calibration of the joint 2p-widefield detection system.

a. First row, 2p (left) and widefield (right) imaging of 1-μm diameter fluorescent beads. The white box isolates one of fluorescent beads and the intensity profile across the red dashed line is indicated in the zoom-in panel. Red dots indicate raw data and blue curves indicated Gaussian fitted data with MATLAB command *fit*. The full width at half maximum (FWHM) of each profile is marked on the bottom side, and relative peak intensity is marked on the right side. The second row and the third row are similar to the first row but defocus the sample by z = 5 μm and z = 10 μm, respectively. As defocus increases, the low-NA 2p PSF shows consistent tight focus and high intensity, while the PSF by the widefield spread fast. Note when the defocus reaches z = 10 μm, the defocus ring of beads in widefield becomes too large, and the FWHM thus is only measured at the central peak and has a smaller value.

b. Axial resolution in 1/e changes with excitation NA in simulation[3]. With calibration, we find that our system has a valid NA of 0.27.

c. Axial PSF calibration of the low-NA microscope (n = 5 beads across the FOV. The red line shows the average curve).

Scale bar: 50 μm.

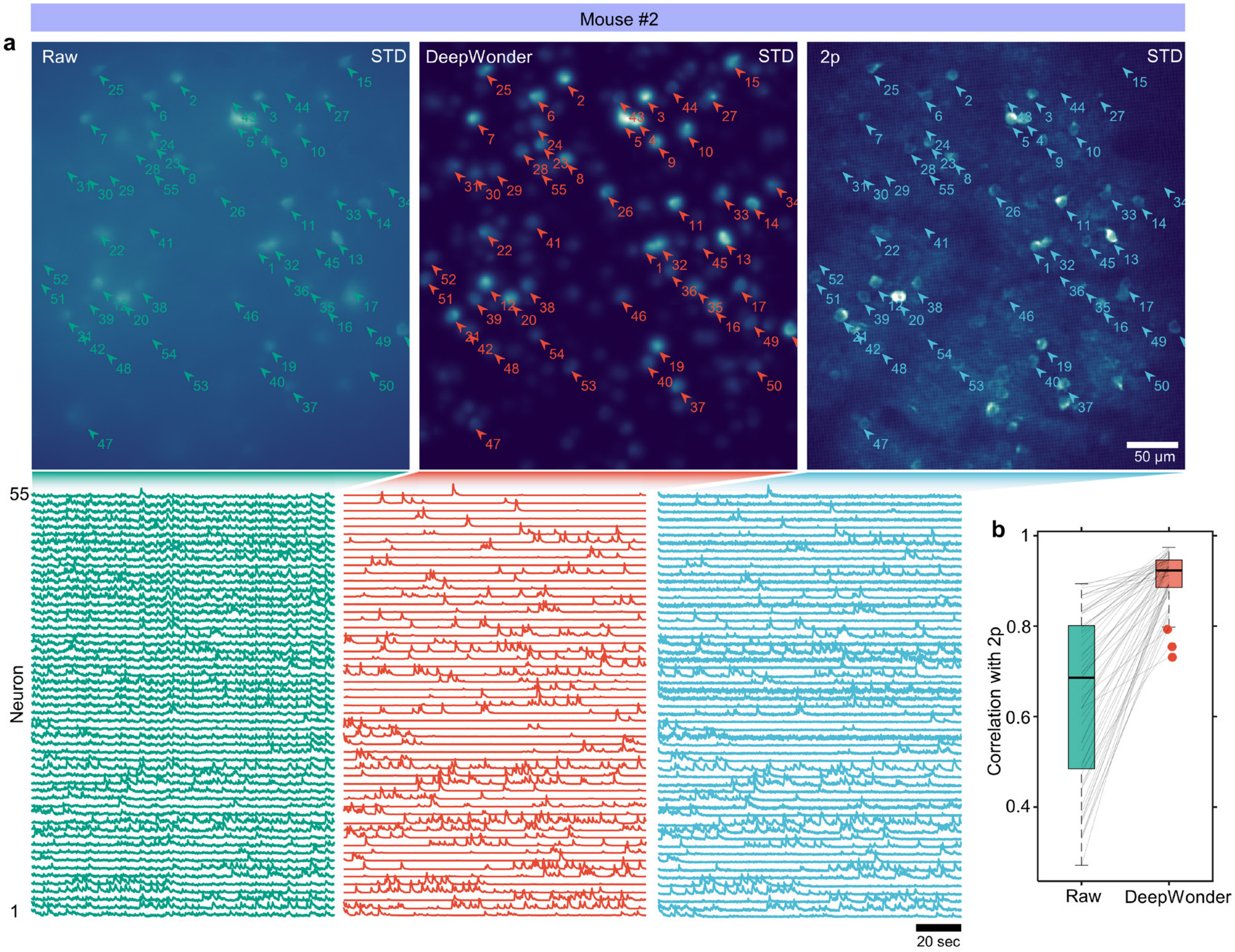

Supplementary Figure 11

DeepWonder retrieves accurate calcium inference with joint widefield-2p detection (mouse #2).

a. Standard deviation (STD) of raw widefield movie (left), RB-Net in DeepWonder processed widefield movie (middle), and two-photon movie (right). Arrows mark neurons and the corresponding temporal activities plotted in the bottom side.

b. Temporal correlation scores of 55 picked neurons by RB-Net output movie (red) and raw movie (green) with 2p ground truth. Central black mark: Median. Bottom and top edges: 25th and 75th percentiles. Whiskers extend to extreme points excluding outliers (1.5 times above or below the interquartile range).

Scale bar: 50 μm and 20 seconds.

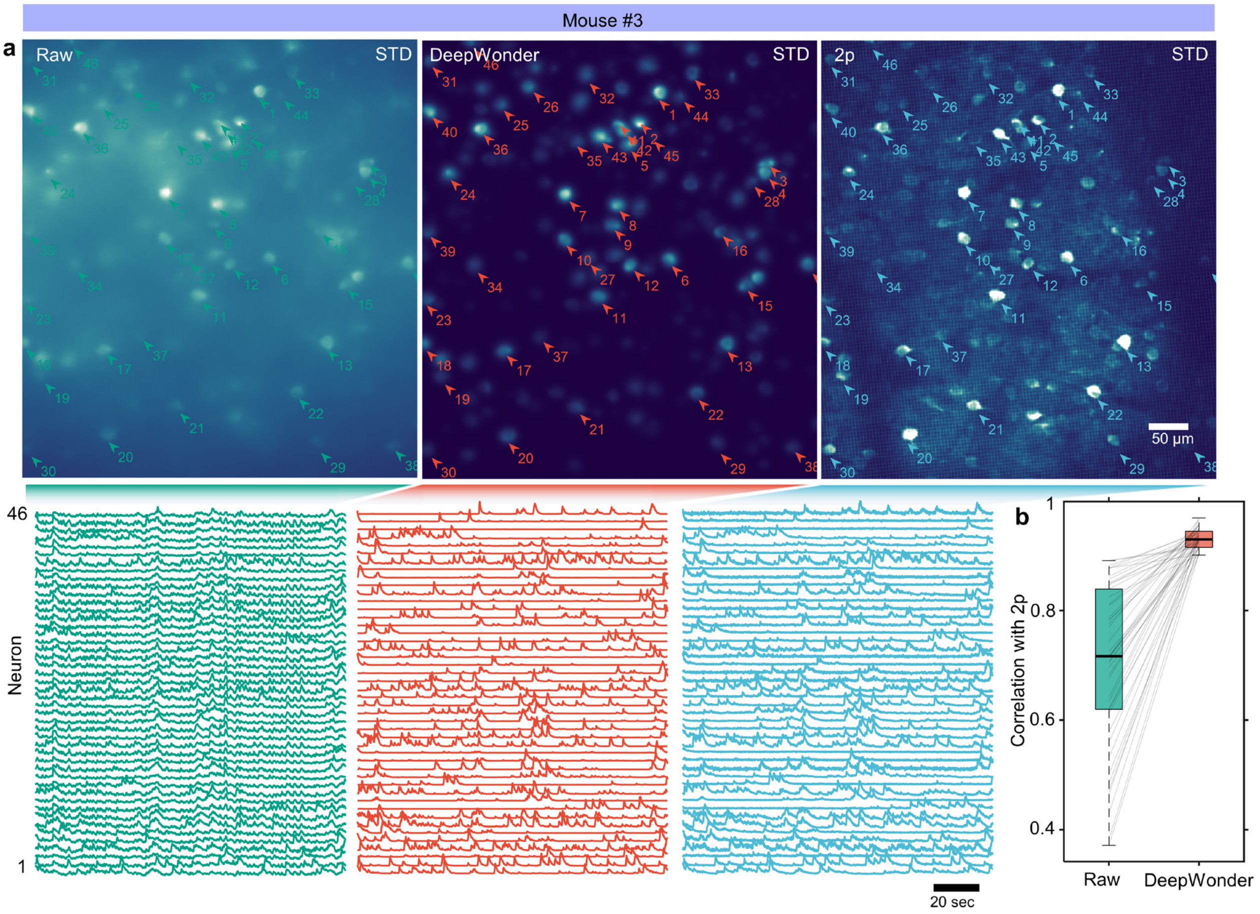

Supplementary Figure 12

DeepWonder retrieves accurate calcium inference with joint widefield-2p detection (mouse #3).

a. Standard deviation (STD) of raw widefield (left), RB-Net in DeepWonder processed widefield movie (middle), and two-photon movie (right). Arrows mark neurons and the corresponding temporal activities plotted in the bottom side.

b. Temporal correlation scores of 46 picked neurons by RB-Net output movie (red) and raw movie (green) with 2p ground truth. Central black mark: Median. Bottom and top edges: 25th and 75th percentiles. Whiskers extend to extreme points excluding outliers (1.5 times above or below the interquartile range).

Scale bar: 50 μm and 20 seconds.

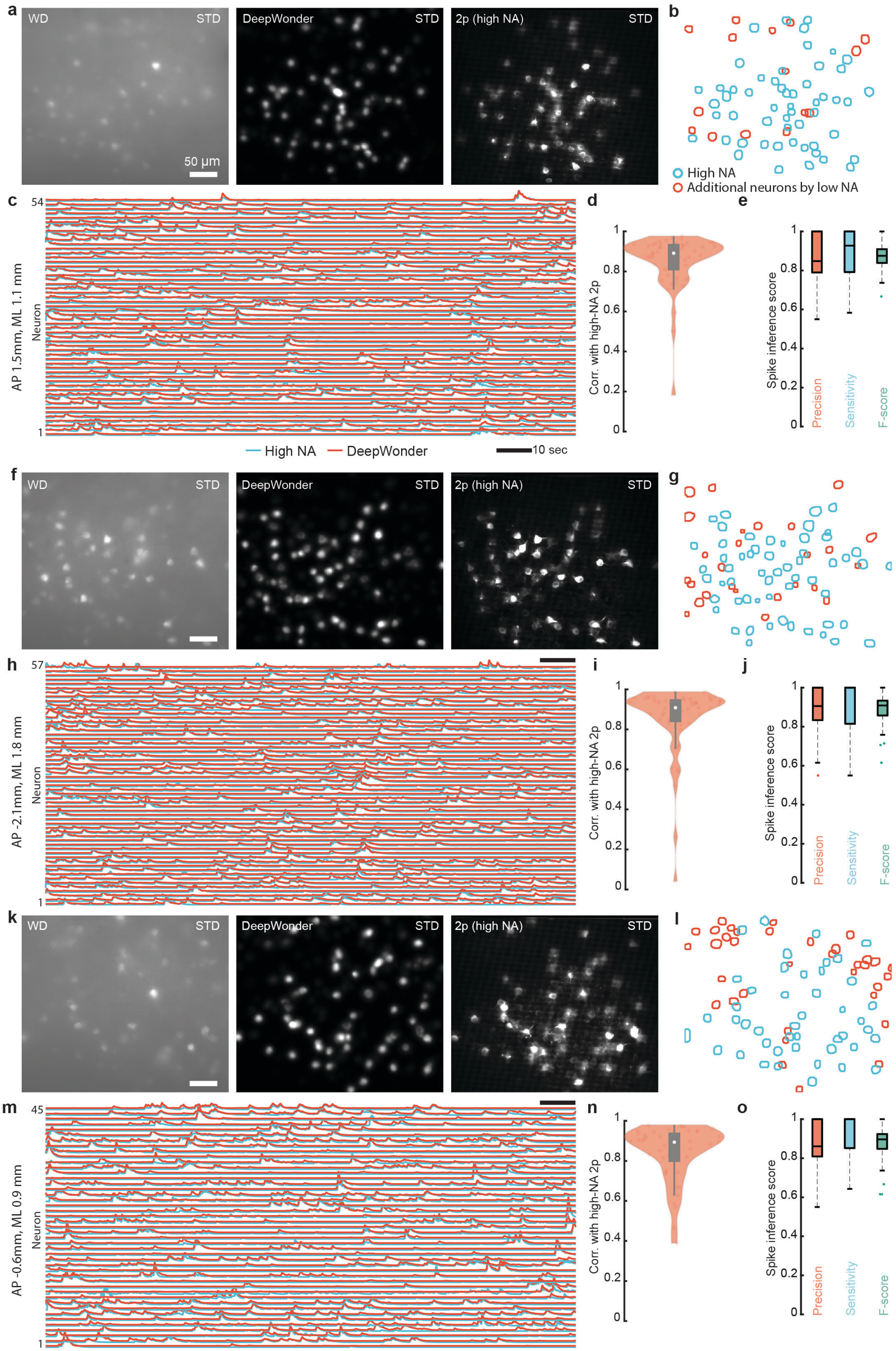

a
WD
STD
50 μm
DeepWonder
STD
2p (high NA)
STD
b
High NA
Additional neurons by low NA
c
54
1
Neuron
AP 1.5mm, ML 1.1 mm
High NA
DeepWonder
10 sec
d
Corr. with high-NA 2p
e
Spike inference score
Precision
Sensitivity
F-score
f
WD
STD
DeepWonder
STD
2p (high NA)
STD
g
h
57
1
Neuron
AP -2.1mm, ML 1.8 mm
i
Corr. with high-NA 2p
j
Spike inference score
Precision
Sensitivity
F-score
k
WD
STD
DeepWonder
STD
2p (high NA)
STD
l
m
45
1
Neuron
AP -0.6mm, ML 0.9 mm
n
Corr. with high-NA 2p
o
Spike inference score
Precision
Sensitivity
F-score

Supplementary Figure 13

Validation of DeepWonder functional signals with high-NA ground truth.

a. Standard deviation (STD) images of the raw movie by widefield (WD, left), DeepWonder processed movie (middle), and high-NA 2p recordings (right). Some neurons that are clear in the DeepWonder processed movie are invisible in high-NA 2p recordings.

b. Neuron segmentation by DeepWonder processed movie compared to high-NA 2p recordings. Shallow blue circles indicate neurons that are found in both DeepWonder and high-NA data, and red circles indicate neurons that are only found in DeepWonder data.

c. Neuronal calcium activities from DeepWonder data (red) and corresponding high-NA functional ground truth (blue). Traces by DeepWonder are offset vertically for clarity.

d. Distributions of temporal correlation between DeepWonder extracted neuronal traces and corresponding high-NA traces across 54 paired neurons. White circle: median. Vertical grey bar: interquartile range. Transparent disks: data points. Transparent violin-shaped areas: kernel density estimate of data distribution.

e. Spike inference scores (precision, sensitivity, and F-score) achieved by OASIS[4] on DeepWonder traces compared to high-NA functional ground truth, across 54 paired neurons in a capture of 4500 frames. Central black mark: Median. Bottom and top edges: 25th and 75th percentiles. Whiskers extend to extreme points excluding outliers (1.5 times above or below the interquartile range).

f-**j** and **k**-**o** are the same as **a**-**e** but at different anterior-posterior (AP) and medial-later (ML) positions, which are labeled in the left side. Violin plot elements as in **d** and box plot elements as in **e**. n = 57 neurons in **f**-**j** and 45 neurons in **k**-**o**.

Scale bars: 50 μm in **a**, **f**, and **k**, and 10 seconds in **c**, **h**, **m.**

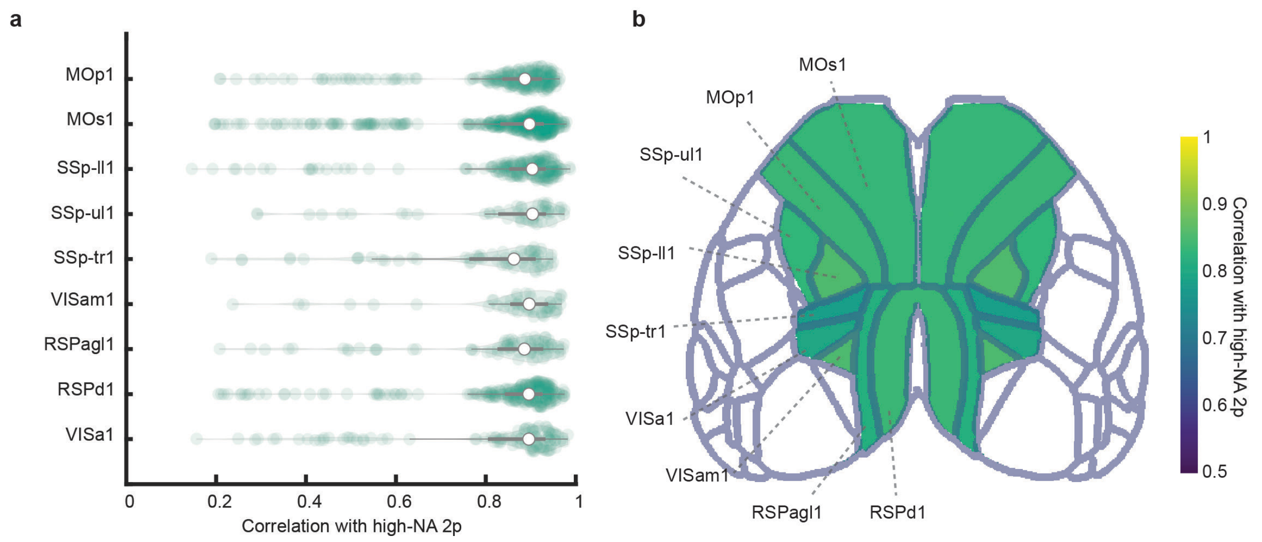

Supplementary Figure 14

Validation of DeepWonder on brain wide with hybrid widefield and high-NA recordings.

a. Distribution of temporal correlation scores between DeepWonder-extracted traces and corresponding high-NA 2p functional ground truth in different brain regions (1545 neurons from 9 cortical regions, n = 20 recordings from 3 mice). White circle: Median. Thick grey vertical line: Interquartile range. Thin vertical lines: Upper and lower proximal values. Transparent disks: Data points. Transparent violin-shaped areas: Kernel density estimate of data distribution.

b. Spatial distribution of neuron correlation scores achieved by DeepWonder on experimental high-NA functional verification datasets overlaid with Allen CCF atlas. Accessed cortical regions include Primary motor area Layer 1 (MOp1), Secondary motor area layer 1 (MOs1), Primary somatosensory area lower limb layer 1 (SSp-ll1), Primary somatosensory area upper limb layer 1 (SSp-ul1), Primary somatosensory area trunk layer 1 (SSp-tr1), Anteromedial visual area layer 1 (VISam1), Retrosplenial area lateral agranular part layer 1 (RSPagl1), Retrosplenial area dorsal part layer 1 (RSPd1), and Anterior area layer 1 (VISa1). Brain-wide correlation score reached 0.83 ± 0.02 (mean ± SD, n = 9 cortical regions).

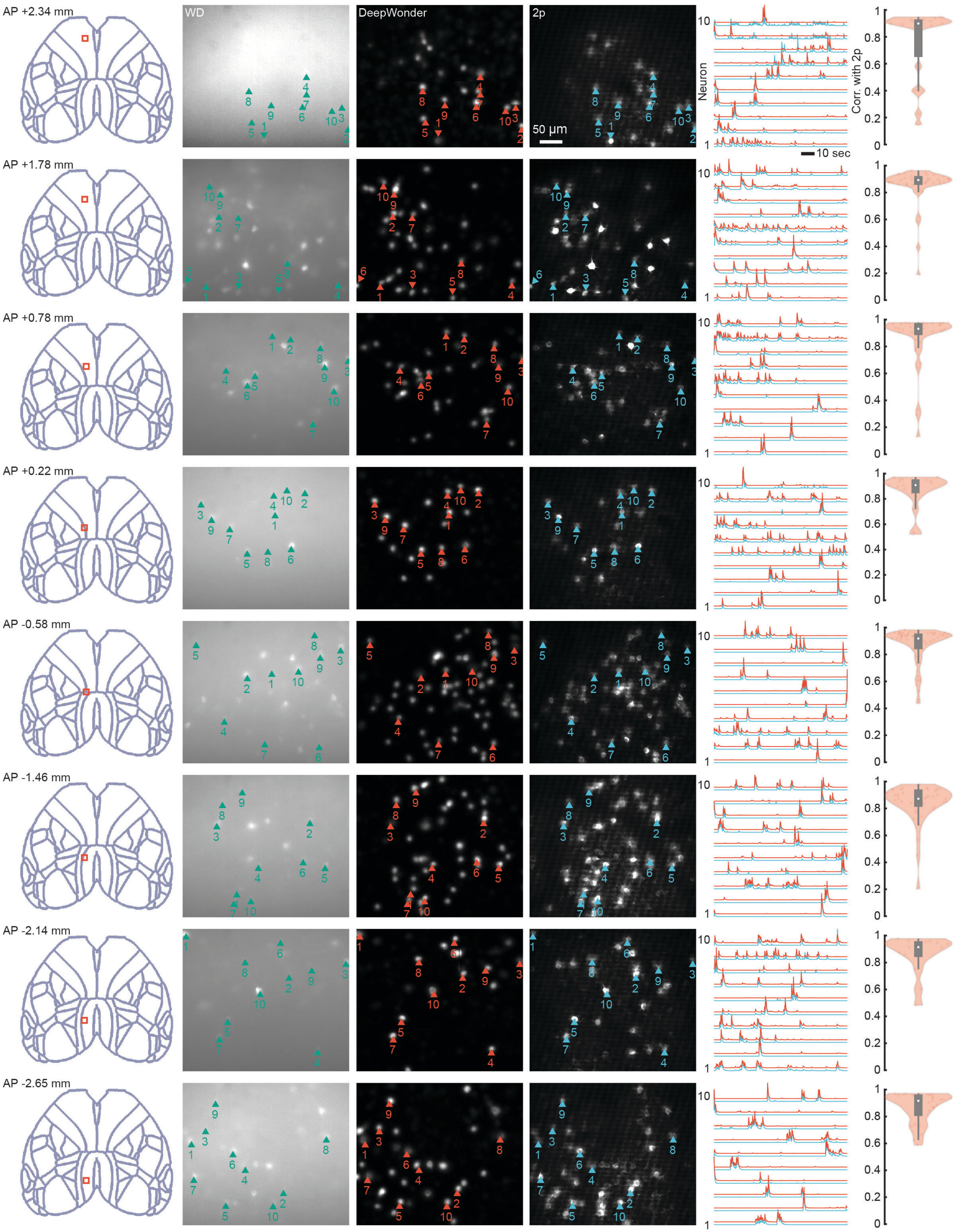

AP +2.34 mm
AP +1.78 mm
AP +0.78 mm
AP +0.22 mm
AP -0.58 mm
AP -1.46 mm
AP -2.14 mm
AP -2.65 mm
WD
DeepWonder
2p
50 μm
Neuron
10 sec
Corr. with 2p

Supplementary Figure 15

Validation of DeepWonder on different anterior-posterior positions in mouse cortex.

From top to bottom: 8 different anterior-posterior (AP) positions are accessed by hybrid widefield-2p detection system and processed by DeepWonder. The first column labels the location of imaging FOV by red boxes in Allen CCF atlas[5] (blue contour). The bregma point is the zero point, where positions on the anterior side are positive and positions on the posterior side are negative. AP positions are labeled in the top left corner for each capture. The second to the fourth columns show the standard deviation (STD) image across temporal frames of raw widefield movie (WD), DeepWonder processed movie, and 2p movie, respectively. Randomly selected 10 neurons are labeled by arrows and numbers in each panel, where green for raw movie, red for DeepWonder movie, and blue for 2p movie. The fifth column shows neuronal activity traces corresponding to arrows in STD panels as used for performance quantifications. Red traces for DeepWonder movie and blue traces for 2p movie, and DeepWonder traces are offset vertically for clarity. The sixth column shows distributions of temporal correlation between DeepWonder-extracted traces and corresponding 2p ground truth traces. White circle: Median. Thick grey vertical line: Interquartile range. Thin vertical lines: Upper and lower proximal values. Transparent disks: Data points. Transparent violin-shaped areas: Kernel density estimate of data distribution. n = 19, 44, 37, 19, 66, 40, 15, 31 neurons were evaluated for temporal correlation assessment over the 8 AP positions (from top to bottom), respectively.

Scale bar: 50 μm and 10 seconds.

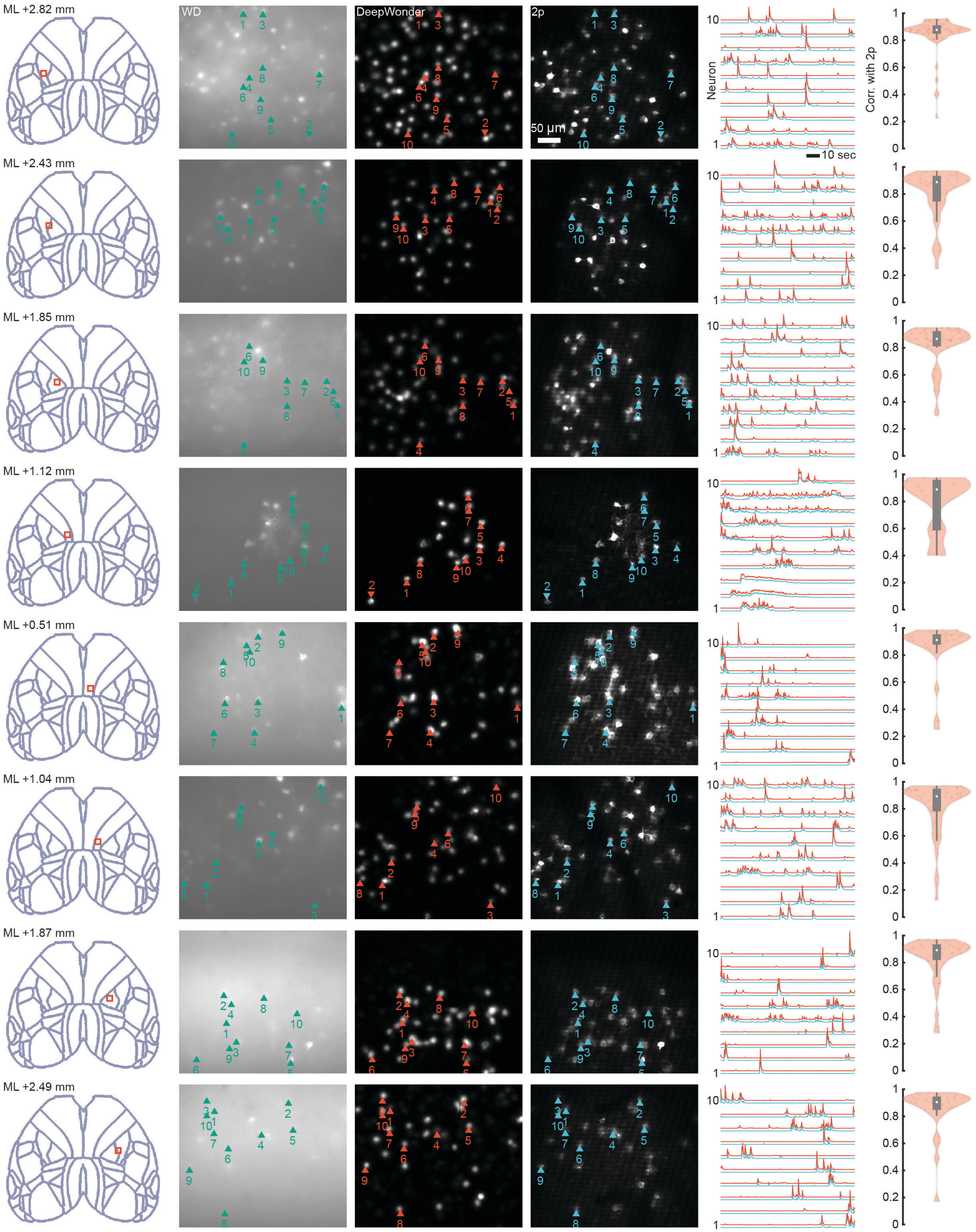
ML +2.82 mm
ML +2.43 mm
ML +1.85 mm
ML +1.12 mm
ML +0.51 mm
ML +1.04 mm
ML +1.87 mm
ML +2.49 mm
WD
DeepWonder
2p
50 μm
Neuron
10 sec
Corr. with 2p

Supplementary Figure 16

Validation of DeepWonder on different medial-lateral positions in mouse cortex.

From top to bottom: 8 different medial-lateral (ML) positions are accessed by hybrid widefield-2p detection system and processed by DeepWonder. The first column labels the location of imaging FOV by red boxes in Allen CCF atlas (blue contour). The bregma point is the zero point, where positions on the lateral side are positive. ML positions are labeled in the top left corner for each capture. The second to the fourth columns show the standard deviation (STD) image across temporal frames of raw widefield movie, DeepWonder processed movie, and 2p movie, respectively. Randomly selected 10 neurons are labeled by arrows and numbers in each panel, where green for raw movie, red for DeepWonder movie, and blue for 2p movie. The fifth column shows neuronal activity traces corresponding to arrows in STD panels as used for performance quantifications. Red traces for DeepWonder movie and blue traces for 2p movie, and DeepWonder traces are offset vertically for clarity. The sixth column shows distributions of temporal correlation between DeepWonder-extracted traces and corresponding 2p ground truth traces. White circle: Median. Thick grey vertical line: Interquartile range. Thin vertical lines: Upper and lower proximal values. Transparent disks: Data points. Transparent violin-shaped areas: Kernel density estimate of data distribution. n = 71, 40, 59, 14, 30, 52, 45, 29 neurons were evaluated for temporal correlation assessment over the 8 ML positions (from top to bottom), respectively.

Scale bar: 50 μm and 10 seconds.

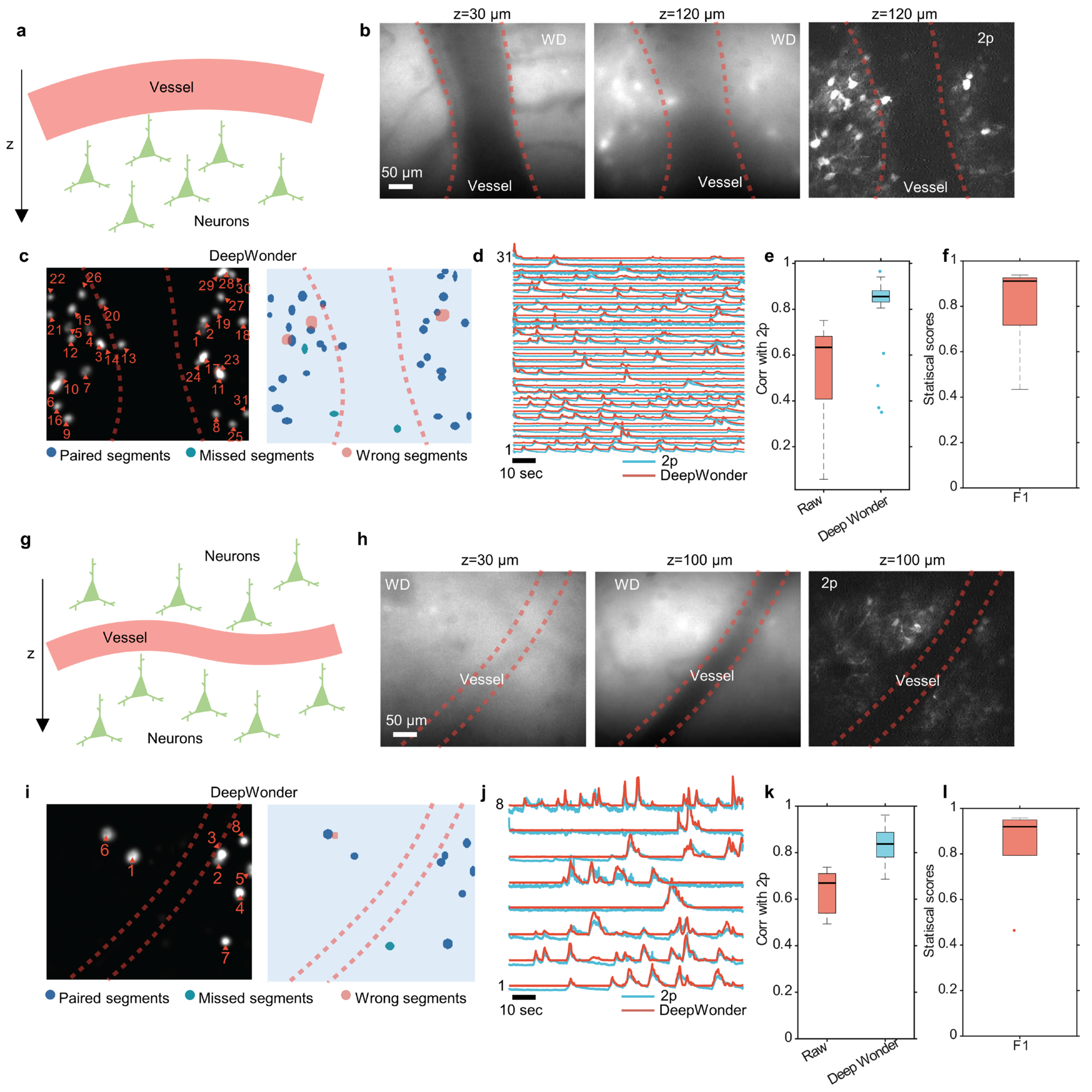

Supplementary Figure 17

Validation of DeepWonder encountering hemodynamic responses.

a. Schematics of an imaging condition where vessels are above target neuronal population.

b. Left to right, exemplary frames by widefield (WD) in z = 30 μm, WD frame in z = 120 μm, and corresponding 2p frame in z = 120 μm. Dashed red lines outline the boundary of vessels. Vessels are clear in z = 30 μm and neurons are clear in z = 120 μm.

c. Standard deviation (STD) across temporal frames of DeepWonder processed movie (left) and corresponding segmentation (right). Blue masks represent corrected segments compared to ground truth segments, green masks represent missed segments, and red masks represent wrong segments.

d. Neuronal activity traces corresponding to arrows in **c** are used for performance quantifications. Red traces for DeepWonder movie and blue traces for 2p movie, and DeepWonder traces are offset vertically for clarity.

e. Temporal correlations of detected neurons with 2p functional ground truth from DeepWonder movie (0.82 ± 0.15, mean ± SD, blue) and raw movie (0.55 ± 0.18, mean ± SD, red), over n = 198 neurons from 6 recordings across 3 mice. Central black mark: Median. Bottom and top edges: 25th and 75th percentiles. Whiskers extend to extreme points excluding outliers (1.5 times above or below the interquartile range).

f. F1 scores of segmentation by DeepWonder are 0.86 ± 0.15 (mean ± SD) from 6 recordings over 3 mice. Box plot elements as in **e**.

g. Schematics of an imaging condition where vessels and target neuronal population are in the same depth. Vessels are clear in z = 100 μm and neurons are clear in z = 100 μm.

h-**j** are the same as **b**-**d** but in new data (where vessels and target neuronal population are in the same depth, like in **g**).

k. Temporal correlations of detected neurons with 2p functional ground truth from DeepWonder movie (0.83 ± 0.09, mean ± SD, blue) and raw movie (0.64 ± 0.09, mean ± SD, red), over n = 121 neurons from 5 recordings across 3 mice. Box plot elements as in **e**.

l. F1 scores of segmentation by DeepWonder are 0.88 ± 0.15 (mean ± SD) from 5 recordings across 3 mice. Box plot elements as in **e**.

Scale bar: 50 μm and 10 seconds.

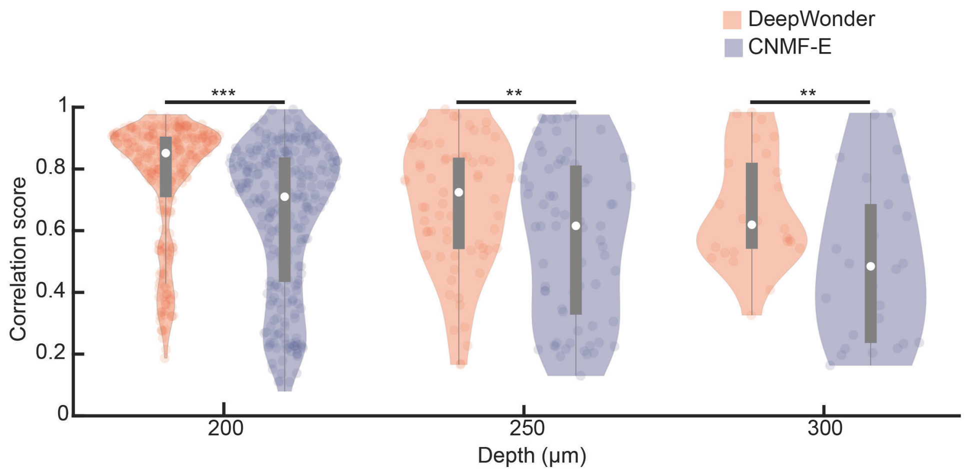

Supplementary Figure 18

Comparison of DeepWonder and CNMF-E in 200-300 μm deep mouse cortical imaging.

Distributions of temporal correlation scores of DeepWonder (red) and CNMF-E (purple) as a function of depths. Mean ± SD of correlation scores of DeepWonder are 0.77 ± 0.20, 0.68 ± 0.21, 0.67 ± 0.18 from 200 to 300 μm at a step of 50 μm, respectively. Mean ± SD of correlation scores of CNMF-E are 0.64 ± 0.24, 0.58 ± 0.26, 0.50 ± 0.26 from 200 to 300 μm at a step of 50 μm, respectively. n = 258, 69, 26 neurons at 200, 250, and 300 μm, respectively. ***$P = 4.06\times10^{-13}$, **$P = 0.03$, **$P = 0.01$ for depth 200, 250, and 300 μm, respectively, by two-sided Wilcoxon signed-rank test. The CNMF-E intakes paired DeepWonder and two-photon segmentation masks as initializations. Data from n = 11 recordings across 4 mice. White circle: Median. Thick grey vertical line: Interquartile range. Thin vertical lines: Upper and lower proximal values. Transparent disks: Data points. Transparent violin-shaped areas: Kernel density estimate of data distribution.

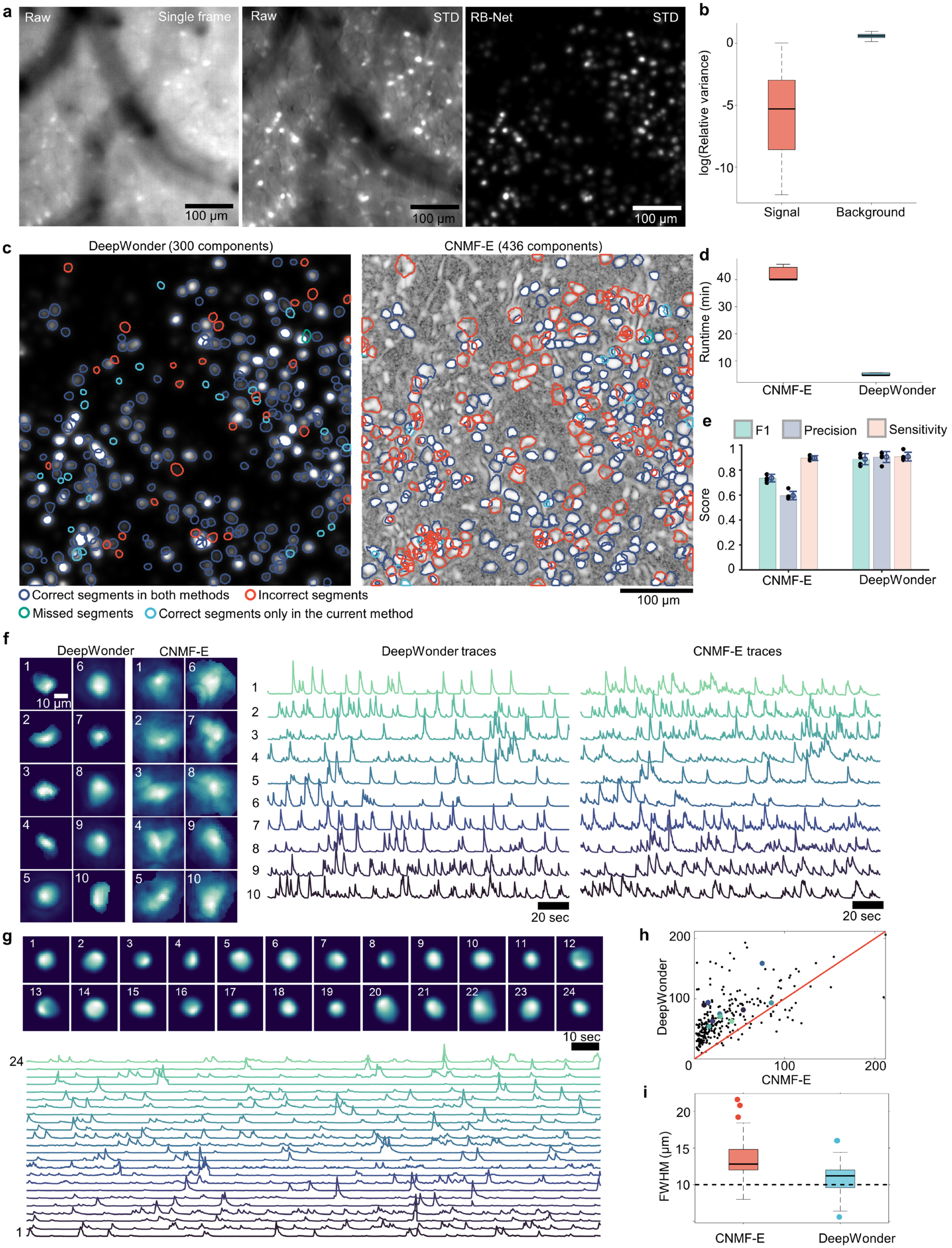
a
Raw
Single frame
100 μm
Raw
STD
100 μm
RB-Net
STD
100 μm
b
log(Relative variance)
0
-5
-10
Signal
Background
c
DeepWonder (300 components)
CNMF-E (436 components)
Correct segments in both methods
Incorrect segments
Missed segments
Correct segments only in the current method
100 μm
d
Runtime (min)
40
30
20
10
CNMF-E
DeepWonder
e
F1
Precision
Sensitivity
Score
1
0.8
0.6
0.4
0.2
0
CNMF-E
DeepWonder
f
DeepWonder
CNMF-E
10 μm
DeepWonder traces
CNMF-E traces
20 sec
20 sec
g
10 sec
24
1
h
DeepWonder
CNMF-E
200
100
0
100
200
i
FWHM (μm)
20
15
10
CNMF-E
DeepWonder

Supplementary Figure 19

Comparisons of DeepWonder and CNMF-E in RUSH dataset.

a. A single frame of raw recordings (left), standard deviation (STD) of raw recordings (middle), and STD of background removed images by proposed RB-Net (right).

b. Variance explanations by background (red) and neuronal signals (blue) over all pixels in logarithmic scale[6]. The variance of signals is calculated based on background removed images by RB-Net. This plot shows the background dominates the variance explanation in widefield microscope. Data from n = 329476 pixels in a single recording. Central black mark: Median. Bottom and top edges: 25th and 75th percentiles. Whiskers extend to extreme points excluding outliers (1.5 times above or below the interquartile range).

c. The contour plot of all neurons detected by DeepWonder (left) and CNMF-E (right) superimposed on the STD of background removed images and correlation image from CNMF-E, respectively. Compared to manual segmentation, deep blue circles mark correct segments in both methods, red circles mark incorrect segments in each method, green circles mark missed segments in each method, and shallow blue circles mark correct segments that are only in the current method.

d. Runtime comparisons of CNMF-E (red) and DeepWonder (blue). The raw movie has a size of 574 x 574 x 8154 in 10 Hz. Statistical scores are shown in mean ± SD across n = 5 recordings from a single mouse. Box plot elements as in **b**.

e. F1, precision, and sensitivity scores of segmentation by CNMF-E are 0.74 ± 0.06, 0.58 ± 0.07, and 0.88 ± 0.04, respectively. F1, precision, and sensitivity scores of segmentation by DeepWonder are 0.87 ± 0.10, 0.91 ± 0.09, and 0.88 ± 0.07, respectively. Statistical scores are shown in mean ± SD across n = 5 recordings from a single mouse. Height of bars: Mean. Error bars: SD. Black dots: n = 5 recordings.

f. Spatial (left) and temporal (right) components of 10 example neurons detected by both methods.

g. The spatial (top) and temporal components (bottom) of 24 neurons that are only detected by DeepWonder. All of them show good quality. The window size of each component is 24 μm.

h. The signal-to-noise ratio (SNR) of all neurons detected by both methods. Colors match the example traces shown in **f**. Data from n = 247 neurons in a single recording.

i. The full width at half maximum (FHWM) of components detected by both CNMF-E (left) and DeepWonder (right). The black dashed line represents FWHM of 10 μm, which is the average diameter of labeled neurons in the mouse brain. Data from n = 247 neurons in a single recording. Box plot elements as in **b**.

Scale bar: 100 μm in **a** and **c**, 20 seconds in **f**, and 10 seconds in **g**.

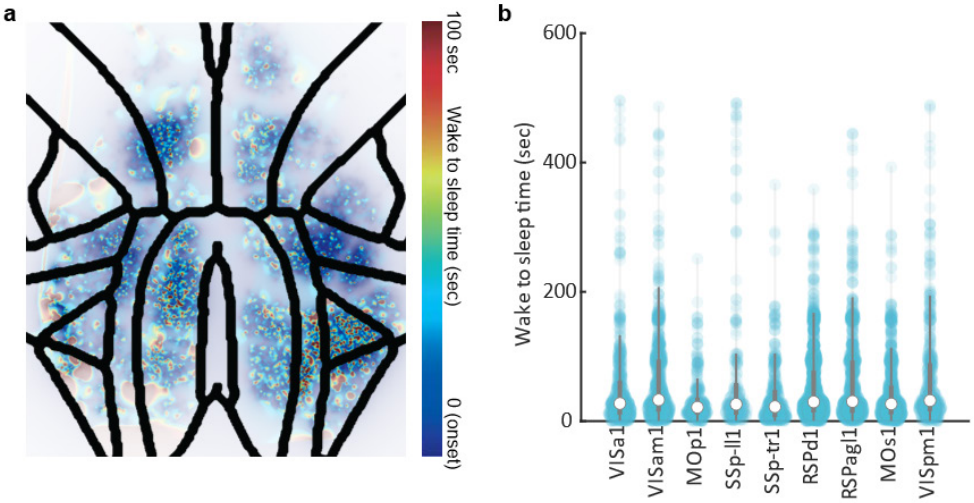

Supplementary Figure 20

Various wake-to-sleep latency distributions across multiple brain area.

a. Rendering of wake-to-sleep latency in second scale across multiple brain areas. For each neuron inferred by DeepWonder, we firstly conduct a calcium deconvolution with OASIS[4] to get the spike trains, then calculate the firing rate in a bin width of 10 seconds. We define the neuron to be fully "asleep" when its firing rate drops to be lower than 10% of its peak firing rate. We calculate the wake-to-sleep latency by counting the time between the isoflurane onset and the time point that the neuron is fully asleep. We use *interp2* in MATLAB to conduct 2D interpolation distribution of wake-to-sleep latency with the neuron density controlling the transparency.

b. Wake-to-sleep latency distributions across multiple brain regions in violin plots. n = 5193 neurons across 9 cortical regions in 1 recording were evaluated. White circle: Median. Thick grey vertical line: Interquartile range. Thin vertical lines: Upper and lower proximal values. Transparent disks: Data points. Transparent violin-shaped areas: Kernel density estimate of data distribution.

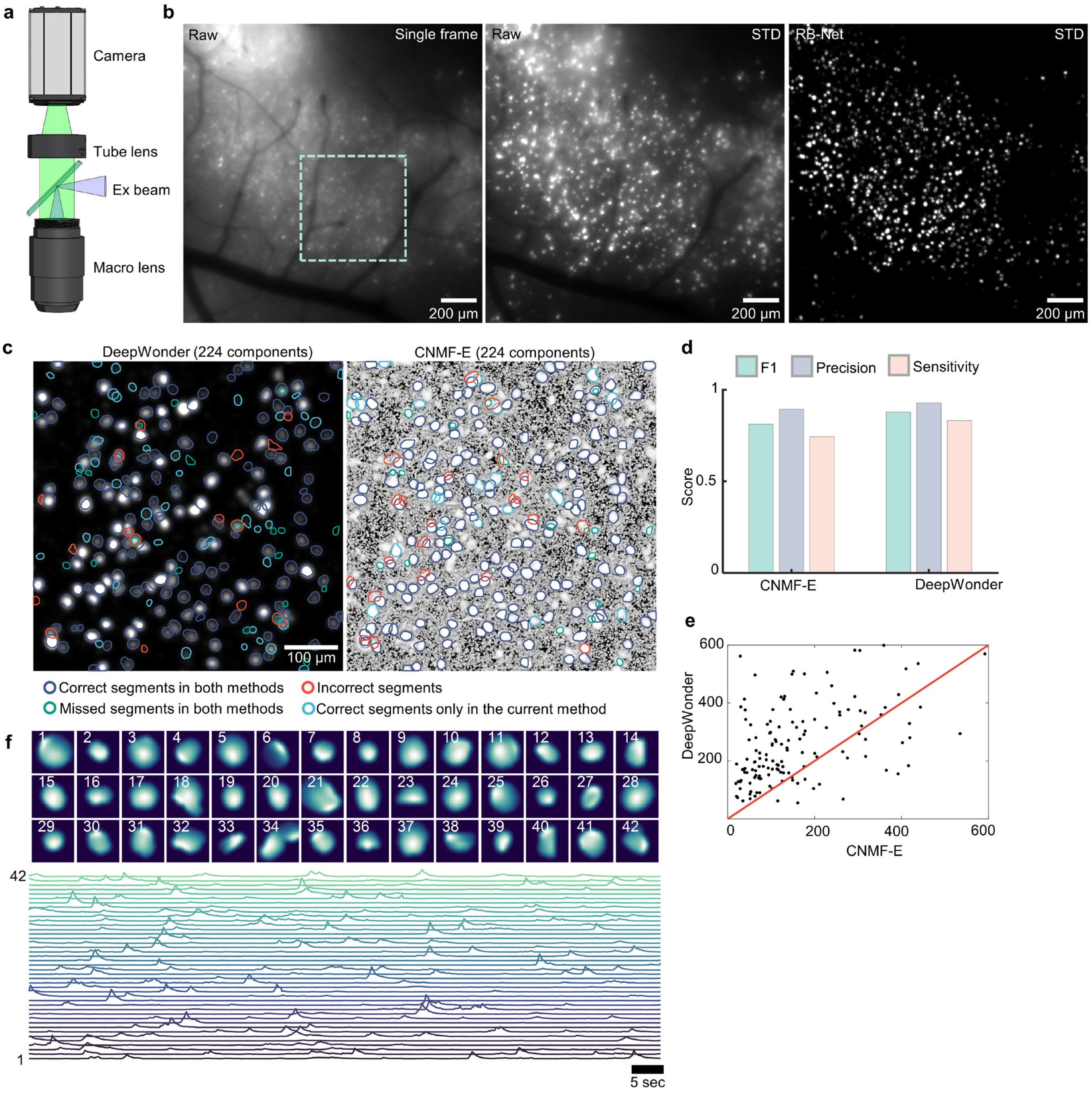

Supplementary Figure 21

Comparisons of DeepWonder and CNMF-E in macroscope dataset.

a. Sketch of a macroscope system.

b. Left to right: a single frame of raw recordings, standard deviation (STD) of raw recordings, and STD of background removed images by proposed RB-Net. All images are up-sampled 4 times for better visibility.

c. The contour plot of all neurons detected by DeepWonder (left) and CNMF-E (right) superimposed on the STD of background removed images and correlation image from CNMF-E, respectively. Compare to manual segmentation, deep blue circles mark correct segments in both methods, red circles mark incorrect segments in each the methods, green circles mark missed segments in both methods, and shallow blue circles mark correct segments that are only in the current method.

d. F1, precision, and sensitivity scores of segmentation by CNMF-E are 0.81, 0.89, and 0.74, respectively. F1, precision, and sensitivity scores of segmentation by DeepWonder are 0.88, 0.93, and 0.83, respectively.

e. The signal-to-noise ratio (SNR) of all neurons detected by both methods. n = 145 neurons.

f. The spatial (top) and temporal components (bottom) of 42 neurons that are only detected by DeepWonder only. All of them show good quality in spatial footprints and temporal activities. The window size of each component is 24 μm.

Scale bar: 200 μm in **a**, 100 μm in **c**, and 5 seconds in **f**.

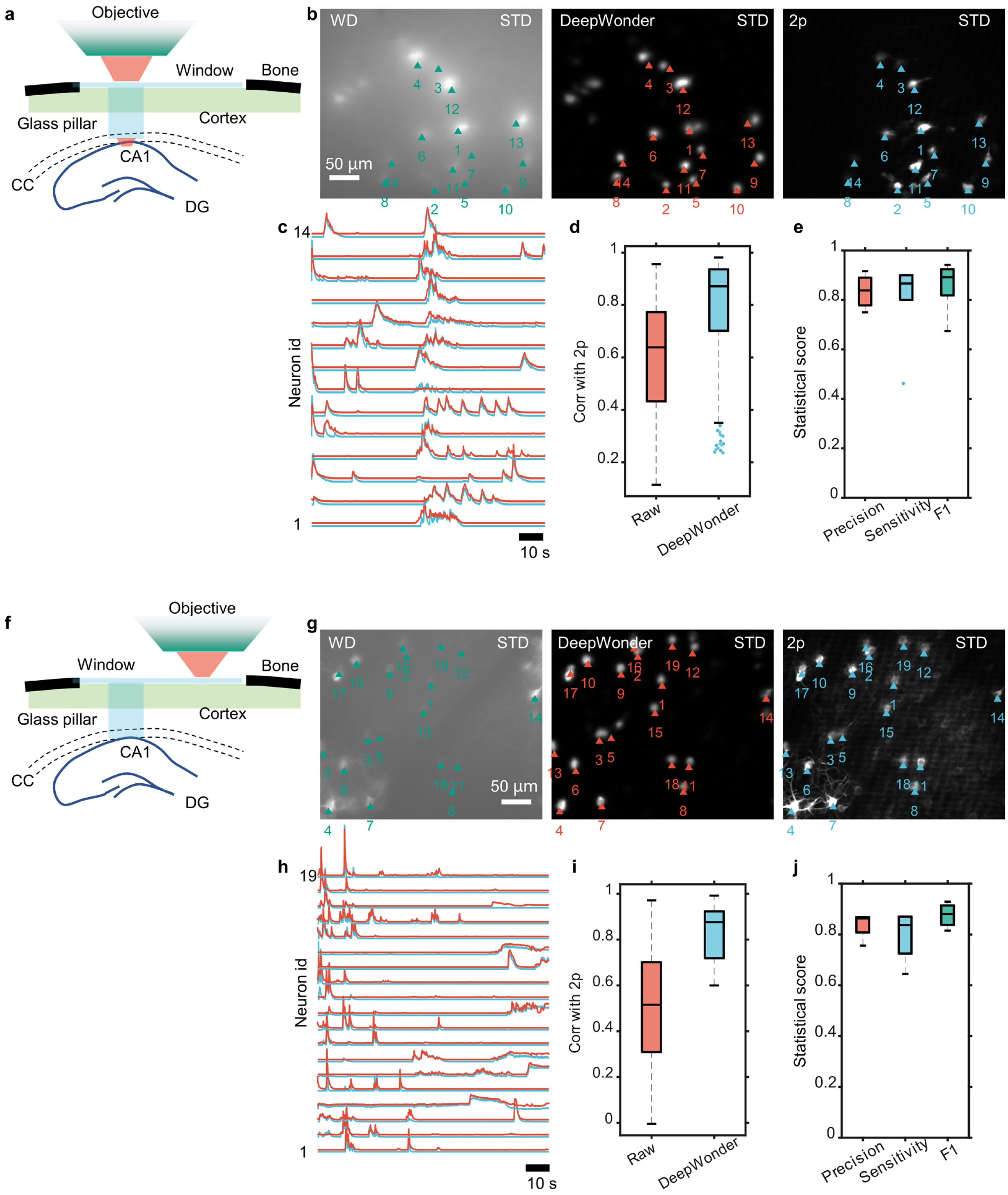

Supplementary Figure 22

DeepWonder in cortical and hippocampal imaging.

a. Simplified schematics of imaging hippocampus CA1 area through a glass pillar by the hybrid imaging system[7] (Methods).

b. Left to right, standard deviation (STD) image across temporal frames of raw widefield movie, DeepWonder processed movie, and 2p movie, respectively. Active neurons are labeled by arrows and numbers in each panel, where green for raw movie, red for DeepWonder movie, and blue for 2p movie.

c. Neuronal activity traces corresponding to arrows in STD panels as used for performance quantifications. Red traces for DeepWonder movie and blue traces for 2p movie, and DeepWonder traces are offset vertically for clarity.

d. Temporal correlations of detected neurons with 2p from DeepWonder output movie (0.81 ± 0.19, mean ± SD, blue) and raw movie (0.60 ± 0.22, mean ± SD, red), across n = 232 neurons from 4 recordings in 1 mouse. Central black mark: Median. Bottom and top edges: 25th and 75th percentiles. Whiskers extend to extreme points excluding outliers (1.5 times above or below the interquartile range).

e. F1, precision, and sensitivity scores of segmentation by DeepWonder are 0.89 ± 0.07, 0.84 ± 0.05, and 0.87 ± 0.11 across n = 4 recordings in 1 mouse, respectively. Box plot elements as in **d**.

f. Simplified schematics of imaging cortex in the same mouse.

g, **h** are the same as **b**, **c** but from cortical imaging data.

i. Temporal correlations of detected neurons with 2p from DeepWonder output movie (0.82 ± 0.12, mean ± SD, blue) and raw movie (0.50 ± 0.25, mean ± SD, red), across n = 254 neurons from 3 recordings in 1 mouse. Box plot elements as in **d**.

j. F1, precision, and sensitivity scores of segmentation by DeepWonder are 0.88 ± 0.04, 0.86 ± 0.04, and 0.84 ± 0.08 across n = 3 recordings in 1 mouse, respectively. Box plot elements as in **d**.

Scar bar: 50 μm and 10 seconds.

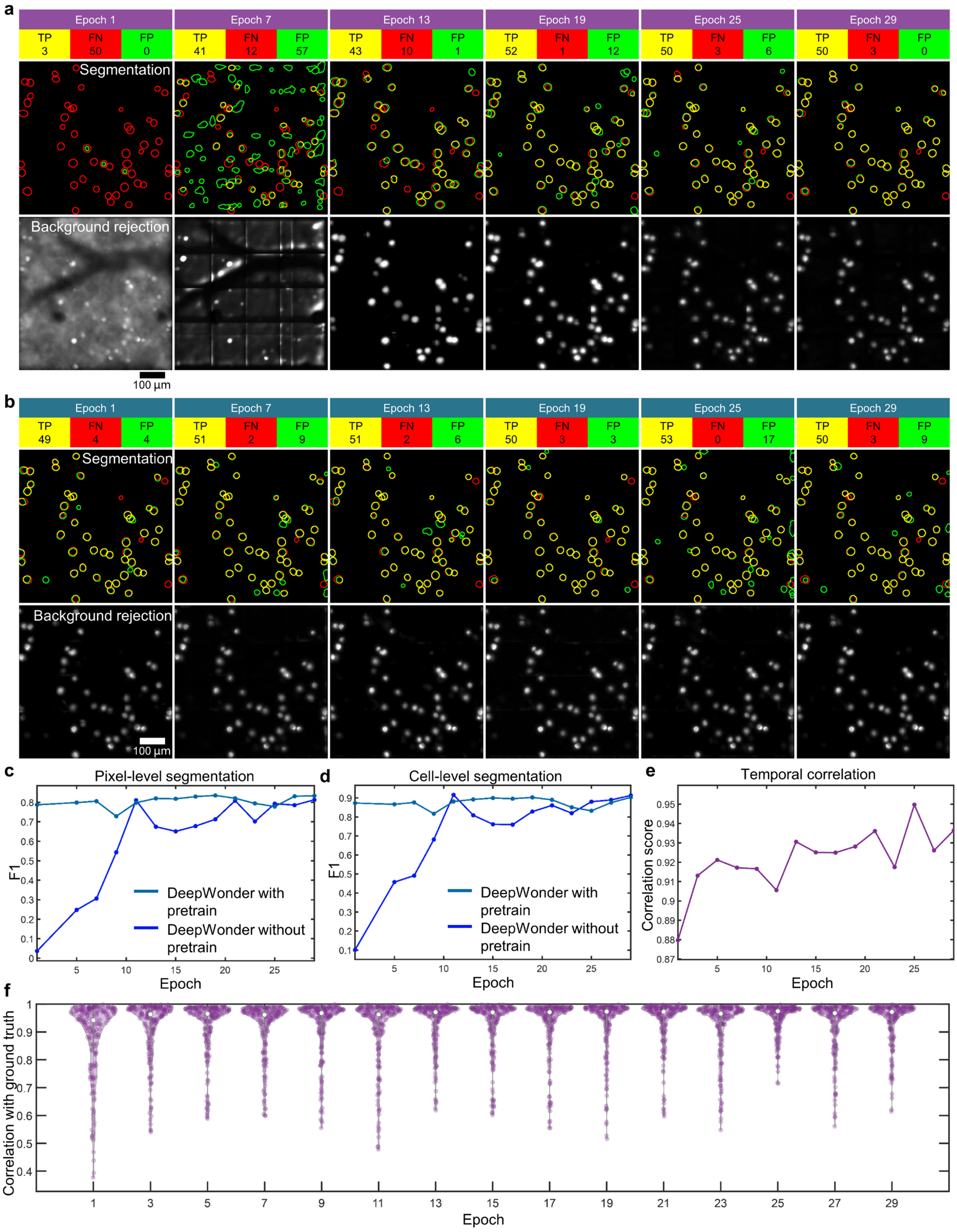

Supplementary Figure 23

Fast fine tuning DeepWonder in new datasets through a pretrained model.

a. The neuron segmentation results of DeepWonder without the pretrained model across different training epochs. The first row, training epoch number. The second row, the number of true positives (TP), false negatives (FN), and false positives (FP) in the test dataset. The third row, the neuron segmentation results of DeepWonder without the pretrained model, where true positives (yellow circles), false negatives (red circles), and false positives (green circles) are labeled. The fourth row, the standard deviation (STD) of DeepWonder output background removed movies.

b. The same as **a** but by the DeepWonder which is initialized with the pretrained model.

c. Pixel-level neuron segmentation statistics (F score) across different epochs in the same test datasets by DeepWonder with (aquamarine) and without (blue) the pretrained model. The statistical F1 scores in pixel level are reported by judging if a segmented pixel from tested methods have the same class as the corresponding pixel in ground truth data.

d. Cell-level neuron segmentation statistics (F score) across different epochs in the same datasets by DeepWonder with (aquamarine) and without (blue) the pretrained model. The statistical F1 scores in cell level are reported by judging if a segmented “neuron” from tested methods is also labeled as “neuron” in ground truth data.

e. Correlation scores increase as the fine-tuning DeepWonder continues.

f. The violin plot of the distribution of correlation scores of DeepWonder with the pretrained model at different training epochs. As DeepWonder is fine-tuned, the distribution of correlation scores is more concentrated. The distribution is highly concentrated over 0.9 after 5 epochs of training. Data from 187 neurons from 3 simulated single recording. White circle: median. Vertical grey bar: interquartile range. Transparent disks: data points. Transparent violin-shaped areas: kernel density estimate of data distribution. Data from 260 neurons across 3 recordings over 2 mice.

Scale bar: 100 μm.

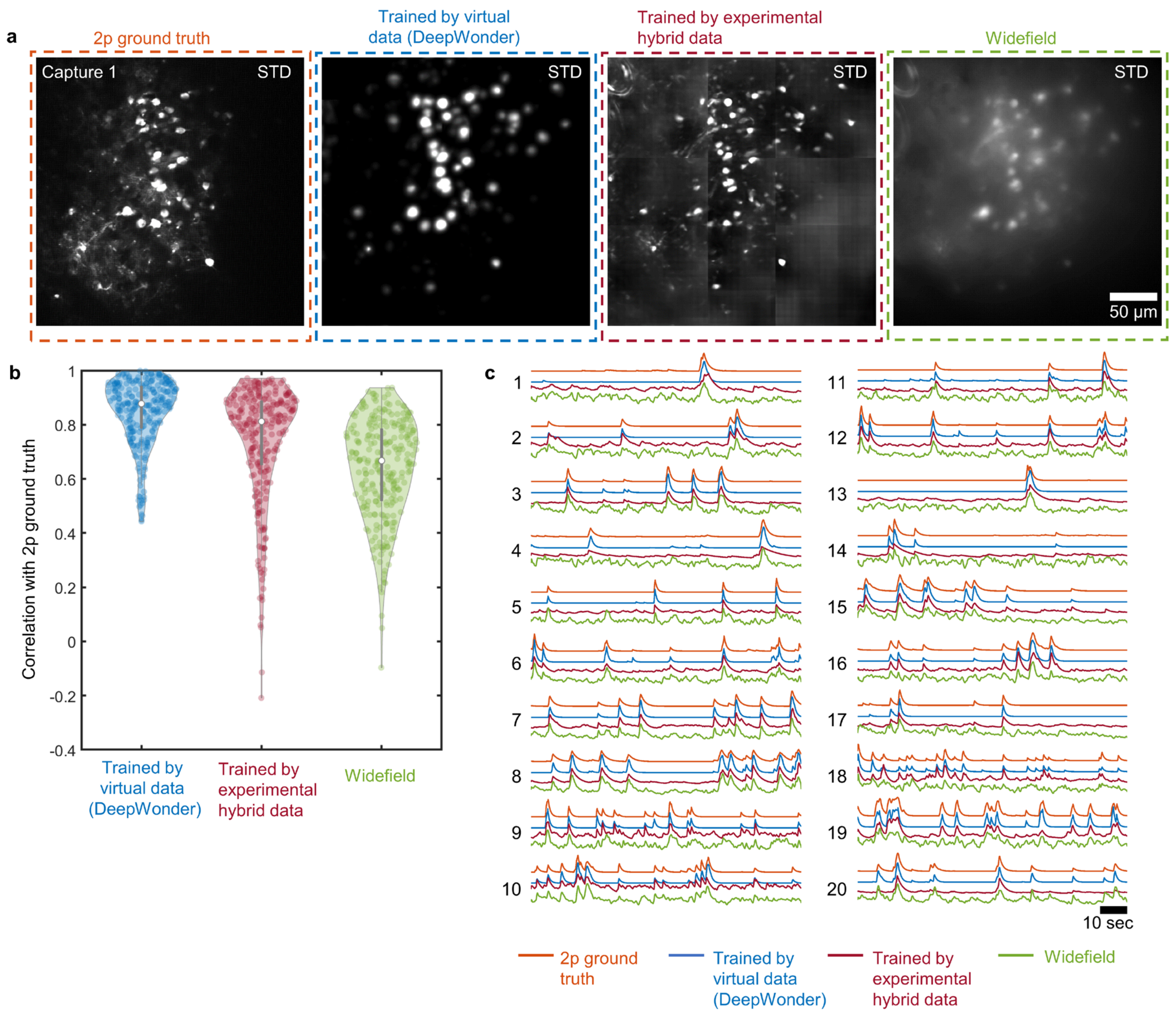

Supplementary Figure 24

Comparisons of a neural network trained by experimental paired hybrid data with DeepWonder.

a. Left to right: standard deviation (STD) of experimentally captured 2p movie by the hybrid microscope, STD of DeepWonder output movie, STD of a neural network trained by paired hybrid data (i.e. from experimental widefield movie to experimental 2p movie), STD of experimentally captured widefield movie by the hybrid microscope.

b. Distributions of temporal correlation with 2p ground truth by DeepWonder-extracted traces (blue), traces from neural network trained by experimental hybrid data (red), and raw traces (green). Mean ± SD of correlation scores are 0.85 ± 0.12, 0.74 ± 0.22, 0.64 ± 0.19, respectively. White circle: median. Vertical grey bar: interquartile range. Transparent disks: data points. Transparent violin-shaped areas: kernel density estimate of data distribution. Data from 260 neurons across 3 recordings over 2 mice.

c. 20 exemplary traces from 2p ground truth (orange), DeepWonder (blue), neural network trained by experimental hybrid data (deep red), and raw movie (green).

Scale bar: 50 μm and 10 seconds.

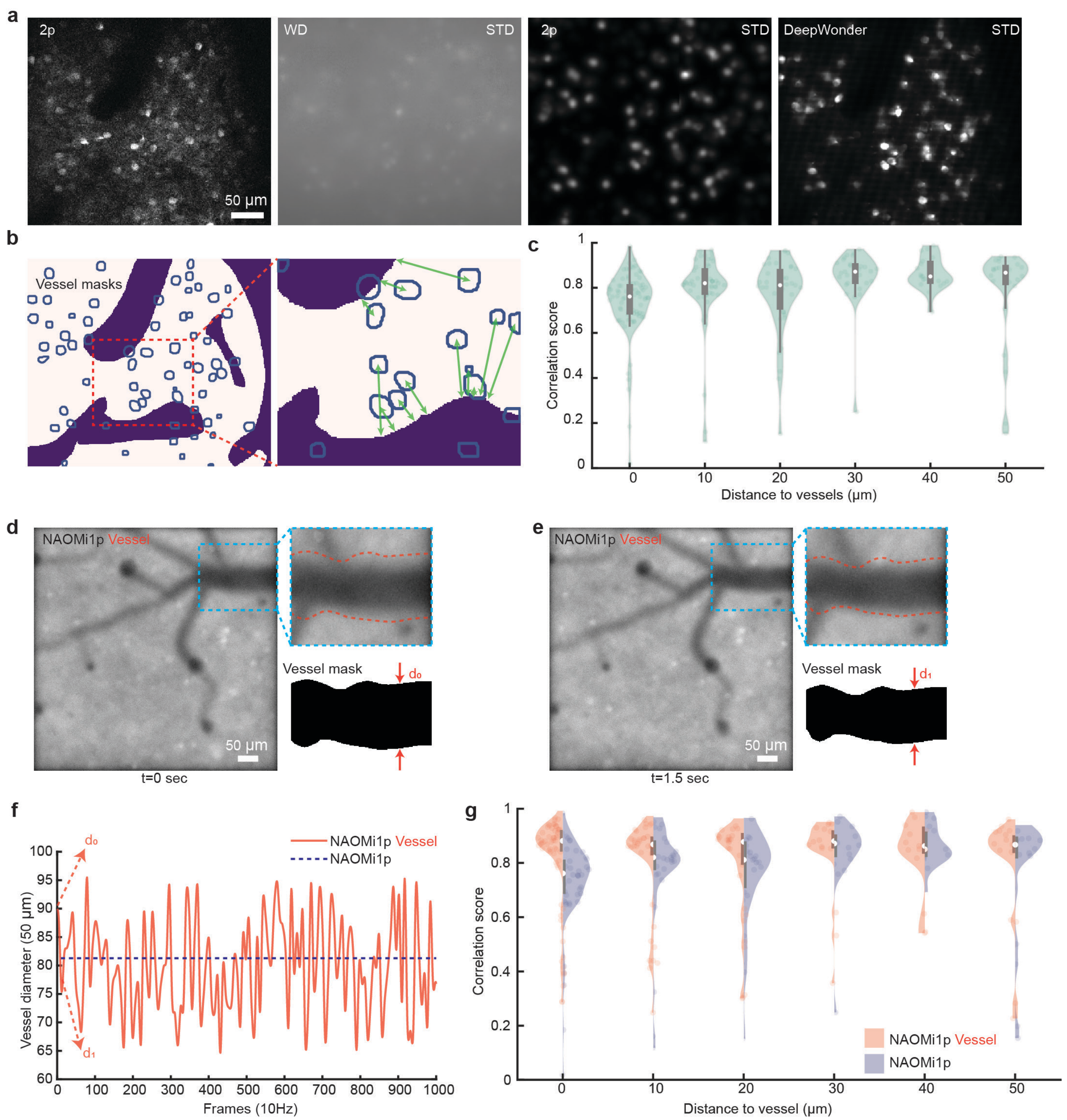

Supplementary Figure 25

Incorporating hemodynamics modeling in NAOMi1p model.

a. Left to right in the first row: an exemplary frame from 2p capture, standard deviation (STD) across the temporal dimension of raw widefield movie (WD), DeepWonder processed movie, and 2p movie.

b. Left, manually annotated vessel masks (purple) and segmented neuron masks (blue). Right, a zoom-in panel where the distances between neurons and vessels are labeled by green arrows.

c. Correlation scores between DeepWonder and 2p functional ground truth as a function of neuron-vessel distance by DeepWonder. Correlation scores are 0.74 ± 0.15, 0.79 ± 0.17, 0.77 ±0.17, 0.85 ± 0.13, 0.86 ± 0.07, 0.78 ± 0.22 in neuron-vessel distance at 0, 10, 20, 30, 40, 50 μm, respectively. Data from n = 4 recordings across 3 mice. White circle: Median. Thick grey vertical line: Interquartile range. Thin vertical lines: Upper and lower proximal values. Transparent disks: Data points. Transparent violin-shaped areas: Kernel density estimate of data distribution.

d. An exemplary frame output by NAOMi1p-Vessel at t = 0 second (Methods). The blue box shows the zoom-in image of a fragment of vessels (top right), and corresponding vessel masks are plotted in the bottom right. The boundary of the vessel mask is overlaid with red dashed lines in the zoom-in image.

e. The same as **d** but at t=1.5 second, where the vessel fragments are contracted obviously.

f. Diameters of the vessel fragments change as a function of time across 1000 frames at 10 Hz (solid red lines). The diameter values from **d** (d_0) and **e** (d_1) are labeled by dashed red arrows. As a comparison, the diameters of the same vessel fragment by the NAOMi1p without hemodynamics modeling are consistent across different time (dashed blue lines).

g. Correlation scores between NAOMi1p-vessel trained DeepWonder and 2p ground truth (red), and correlation scores between initial DeepWonder and 2p ground truth (blue), as a function of neuron-vessel distance. Correlation scores of DeepWonder trained by NAOMi1p-vessel are 0.83 ± 0.16, 0.81 ± 0.17, 0.81 ±0.17, 0.85 ± 0.13, 0.85 ± 0.13, 0.81 ± 0.18 in neuron-vessel distance at 0, 10, 20, 30, 40, 50 μm, respectively. Correlation scores of DeepWonder trained by initial NAOMi1p are 0.74 ± 0.15, 0.79 ± 0.17, 0.77 ±0.17, 0.85 ± 0.13, 0.86 ± 0.07, 0.78 ± 0.22 in neuron-vessel distance at 0, 10, 20, 30, 40, 50 μm, respectively. Data from n = 4 recordings across 3 mice. Violin plot elements as in **c**.

Scale bar: 50 μm.

a

b

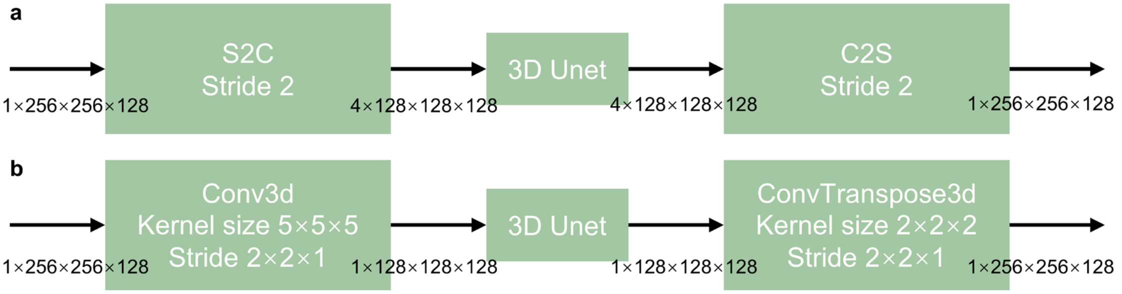

c

	Pixel level			Cell level		
	F1	Recall	Precision	F1	Recall	Precision
S2C	0.85563	0.87862	0.83448	0.87954	0.96234	0.80986
Large CONV+Stride	0.70275	0.76597	0.64937	0.73796	0.86611	0.64286

d

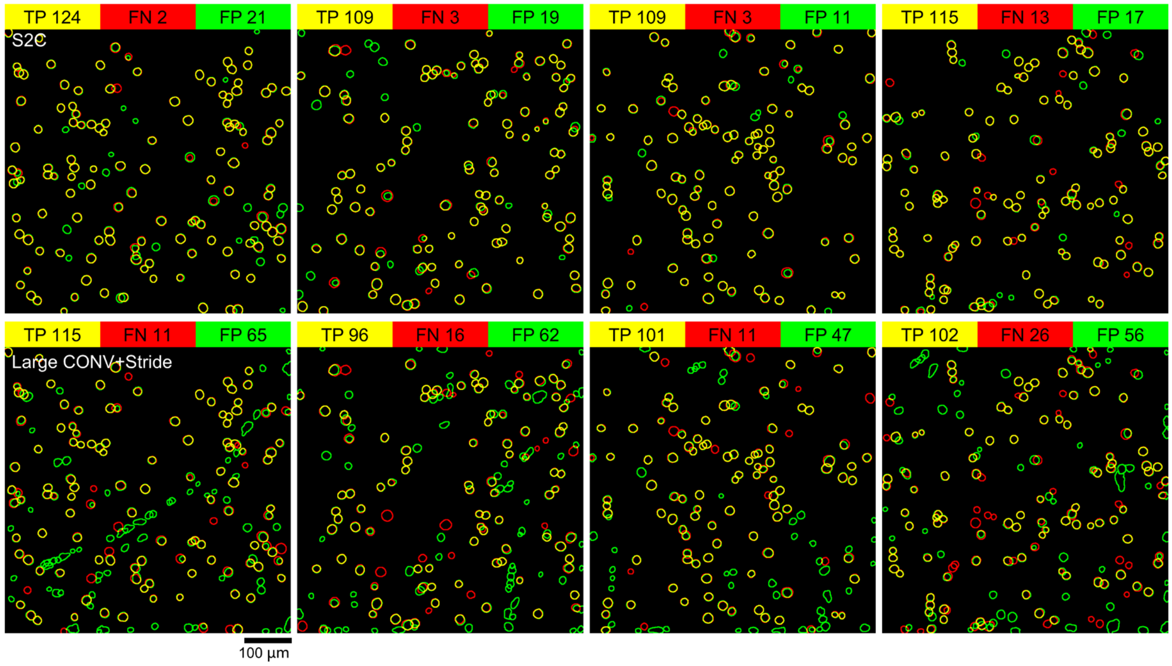

Supplementary Figure 26

Comparison of S2C with a large convolutional filter and a large stride.

a. The scheme of DeepWonder with “spatial to channel” (S2C) and “channel to spatial” (C2S) operators. The input data size for 3D-Unet in DeepWonder is 4(C) x 128(H) x 128(W) x 128(T).

b. The scheme of a tested neural network which has the same input and output size as **a** but replaces S2C and C2S by a large convolutional filter (CONV) and a large stride. The input data size for 3D-Unet is 1(C) x 128(H) x 128(W) x 128(T). Note the total voxel number of input data in **b** is the same as that in **a**, and the run time of **a** and **b** is similar

c. Accuracy comparison of DeepWonder and the tested neural network with a large CONV and a large stride in neuron segmentation. The statistical F1, recall, and precision score in pixel level are reported by judging if a segmented pixel from tested methods have the same class as the corresponding pixel in ground truth. The statistical F1, recall, and precision score in cell level are reported by judging if a segmented “neuron” from tested methods is also labelled as “neuron” in ground truth data. The scores are averaged across 6 test samples.

d. Neuron segmentation examples by DeepWonder (the first row) and the tested neural network with a large CONV and a large stride (the second row). DeepWonder has fewer false positives and fewer false negatives.

Scale bar: 100 μm.

Supplementary Notes

Supplementary Note 1: Other widefield neuron extraction and activity inference methods.

We compare multiple popular widefield calcium analysis methods with our proposed method in both performance and speed aspects.

CNMF-E. CNMF-E was proposed to extract calcium signals from background-contaminated widefield signals[6]. For each pixel in the raw frame, CNMF-E builds a background model which represents that pixel by a weighted summation of surrounding pixels in a ring shape centered at that pixel. Combining the background model and constrained non-negative matrix factorization (CNMF), CNMF-E is popular in processing both widefield data and microendoscope data. To compare our methods with CNMF-E, we fetched the CaImAn package from the official github repo (https://github.com/flatironinstitute/CaImAn). We used *correlation_pnr* function to calculate correlation images. We used the demo script *demo_pipeline_cnmfE.py* to run our data. CNMF-E ran in batch mode with default batch configuration parameters, including a patch size of 128 x 128 and a patch overlapping size of 20. We adjusted the neuron size parameter (*gSig*) in CNMF-E such that it represented a neuron with 10 μm diameter in the capture. The hyperparameters, i.e. spatial threshold θ_{sp}, signal-to-noise ratio (SNR) threshold θ_{SNR}, cross-correlation threshold L_{min}, peak-to-noise ratio (PNR) threshold P_{min} were set by default values.

OnACID-E. We accessed OnACID-E from the official release of CaImAn. OnACID-E was recently developed as the online version of CNMF-E, which was previously successful on microendoscope data[8]. Since the performance of OnACID-E would not be better than offline CNMF-E[8], we didn't further compare its performance with our method in the manuscript. For running speed, OnACID-E achieves superior speed compared to offline CNMF-E in microendoscope data. However, we found OnACID-E was much slower than CNMF-E on data with a larger pixel number (e.g. 600 x 600 pixels laterally). With default parameters of OnACID-E (from script *demo_online_cnmfE.ipynb* in the CaImAn package), a motion-corrected video with 600 x 600 x 1000 frames took ~3700 seconds for OnACID-E, while only 235 seconds for CNMF-E. We thus compared the speed of CNMF-E with our methods instead of OnACID-E.

Ring-CNN. We accessed ring-CNN from the official release of CaImAn. Ring-CNN technique was recently proposed to accelerate one-photon calcium signal extraction. It firstly removes the background of widefield signals with several trainable ring masks and then uses the established two-photon signal analysis method (CNMF) to extract signals. In ring-CNN method, the background is simply modeled by the convolution of ring masks and the current raw data, which is a simplified strategy compared to the original CNMF-E approach. Our proposed removing background network (RB-Net) in DeepWonder has superior background suppression ability ands achieves clearer neuronal signals (Supplementary Fig. 5). Furthermore, our neuron segmentation network (NS-Net) shows superior performance compared to CNMF which is used in the ring-CNN approach (Supplementary Fig. 7). Since the performance of ring-CNN will not be better than offline CNMF-E[8], we don't further compare its performance with our method in the manuscript. For microendoscope data with a small pixel number, ring-CNN processing speed can exceed the capture speed of video[8]. However, we found ring-CNN was much slower than CNMF-E on the data with a larger pixel number (e.g. 600 x 600 pixels laterally). With default parameters of ring-CNN, a video with 600 x 600 x 1000 frames took ~11000 seconds for ring-CNN, while only 235 seconds for CNMF-E. We further note that for each capture, ring-CNN needs to be re-trained, which takes longer time than following de-background processing. On the other hand, for the same acquisition system, our method needs to be trained once and then can be directly applied to the following data, which saves large amount of time.

MIN1PIPE. We accessed MIN1PIPE from the official release in github https://github.com/JinghaoLu/MIN1PIPE. MIN1PIPE method was initially proposed for processing microendoscope data[9]. We found the running time of MIN1PIPE was significantly longer than CNMF-E in data with a larger pixel number. For data with 750 x 750 pixels and 1000 frames, the diffusion filtering method in MIN1PIPE show a significantly larger background compared to our method (Supplementary Fig. 5). For a standard FOV of 1080 x 1080 pixels with 1000 frames, MIN1PIPE took over 5 hours, while our approach took ~4 minutes and CNMF-E took ~33 minutes. For a dataset with a larger size, e.g. 2.0 x 1.7mm^2 FOV from a single RUSH camera, min1pipe took over 10 hours so we don't further compare speed with our methods.

Supplementary Note 2: Joint one-photon and two-photon data acquisition.
After the joint one-photon and two-photon microscope was set up, we conducted several measurements and adjustments to make sure two-photon and one-photon imaging FOVs are aligned both laterally and axially.

To align the focal plane of two-photon and one-photon microscope, we firstly let the two-photon microscope focus on a planar plant slice whose thickness was less than 10 µm. Then, we moved the tube lens of the widefield microscope such that the plant slice was also focused in the camera. After the above coarse alignment, we further deposited a single layer of 1 µm fluorescent beads on the top of a microscope slide, and drove the piezo scanner to scan focal stacks for both two-photon and one-photon microscope. We separately calculated the best focusing depth of two microscopes and accordingly tuned the tubelens of widefield microscope based on the system magnifications such that both modalities had the same focus depth.

After adjusting the two-photon and one-photon imaging modules with the same focal depths, we again captured a sparse layer of 1 µm fluorescent beads by two modules, and manually found matched beads in each modality. We further used *imregtform* in MATLAB to estimate an affine matrix from widefield FOV to two-photon FOV, which would be used further in mouse brain recordings to match FOVs.

After mouse recordings, we firstly independently ran NormCorre[10] for correcting motions in both one-photon and two-photon datasets[10]. Then, two datasets were registered into the same FOV using pre-calibrated affine projection. We further ran CaImAn[11] with default parameters for analyzing two-photon datasets in batch mode, and ran CNMF-E and DeepWonder on one-photon datasets. We manually screened neurons in widefield movies and neurons that appeared in the same position in the CaImAn results to generate ground truth. After that, we compared both CNMF-E and DeepWonder with the established ground truth, where a neuron that was within 8 µm towards the centroid of a ground truth neuron was labeled as a true positive neuron, otherwise it would be labeled as a false positive. The F1 scores, precision scores, and sensitivity scores were calculated accordingly. The temporal correlation score of a paired neuron (true positive) was computed between the inferred widefield neuron activity and the CaImAn inferred two-photon neuron activity.

Supplementary Note 3: Utilizing pre-trained DeepWonder model for quickly adapting to other datasets

To ensure effortless retrain the DeepWonder, we open source a basic model trained from a large number of diverse datasets which allows the users to fine-tune DeepWonder in specific modalities quickly (https://github.com/yuanlong-o/Deep_widefield_cal_inferece). The pretrained DeepWonder was trained based on following RUSH[12] specifications:

- Acquisition NA 0.35, dry objective
- Excitation wavelength centered at 488 nm, emission wavelength centered at 520 nm, GCaMP6 indicator
- Camera pixel size 0.8 µm
- Optical magnification 10x
- Frame rate 10 Hz

The pretrained DeepWonder model was trained on over 50 NAOMi1p datasets (each has a size of 600 x 600 x 1000), with various neuron densities, vasculatures, indicator expression levels, and noise. In the next, we demonstrate that the proposed pretrained model helps quickly retraining DeepWonder in new datasets with the camera pixel size (sampling size) of 0.4 µm instead of 0.8 µm.

Firstly, we demonstrate that the DeepWonder with the proposed pretrained model gets quicker convergence compared to that without the pretrain model in neuron segmentation. As shown in Supplementary Fig. 23, we trained DeepWonder initialized with and without the pretrained model for 30 epochs, and evaluated the neuron segmentation in both pixel levels and cell levels. We found that it took at least 12 epochs to get a high neuron segmentation accuracy without initializing the network by the pretrained model (Supplementary Fig. 23a-d). On the other hand, DeepWonder that was initialized with the pretrained model had a high segmentation accuracy from the beginning of training. The faster convergence speed brought by the proposed pretrained model helps users to quickly migrate DeepWonder into different imaging modalities (in this case, different spatial sampling rates).

Secondly, we demonstrate fine-tuning with the proposed pretrained model achieves satisfactory performances in temporal activity inference within 5 epochs. As shown in Supplementary Fig. 23e, the correlation with ground truth signals from DeepWonder output quickly went higher than 0.91 after 5 epoch training. We visualized the distribution of correlations in violin plots, where the distribution was highly concentrated over 0.9 after 5 epoch training (Supplementary Fig. 23f). We thus demonstrate that our proposed pretrained model helps DeepWonder be distributed into different imaging modalities quickly, and achieve uncompromised performances on both segmentation and signal inference.

Reference

1 Zeiler, M. D. & Fergus, R. in *Computer Vision–ECCV 2014.* 818-833.

2 Soltanian-Zadeh, S., Sahingur, K., Blau, S., Gong, Y. & Farsiu, S. Fast and robust active neuron segmentation in two-photon calcium imaging using spatiotemporal deep learning. *Proc. Natl. Acad. Sci. U. S. A.*, doi:10.1073/pnas.1812995116 (2019).

3 Zipfel, W. R., Williams, R. M. & Webb, W. W. Nonlinear magic: multiphoton microscopy in the biosciences. *Nat. Biotechnol.* **21**, 1369-1377, doi:10.1038/nbt899 (2003).

4 Friedrich, J., Zhou, P. & Paninski, L. Fast online deconvolution of calcium imaging data. *PLoS Comput. Biol.* **13**, e1005423, doi:10.1371/journal.pcbi.1005423 (2017).

5 Wang, Q. *et al.* The Allen Mouse Brain Common Coordinate Framework: A 3D Reference Atlas. *Cell* **181**, 936-953 e920, doi:10.1016/j.cell.2020.04.007 (2020).

6 Zhou, P. *et al.* Efficient and accurate extraction of in vivo calcium signals from microendoscopic video data. *eLife* **7**, doi:10.7554/eLife.28728 (2018).

7 Zhuang, C. *et al.* Real-time brain-wide multi-planar microscopy for simultaneous cortex and hippocampus imaging at the cellular resolution in mice. *Biomed. Opt. Express* **12**, 1858-1868, doi:10.1364/BOE.418229 (2021).

8 Friedrich, J., Giovannucci, A. & Pnevmatikakis, E. A. Online analysis of microendoscopic 1-photon calcium imaging data streams. *PLoS Comput. Biol.* **17**, e1008565, doi:10.1371/journal.pcbi.1008565 (2021).

9 Lu, J. *et al.* MIN1PIPE: A Miniscope 1-Photon-Based Calcium Imaging Signal Extraction Pipeline. *Cell Rep.* **23**, 3673-3684, doi:10.1016/j.celrep.2018.05.062 (2018).

10 Pnevmatikakis, E. A. & Giovannucci, A. NoRMCorre: An online algorithm for piecewise rigid motion correction of calcium imaging data. *J. Neurosci. Methods* **291**, 83-94, doi:10.1016/j.jneumeth.2017.07.031 (2017).

11 Giovannucci, A. *et al.* CaImAn an open source tool for scalable calcium imaging data analysis. *eLife* **8**, doi:10.7554/eLife.38173 (2019).

12 Fan, J. *et al.* Video-rate imaging of biological dynamics at centimetre scale and micrometre resolution. *Nat. Photonics* **13**, 809-816, doi:10.1038/s41566-019-0474-7 (2019).
