# Supplementary material for: Rapid detection of neurons in widefield calcium imaging datasets after training with synthetic data
Source: Nat Methods. 2023 Apr 1;20(5):747–54. doi: 10.1038/s41592-023-01838-7 (PMC10172132; doi:10.1038/s41592-023-01838-7)
Supplement: Supplementary file 2 — Reporting Summary [file 41592_2023_1838_MOESM2_ESM.pdf]

nature research

Corresponding author(s): Qionghai Dai

Last updated by author(s): Jan 31, 2022

Reporting Summary

Nature Research wishes to improve the reproducibility of the work that we publish. This form provides structure for consistency and transparency in reporting. For further information on Nature Research policies, see our Editorial Policies and the Editorial Policy Checklist.

Statistics

For all statistical analyses, confirm that the following items are present in the figure legend, table legend, main text, or Methods section.

n/a	Confirmed	
☐	☒	The exact sample size (n) for each experimental group/condition, given as a discrete number and unit of measurement
☐	☒	A statement on whether measurements were taken from distinct samples or whether the same sample was measured repeatedly
☐	☒	The statistical test(s) used AND whether they are one- or two-sided<br>*Only common tests should be described solely by name; describe more complex techniques in the Methods section.*
☒	☐	A description of all covariates tested
☒	☐	A description of any assumptions or corrections, such as tests of normality and adjustment for multiple comparisons
☐	☒	A full description of the statistical parameters including central tendency (e.g. means) or other basic estimates (e.g. regression coefficient) AND variation (e.g. standard deviation) or associated estimates of uncertainty (e.g. confidence intervals)
☐	☒	For null hypothesis testing, the test statistic (e.g. F, t, r) with confidence intervals, effect sizes, degrees of freedom and P value noted<br>*Give P values as exact values whenever suitable.*
☒	☐	For Bayesian analysis, information on the choice of priors and Markov chain Monte Carlo settings
☒	☐	For hierarchical and complex designs, identification of the appropriate level for tests and full reporting of outcomes
☐	☒	Estimates of effect sizes (e.g. Cohen's d, Pearson's r), indicating how they were calculated

Our web collection on statistics for biologists contains articles on many of the points above.

Software and code

Policy information about availability of computer code

Data collection

The widefield imaging data was acquired using Micro-Manager 2.0.0 (Free release). The two-photon imaging data was acquired using ScanImage 5.5R1 (Free release).

Data analysis

All data analyses were performed in Matlab R2020a (Mathworks), and Python 3.6. Signals from widefield recordings were extracted using the DeepWonder pipeline which was published with the manuscript (Supplementary Software 1). HALS algorithm was enlisted in the segmentation step of DeepWonder and was modified based on the implementation in CaImAn package (https://github.com/flatironinstitute/CaImAn-MATLAB, version: commit 9e524e2). NAOMi1p pipeline was developed based on NAOMi package (https://bitbucket.org/adamshch/naomi_sim, version: 2021-01-26) and was published with the manuscript (Supplementary Software 1). Signals from two-photon microscopy recordings were extracted using the CaImAn Matlab package (https://github.com/flatironinstitute/CaImAn-MATLAB, version: commit 9e524e2). Calcium movies (both widefield and two-photon microscope) were registered using the NormCorre package (https://github.com/flatironinstitute/NoRMCorre, version: commit 1b39f82). For background suppression comparisons, the MIN1PIPE package (https://github.com/JinghaoLu/MIN1PIPE, version: commit e6449b5) was used. For segmentation performance comparisons, the SUNS package (https://github.com/YijunBao/SUNS_paper_reproduction, version: commit c7e57f4) and STNeuroNet package (https://github.com/soltanianzadeh/STNeuroNet, version: commit 6537096) were used. For visualization, ImageJ (https://imagej.net/software/fiji/, version 2.9.0) was used.

For manuscripts utilizing custom algorithms or software that are central to the research but not yet described in published literature, software must be made available to editors and reviewers. We strongly encourage code deposition in a community repository (e.g. GitHub). See the Nature Research guidelines for submitting code & software for further information.

Data

Policy information about availability of data

All manuscripts must include a data availability statement. This statement should provide the following information, where applicable:

- Accession codes, unique identifiers, or web links for publicly available datasets
- A list of figures that have associated raw data
- A description of any restrictions on data availability

We have mounted our demo data and codes in Google Colab, which is a free Jupyter notebook environment that requires no setup and runs entirely in the cloud. A demo script with full processing of DeepWonder on several demo datasets (including NAOMi1p virtual datasets, cropped RUSH datasets, and two-photon validation datasets) is available through Colab via https://colab.research.google.com/drive/15TvsyEYgE1iGpaNWkq3flXOw52I51mVa. Over 50 Gb paired 2p and widefield data has been made publicly available through https://drive.google.com/drive/folders/1OBcQUY-vsIPljSBChFfn-zqAYtYvDZ4A?usp=sharing. The Allen CCF atlas is available at http://atlas.brain-map.org.

Field-specific reporting

Please select the one below that is the best fit for your research. If you are not sure, read the appropriate sections before making your selection.

☒ Life sciences ☐ Behavioural & social sciences ☐ Ecological, evolutionary & environmental sciences

For a reference copy of the document with all sections, see nature.com/documents/nr-reporting-summary-flat.pdf

Life sciences study design

All studies must disclose on these points even when the disclosure is negative.

Sample size	Sample sizes, in our case the number of mice and recordings, were chosen to ensure that animal-to-animal and recording-to-recording variability was reflected in the captured data. A total of 113 neuronal recordings from 18 animals were analyzed (including two-photon verification recordings). Only animals were included in the study for which all animal procedures (as described in Methods) worked successfully to allow for signal detection (see next item). Provided that animal procedures (surgeries and viral injections/GECI expression) were successful as verified using a standard two-photon microscope, we found imaging results and data quality to be reliably reproducible and consistent, both across imaging sessions with the same animal, and across animals. These minimum criteria are consistent with or exceed other reports in the literature (Ref. 7, 11, 21). Since the subject of our manuscript is to establish a neural recording and signal extraction method rather than any biological findings, we consider this sample size sufficient to verify the performance of our method.
Data exclusions	Only animals were included in the study for which all animal procedures (as described in Online Methods) worked successfully to allow for signal detection (i.e., GECI expression observable), as verified using a standard two-photon microscope. Of these animals, none were excluded.
Replication	For all animals in which animal procedures (surgeries and viral injections/GECI expression, see Online Methods) were successful (as verified using a standard two-photon microscope), imaging and data analysis results were reliably reproduced, both across imaging sessions with the same animal, and across animals, over the course of 9 months. A total of 113 neuronal recordings from 18 animals.
Randomization	Randomization was not relevant to this study, because no experimental groups were formed.
Blinding	Blinding was not relevant to this study, because no group allocation was performed.

Reporting for specific materials, systems and methods

We require information from authors about some types of materials, experimental systems and methods used in many studies. Here, indicate whether each material, system or method listed is relevant to your study. If you are not sure if a list item applies to your research, read the appropriate section before selecting a response.

Materials & experimental systems

n/a	Involved in the study	
☒	☐	Antibodies
☒	☐	Eukaryotic cell lines
☒	☐	Palaeontology and archaeology
☐	☒	Animals and other organisms
☒	☐	Human research participants
☒	☐	Clinical data
☒	☐	Dual use research of concern

Methods

n/a	Involved in the study	
☒	☐	ChIP-seq
☒	☐	Flow cytometry
☒	☐	MRI-based neuroimaging

Animals and other organisms

Policy information about studies involving animals; ARRIVE guidelines recommended for reporting animal research

Laboratory animals	Mus musculus, C57BL/6J and transgenic mice (Rasgrf2-2A-dCre/Ai148D), male and female, P90+, obtained from The Jackson Laboratory. Mice were housed in standard cages with a maximum of 5 mice per cage. Cages were housed in an environment with a 12/12h reverse dark/light cycle, and ambient temperature of 72F and an ambient humidity of ~30%. Mice were provided food and water ad libitum.
Wild animals	none
Field-collected samples	none
Ethics oversight	All animal experiments were performed following institutional and ethical guidelines for animal welfare and have been approved by the Institutional Animal Care and Use Committee (IACUC) of Tsinghua University.

Note that full information on the approval of the study protocol must also be provided in the manuscript.
