# Supplementary material for: Rapid detection of neurons in widefield calcium imaging datasets after training with synthetic data
Source: Nat Methods. 2023 Apr 1;20(5):747–54. doi: 10.1038/s41592-023-01838-7 (PMC10172132; doi:10.1038/s41592-023-01838-7)
Supplement: Supplementary file 3 — DeepWonder and NAOMi1p computational pipeline, with inline documentation, and demo scripts. See https://github.com/yuanlong-o/Deep_widefield_cal_inferecefor for future updates. [file 41592_2023_1838_MOESM3_ESM.zip › colab_tutorial_e.pdf]

Running a DeepWonder notebook

Save a copy of the Colab notebook into your Google drive.

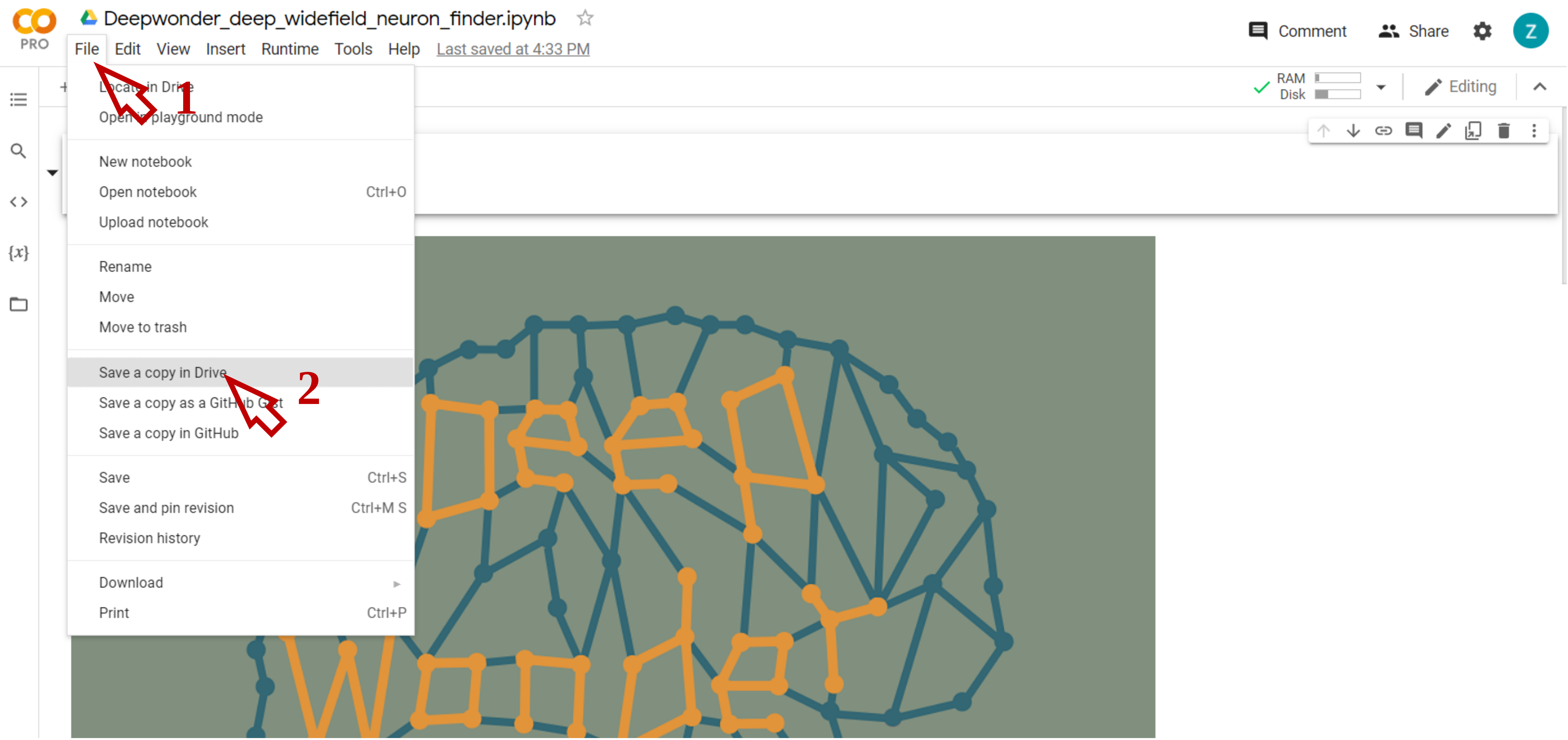

Save a copy of the Colab notebook into your Google drive.

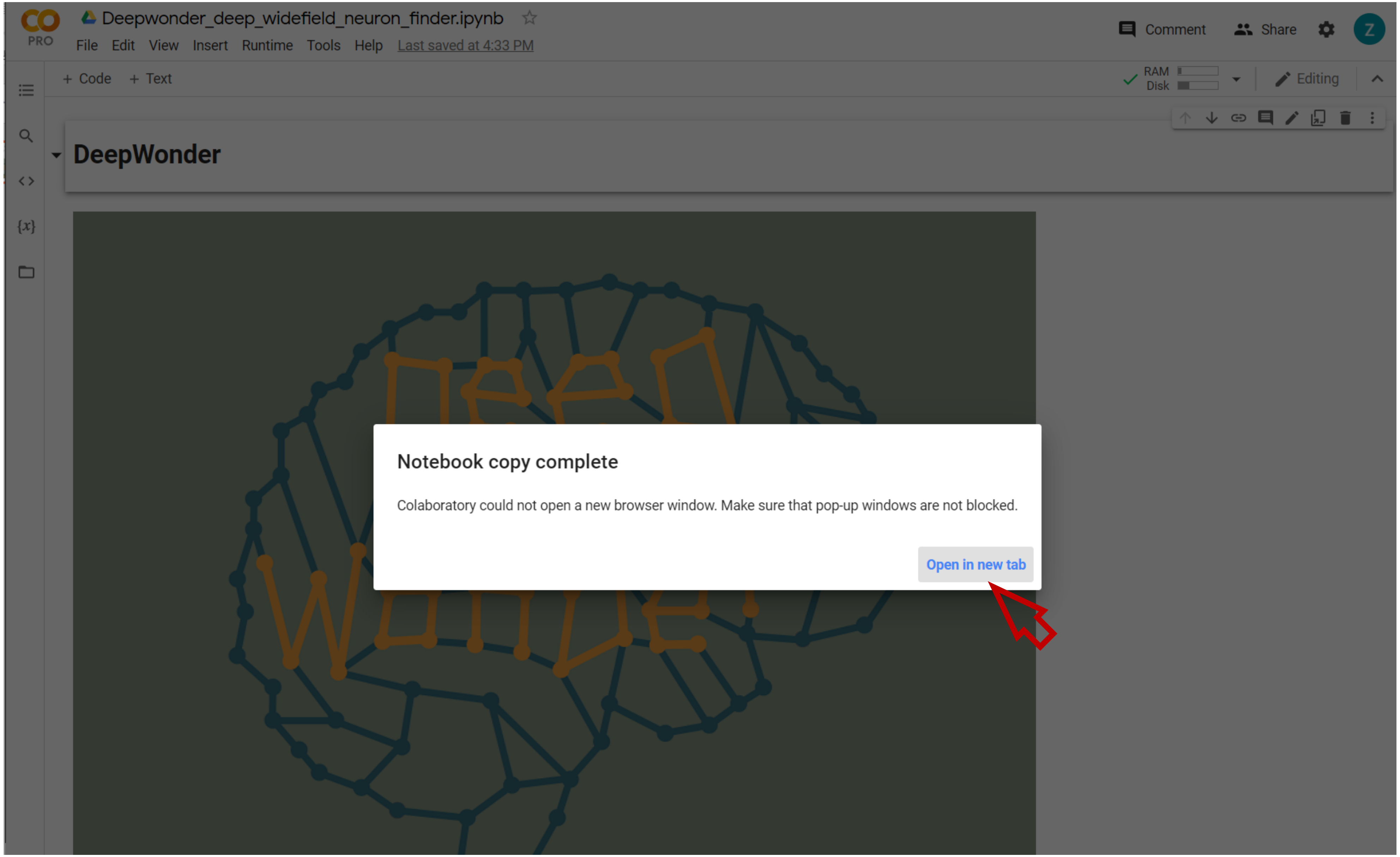

Rename the file according to your preference

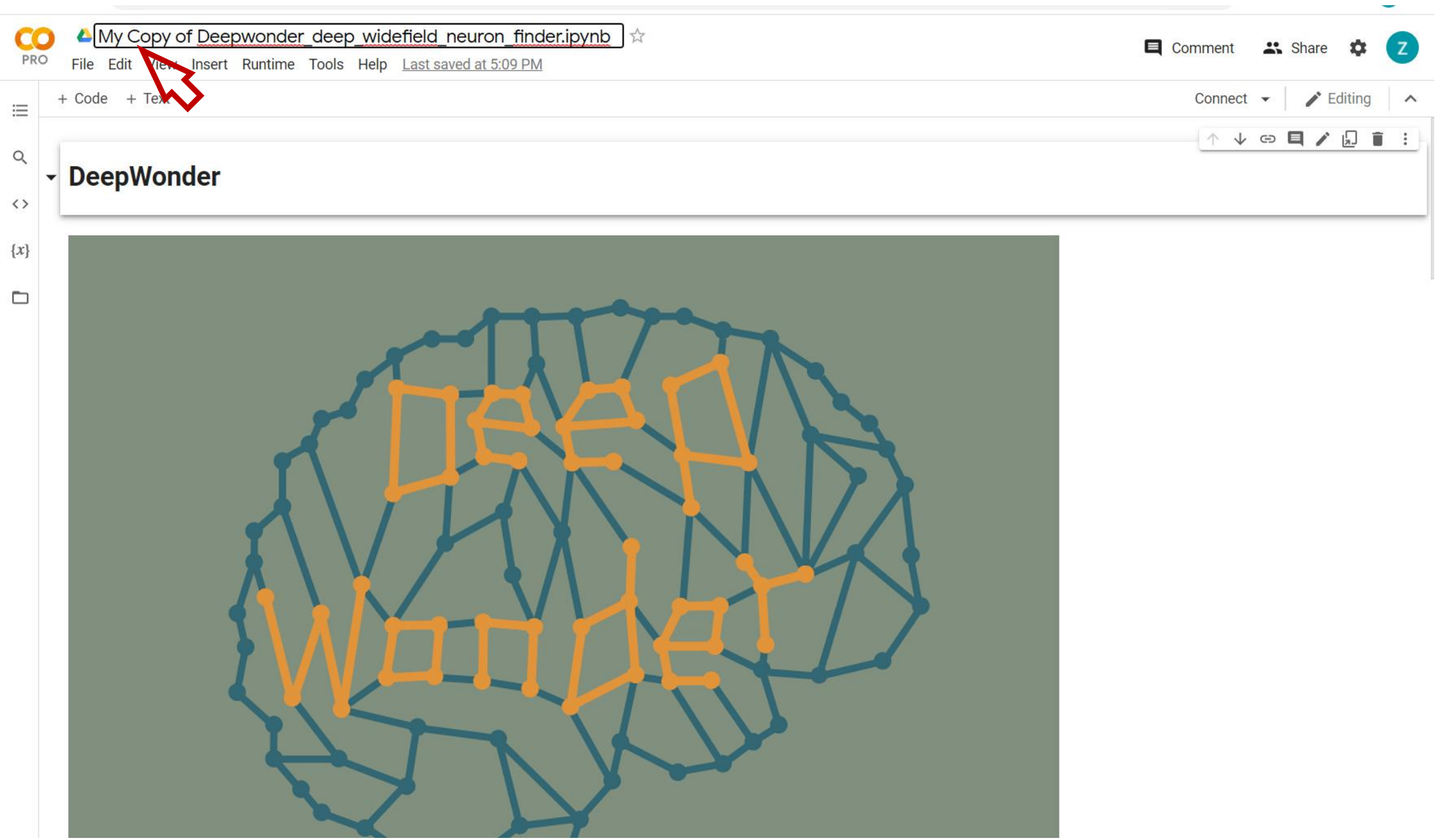

Download the demo data

1.Synthetic widefield data by NAOMi1p code:
https://drive.google.com/drive/folders/1WiTrL5gRuMUssMYt2uDRDO-5pmmrdNSc?usp=sharing

2.Cropped RUSH data:
https://drive.google.com/drive/folders/1CP6CuAmOkAx_hoAhT4h-Pd1o_FTcva9M?usp=sharing

3.Widefield data jointly with two-photon ground truth:
https://drive.google.com/drive/folders/1T7vaOT4tThMumCxi_sFeN5vybv91pl2f?usp=sharing

(Check the link in your browser since sometimes "-" or "_" will miss)

Download the demo data

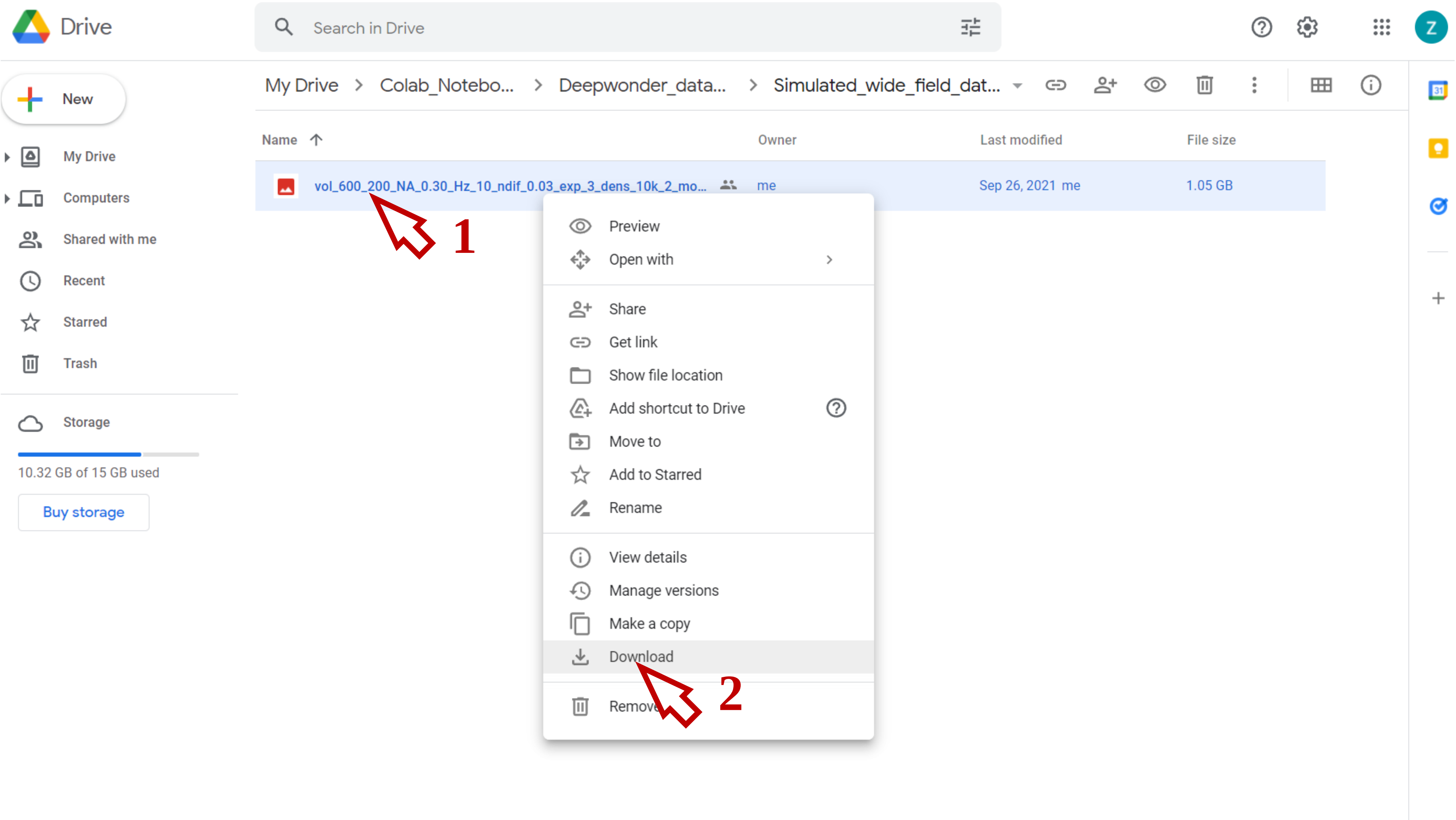

Upload the demo data

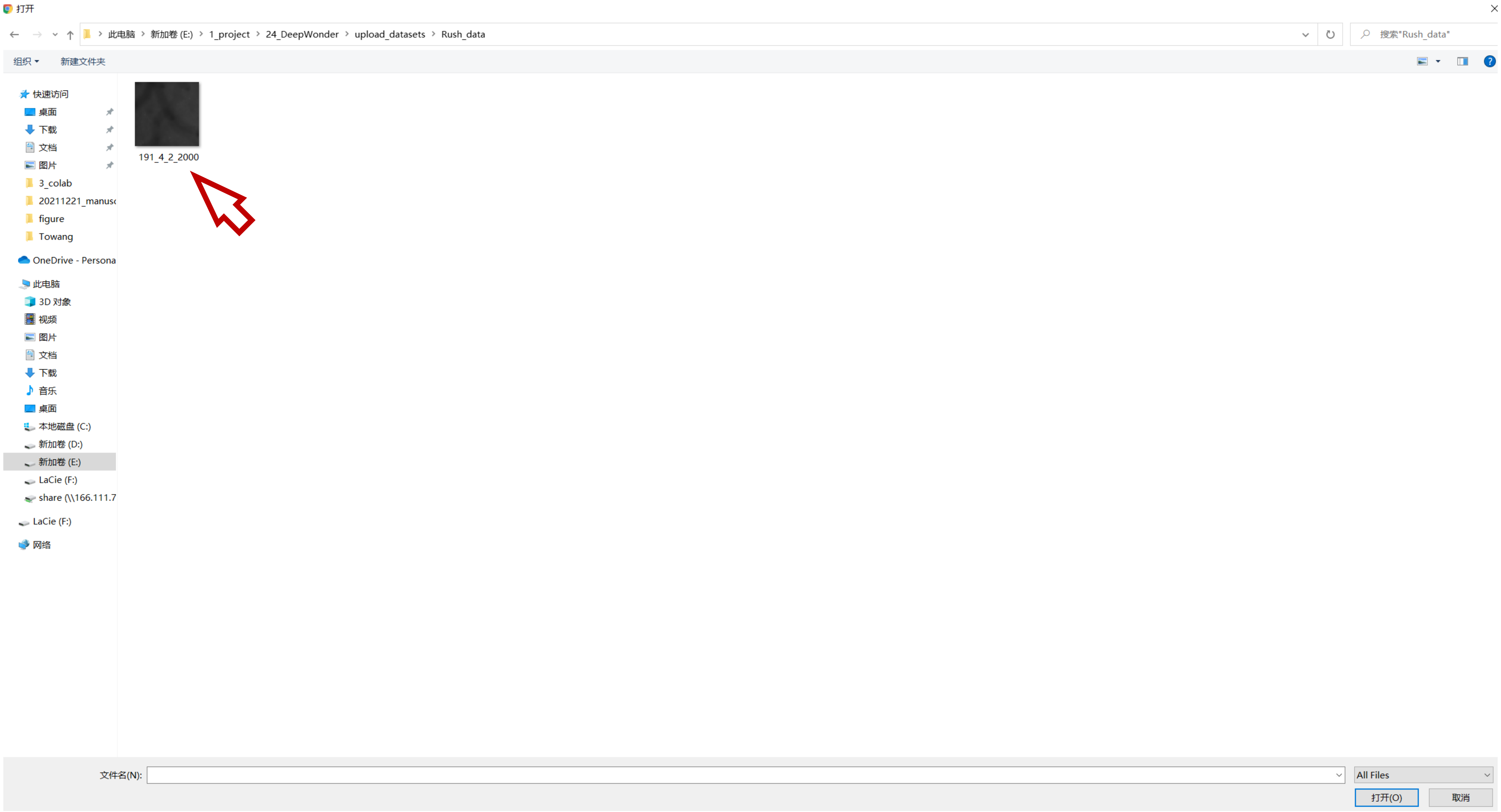

Download the trained models

1.Background removing model:
https://drive.google.com/drive/folders/1K3O1TQAOqAwwiwblF2YS90kFNAqnULwK?usp=sharing

2. Neuron segmentation model:
https://drive.google.com/file/d/1SR7VXdRADTLa3tOfBtTmqEZk2kZEmXId/view?usp=sharing

Download the background removing model

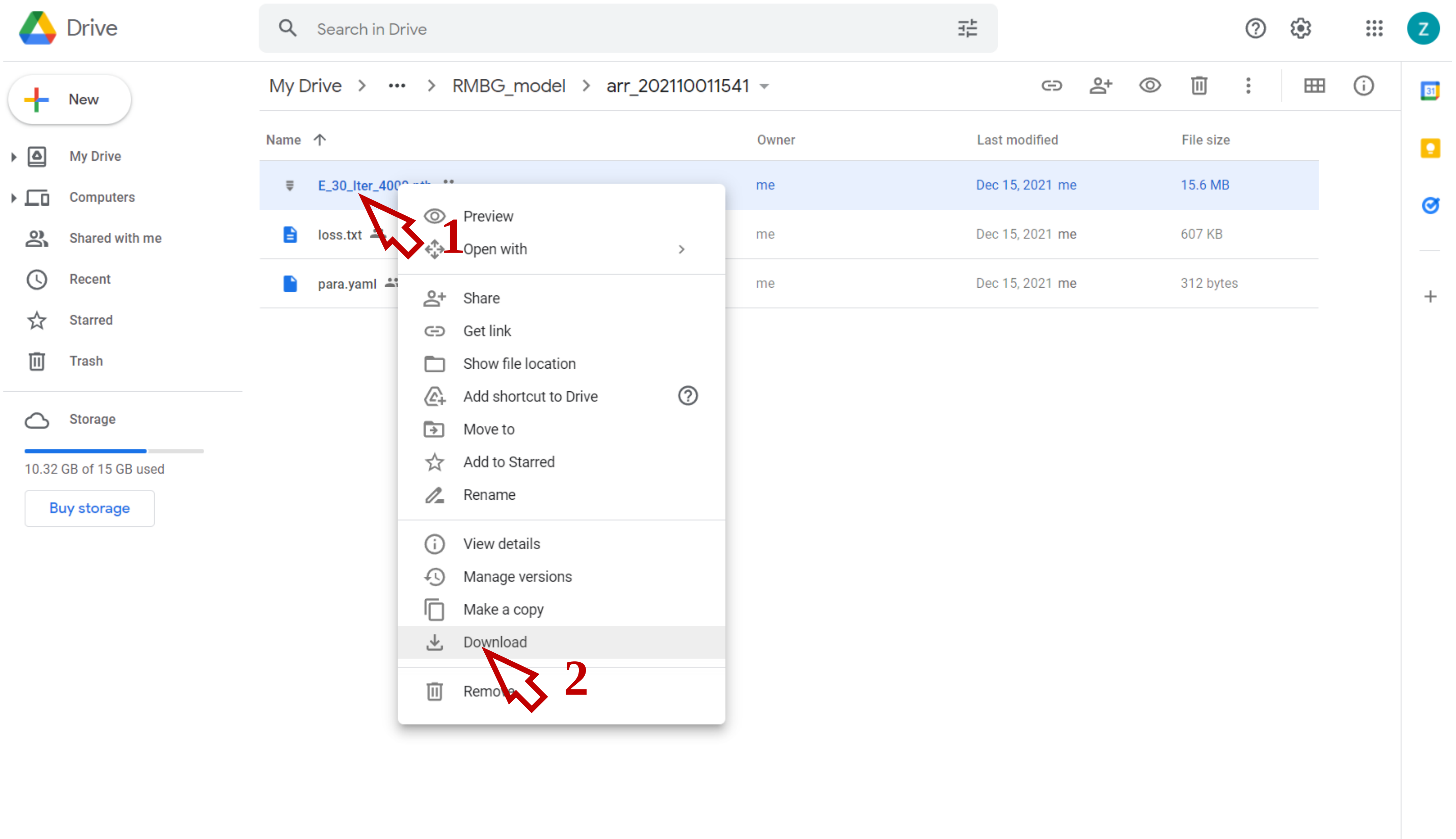

Upload the background removing model

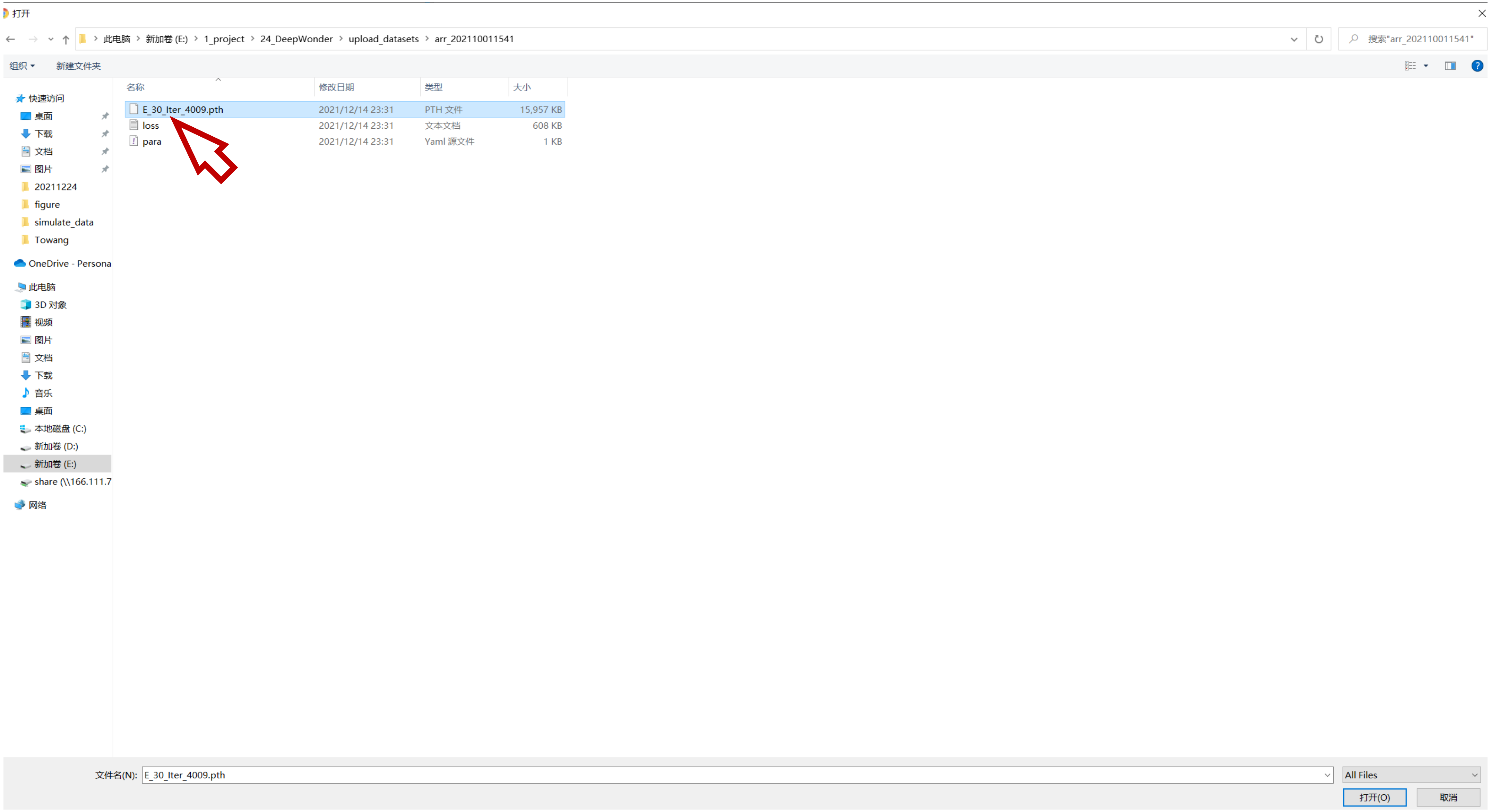

Download the neuron segmentation model

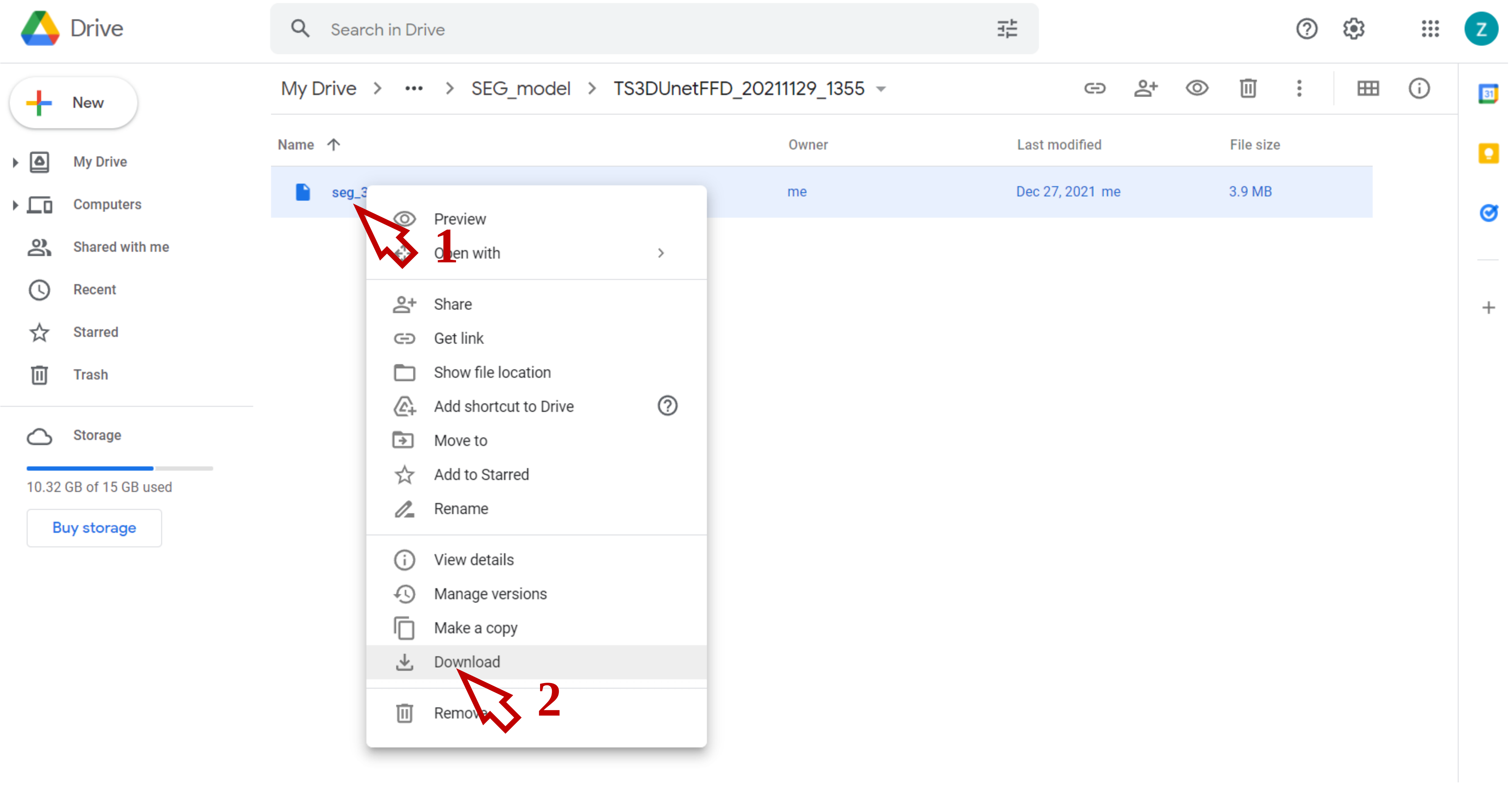

Upload the neuron segmentation model

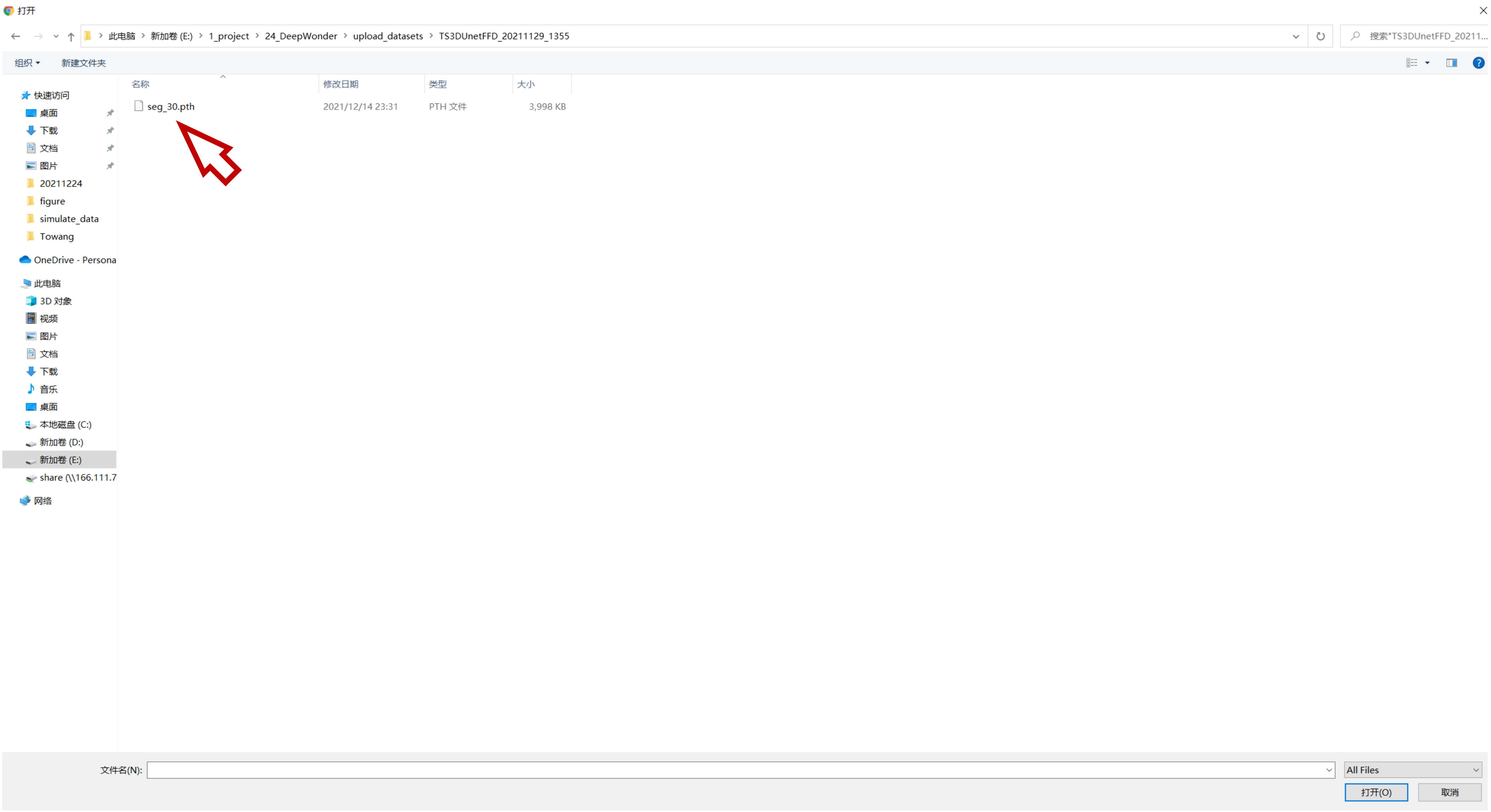

Clear the output from previous stage

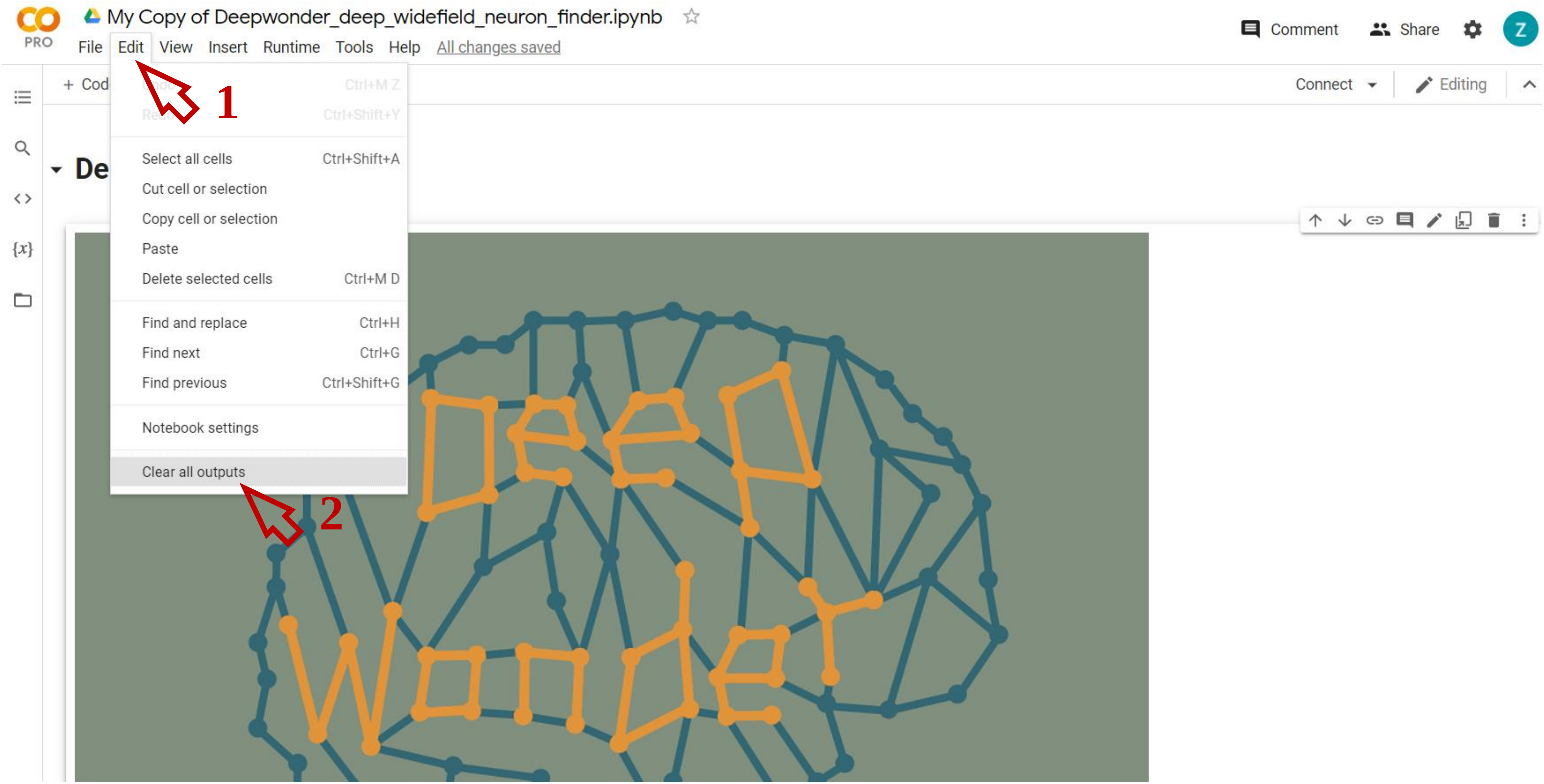

Set GPU

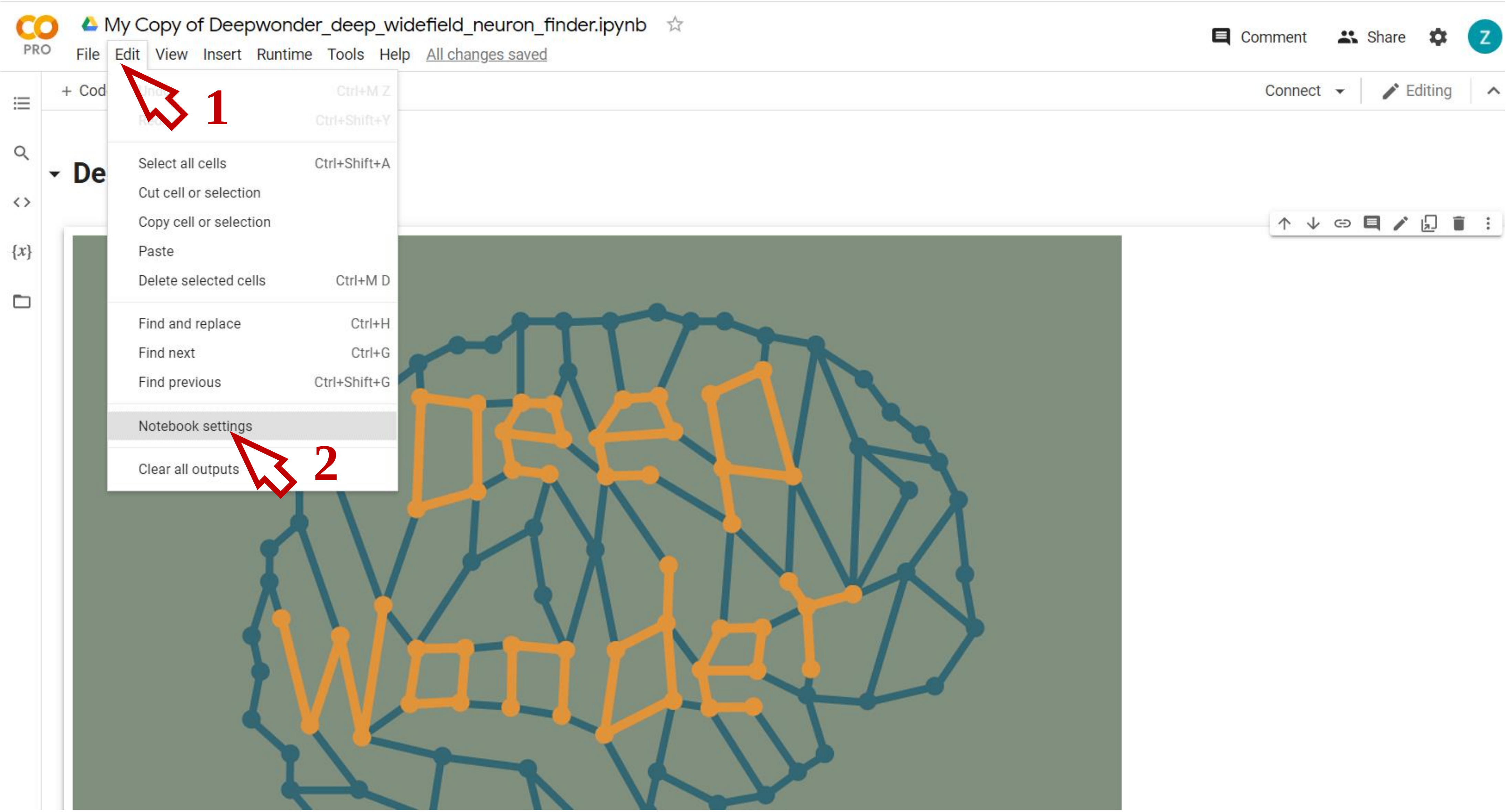

My Copy of Deepwonder_deep_widefield_neuron_finder.ipynb
PRO
File
Edit
View
Insert
Runtime
Tools
Help
All changes saved
Comment
Share
Z
Connect
Editing
1
Select all cells Ctrl+Shift+A
Cut cell or selection
Copy cell or selection
Paste
Delete selected cells Ctrl+M D
Find and replace Ctrl+H
Find next Ctrl+G
Find previous Ctrl+Shift+G
Notebook settings
2
Clear all outputs

Set GPU

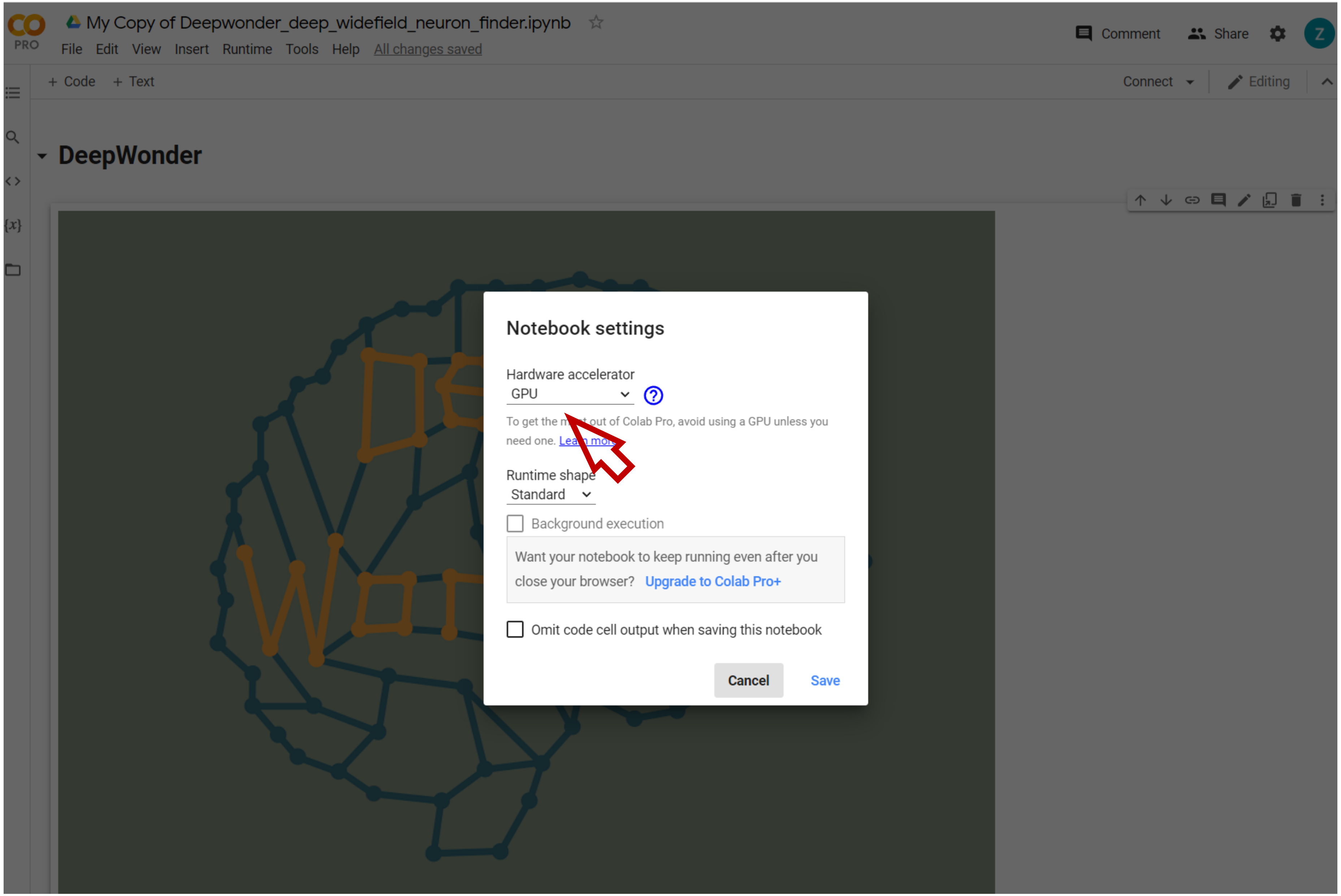

My Copy of Deepwonder_deep_widefield_neuron_finder.ipynb
File Edit View Insert Runtime Tools Help All changes saved
Comment
Share
+ Code + Text
Connect
Editing
DeepWonder
Notebook settings
Hardware accelerator
GPU
Runtime shape
Standard
Background execution
Want your notebook to keep running even after you close your browser? Upgrade to Colab Pro+
Omit code cell output when saving this notebook
Cancel
Save

Install key dependencies

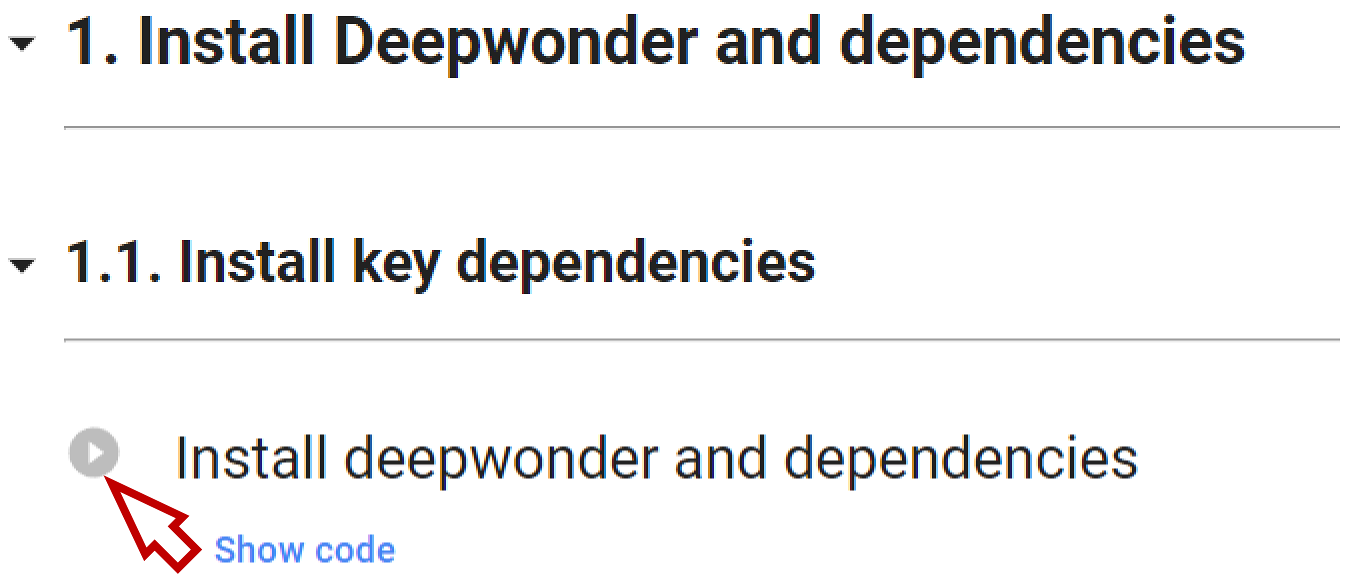

Check whether you have GPU access

2. Check GPU and Google Drive

+ Code + Text

2.1. Check for GPU access

By default, the session should be using Python 3 and GPU acceleration, but it is possible to ensure that these are set properly by doing the following:

Go to **Runtime -> Change the Runtime type**

Runtime type: Python 3 *(Python 3 is programming language in which this program is written)*

Accelerator: GPU *(Graphics processing unit)*

Run this cell to check if you have GPU access

Show code

Check whether you have GPU access

2. Check GPU and Google Drive

2.1. Check for GPU access

By default, the session should be using Python 3 and GPU acceleration, but it is possible to ensure that these are set properly by doing the following:

Go to **Runtime -> Change the Runtime type**

Runtime type: Python 3 *(Python 3 is programming language in which this program is written)*

Accelerator: GPU *(Graphics processing unit)*

Run this cell to check if you have GPU access

Show code

```
You have GPU access
Thu Dec 23 11:36:06 2021
+-----------------------------------------------------------------------------+
| NVIDIA-SMI 495.44       Driver Version: 460.32.03    CUDA Version: 11.2     |
|-------------------------------+----------------------+----------------------+
| GPU  Name        Persistence-M| Bus-Id        Disp.A | Volatile Uncorr. ECC |
| Fan  Temp  Perf  Pwr:Usage/Cap|         Memory-Usage | GPU-Util  Compute M. |
|                               |                      |               MIG M. |
|===============================+======================+======================|
|   0  Tesla P100-PCIE...  Off  | 00000000:00:04.0 Off |                    0 |
| N/A   39C    P0    34W / 250W |    369MiB / 16280MiB |      1%      Default |
|                               |                      |                  N/A |
+-------------------------------+----------------------+----------------------+

+-----------------------------------------------------------------------------+
| Processes:                                                                  |
|  GPU   GI   CI        PID   Type   Process name                  GPU Memory |
|        ID   ID                                                   Usage      |
|=============================================================================|
|  No running processes found                                                 |
+-----------------------------------------------------------------------------+
```

Mount your Google Drive

2.2 Mount your Google Drive

To use this notebook on the data present in your Google Drive, you need to mount your Google Drive to this notebook.

Play the cell below to mount your Google Drive and follow the link. In the new browser window, select your drive and select 'Allow', copy the code, paste into the cell and press enter. This will give Colab access to the data on the drive.

Once this is done, your data are available in the **Files** tab on the top left of notebook.

Play the cell to connect your Google Drive to Colab

- Click on the URL.
- Sign in your Google Account.
- Copy the authorization code.
- Enter the authorization code.
- Click on "Files" site on the right. Refresh the site. Your Google Drive folder should now be available here as "drive".

Show code

Mount your Google Drive

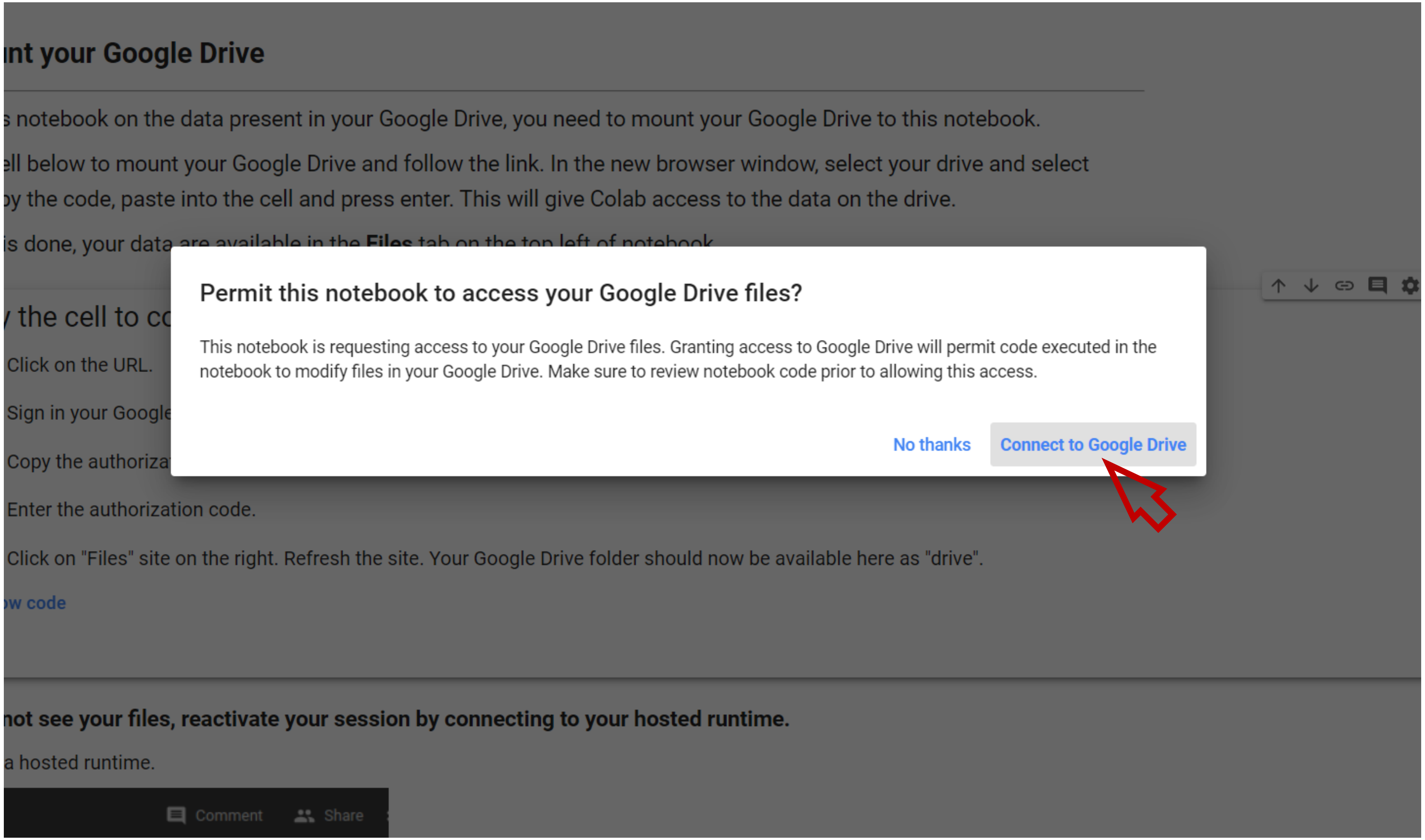

Mount your Google Drive

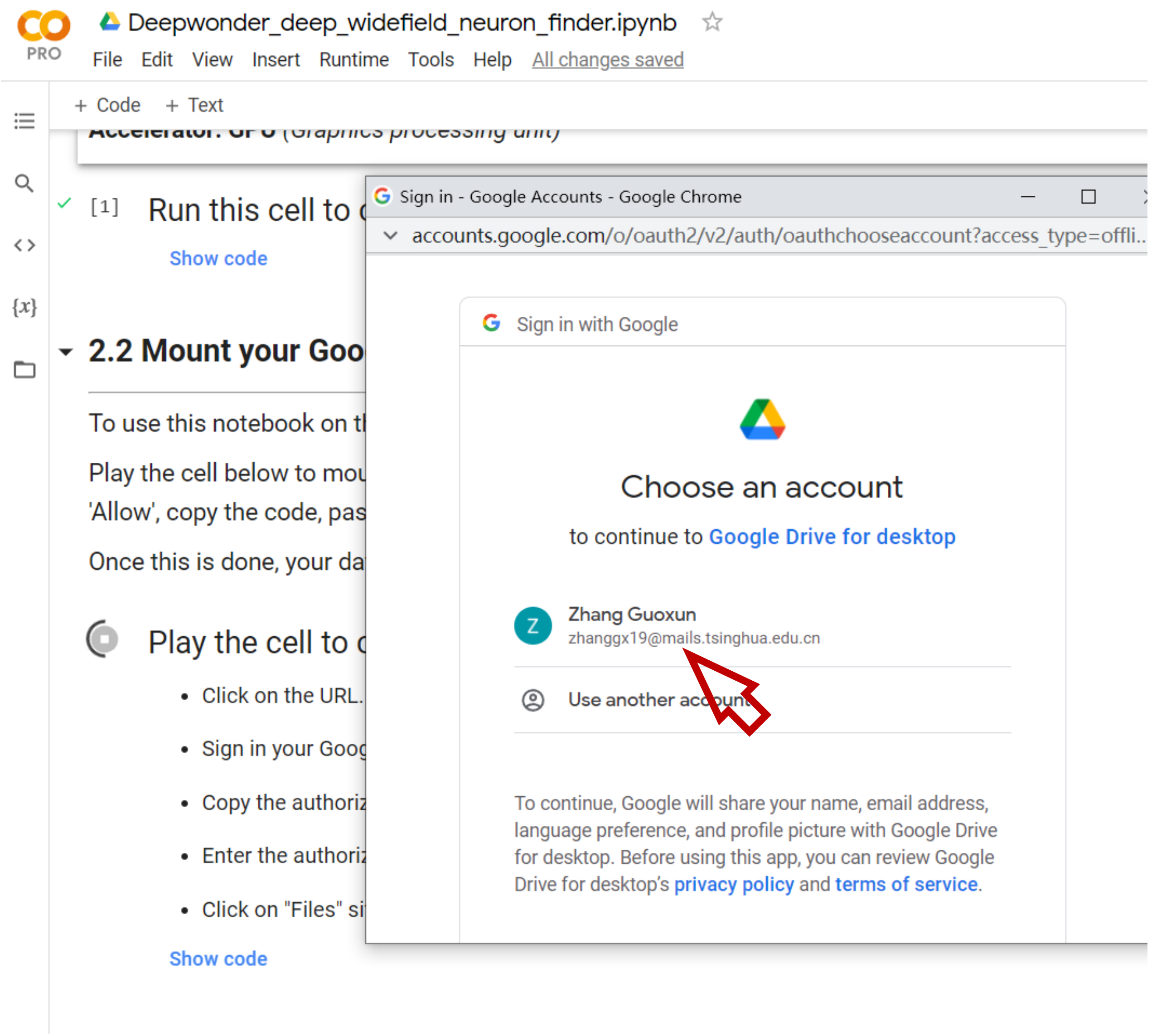

Mount your Google Drive

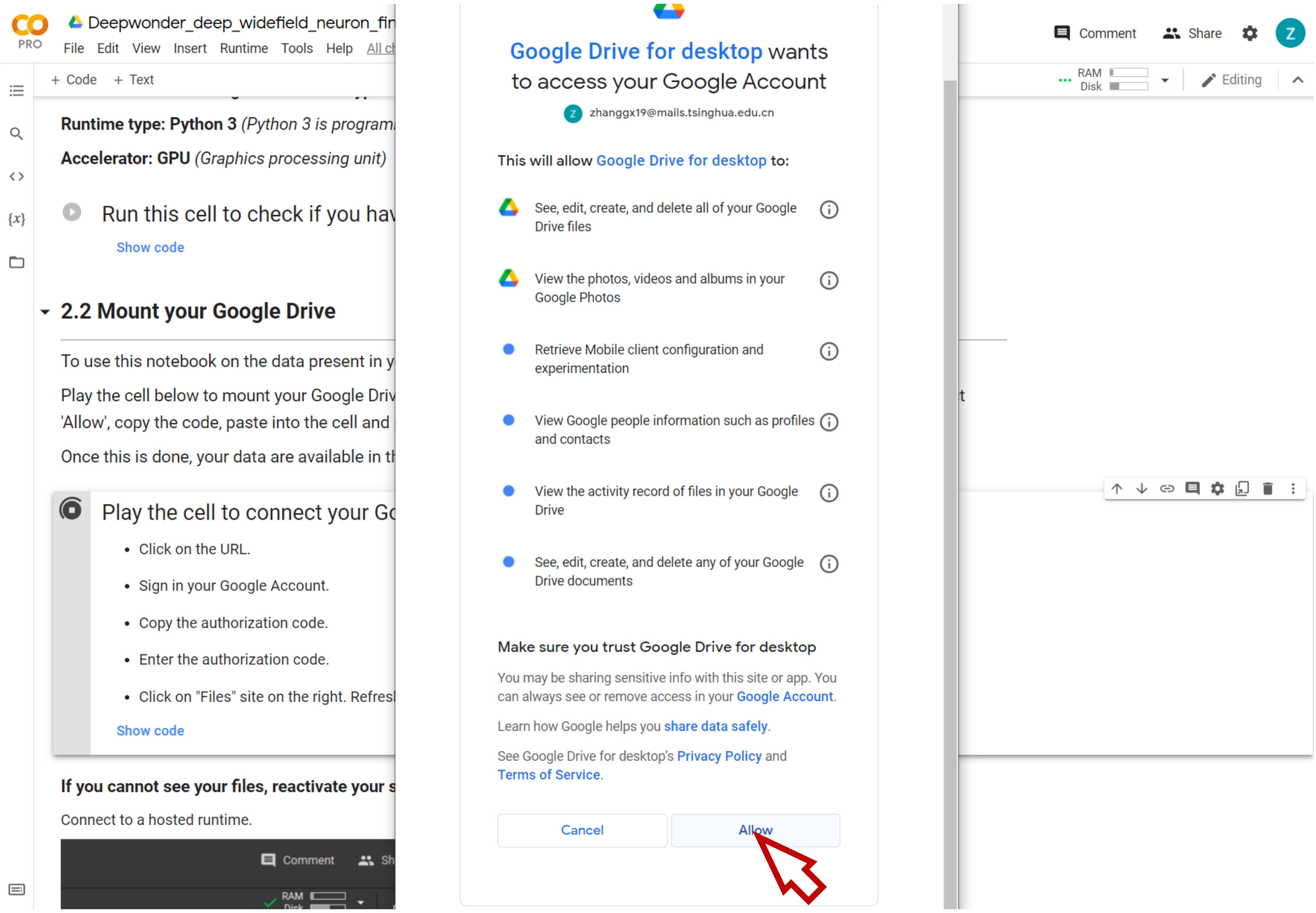

Set the datasets path

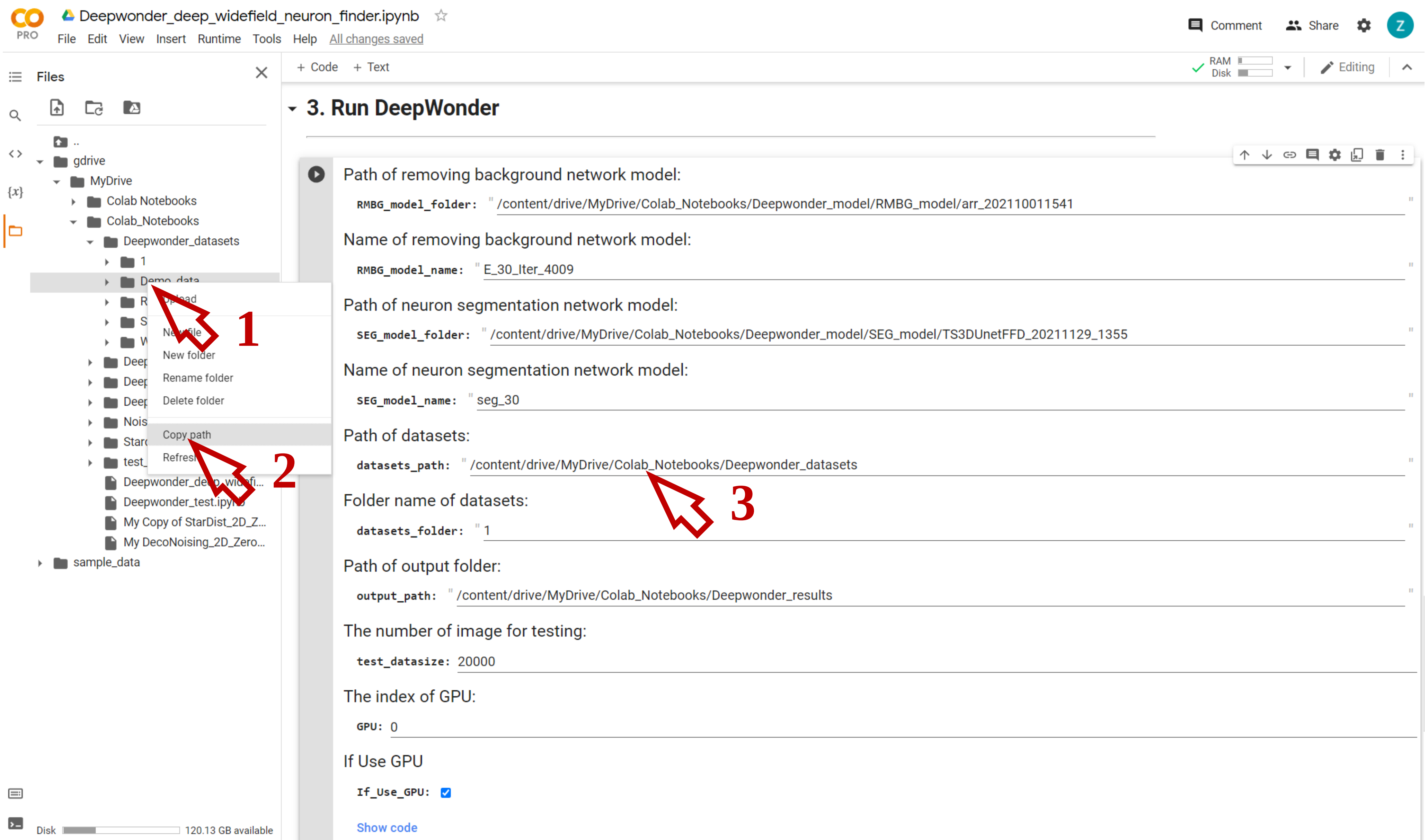

Set the datasets folder

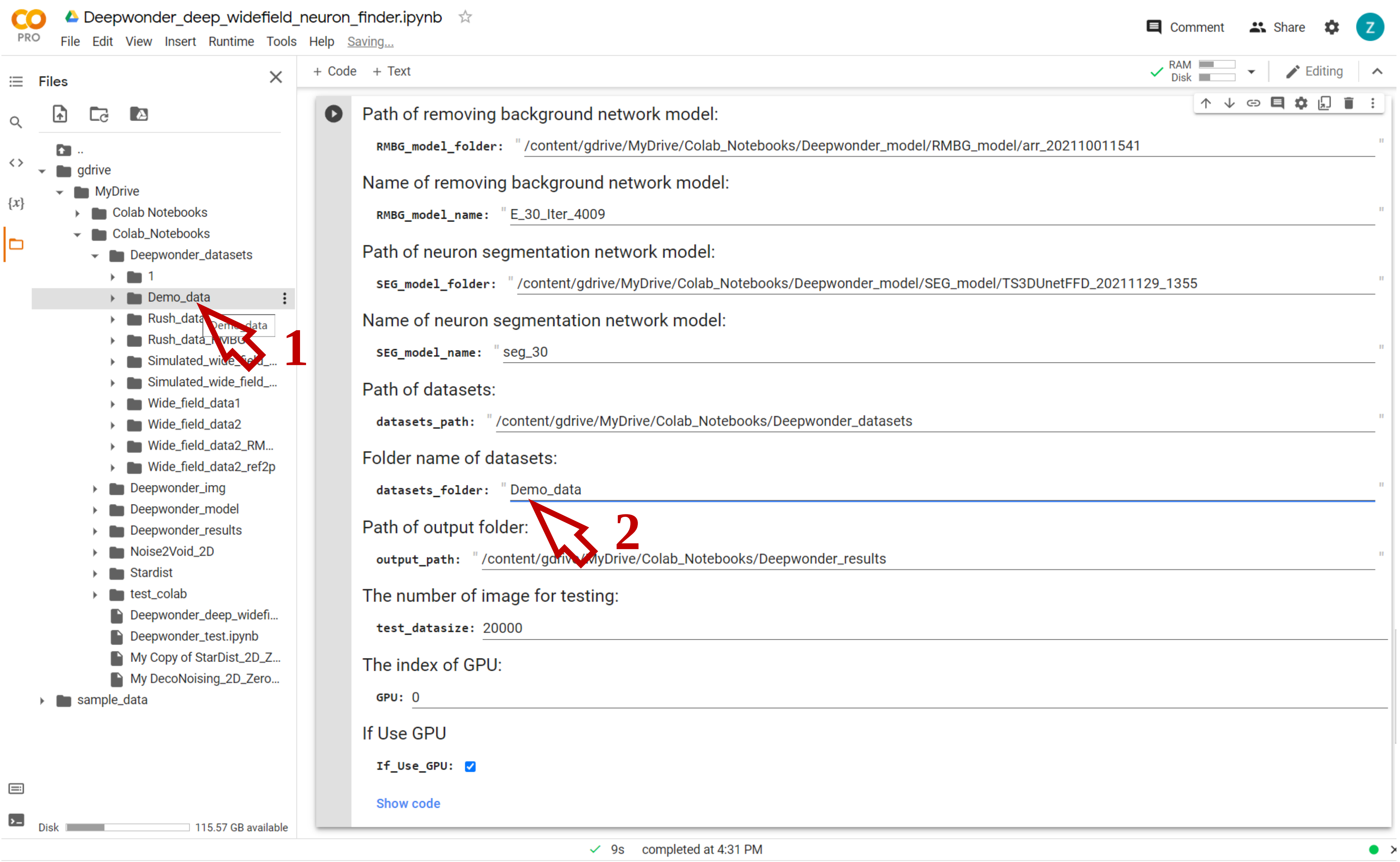

Set the background removing model path

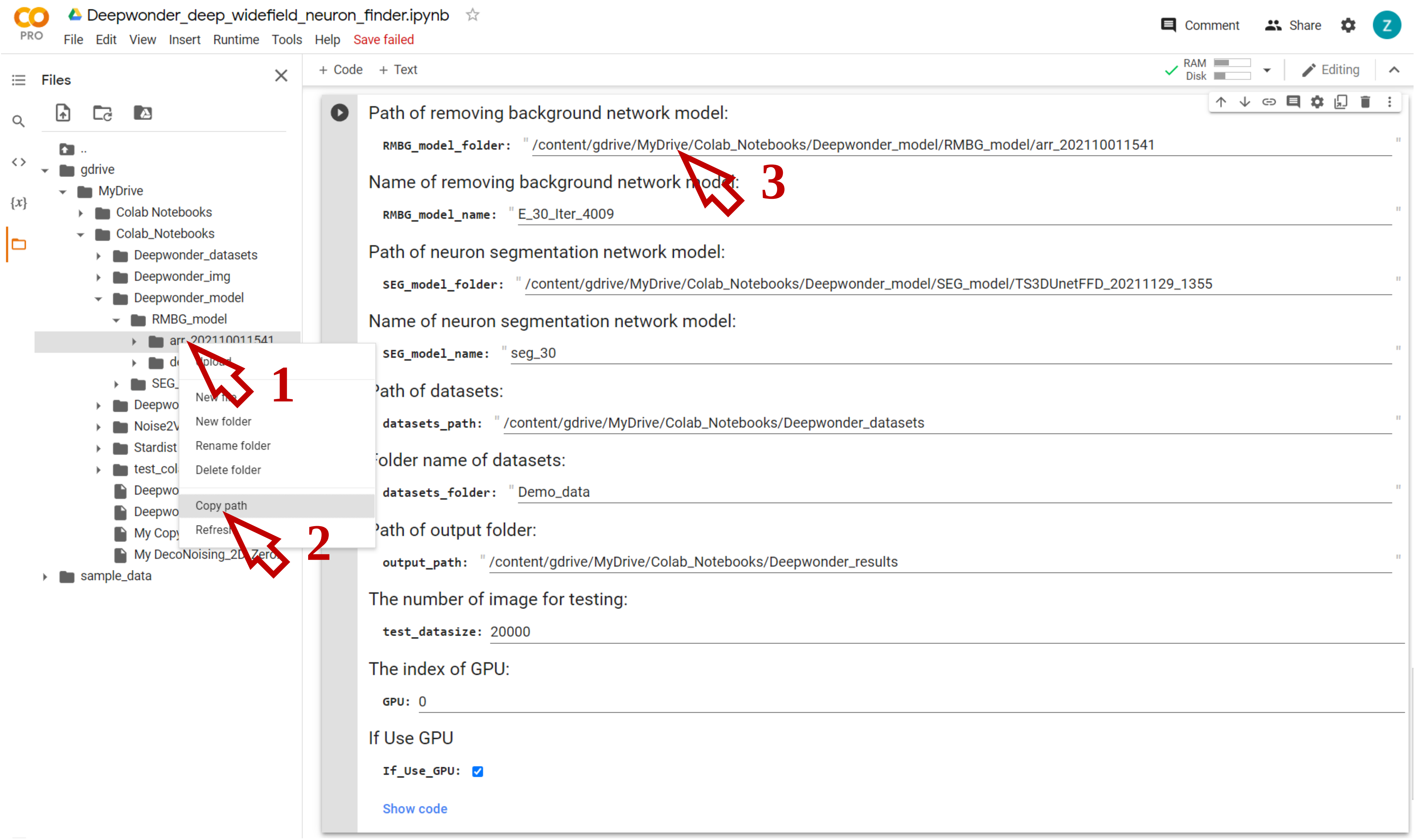

Set the background removing model name

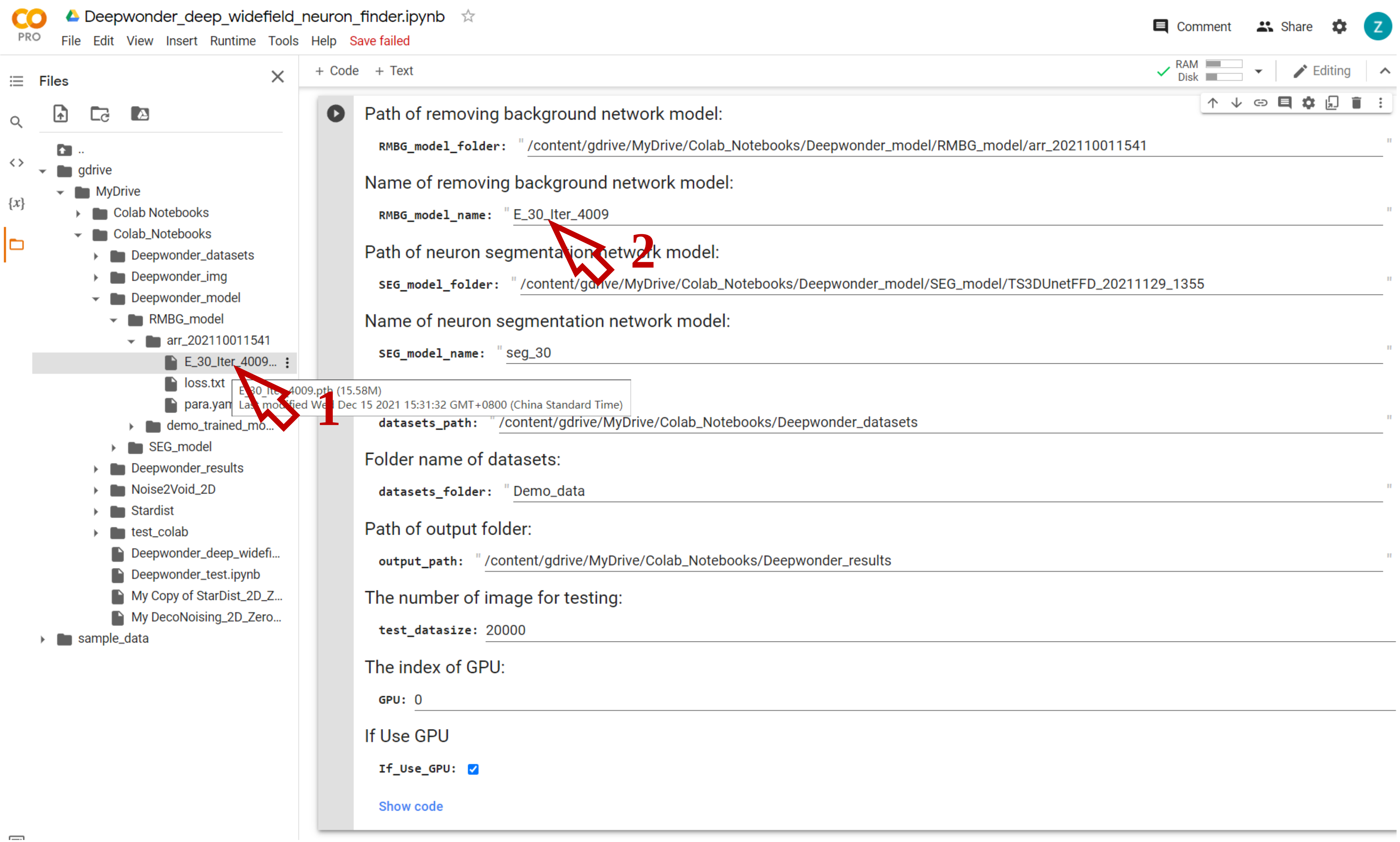

Set the neuron segmentation model path

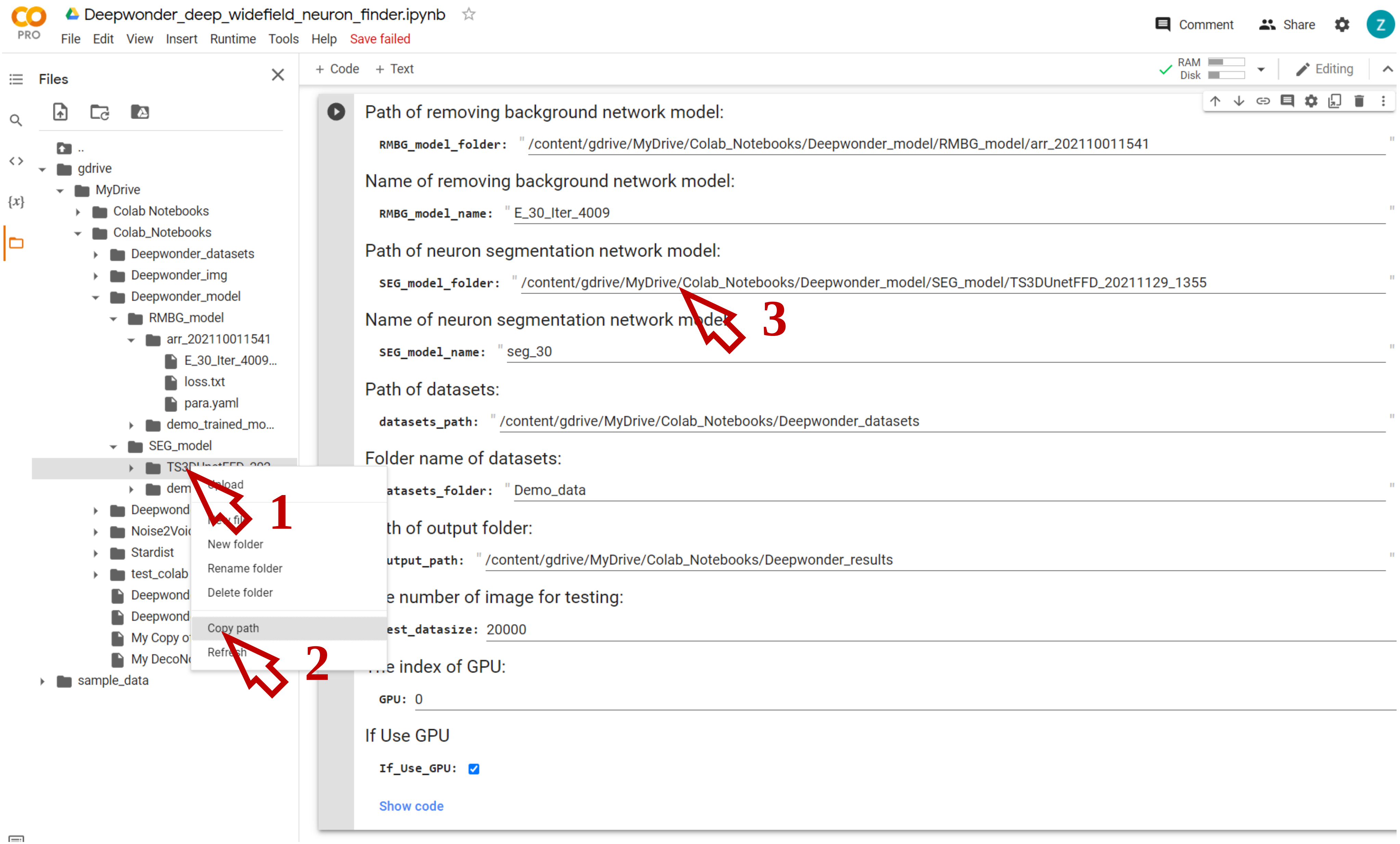

Set the neuron segmentation model name

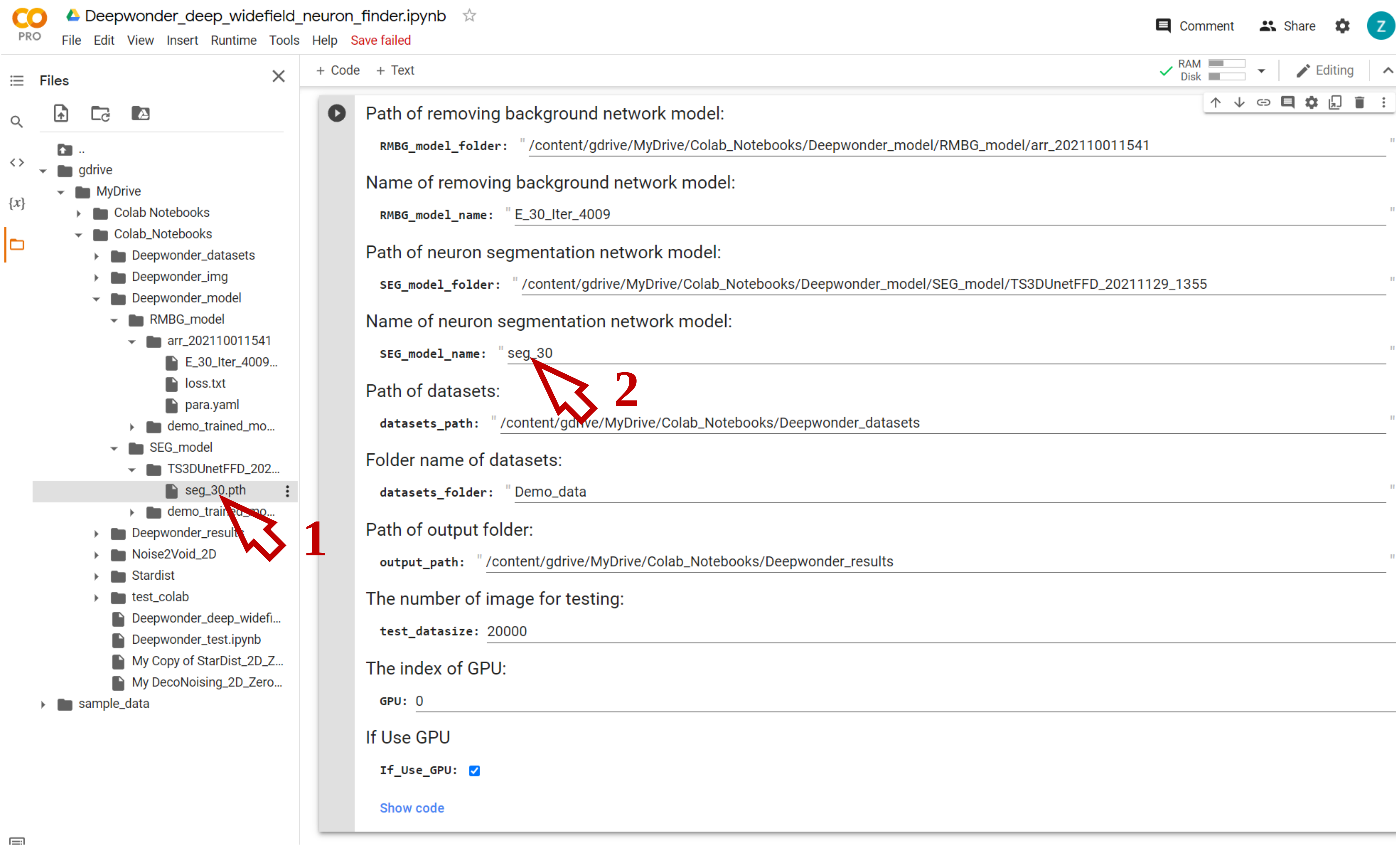

Set the results path

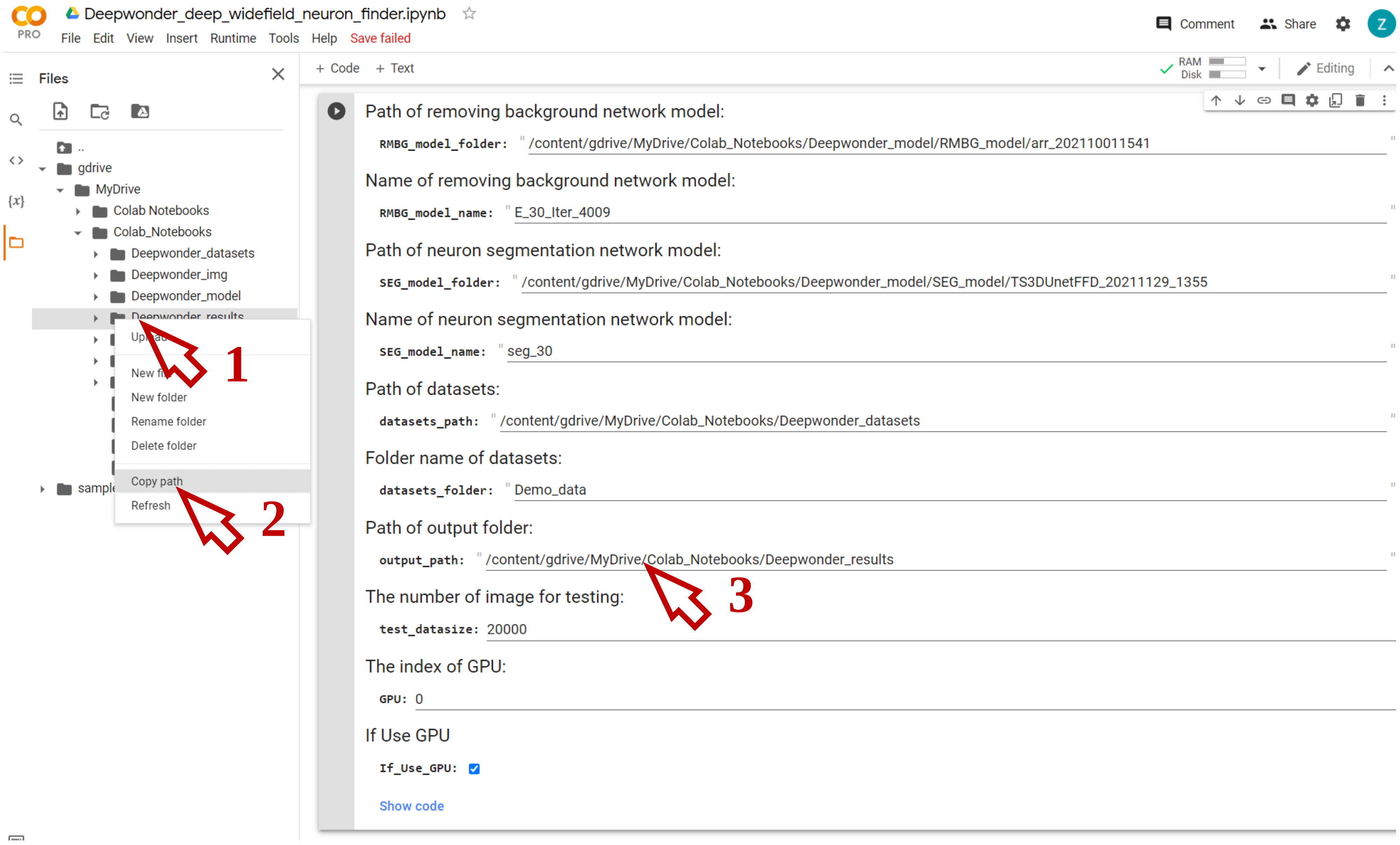

Run the code

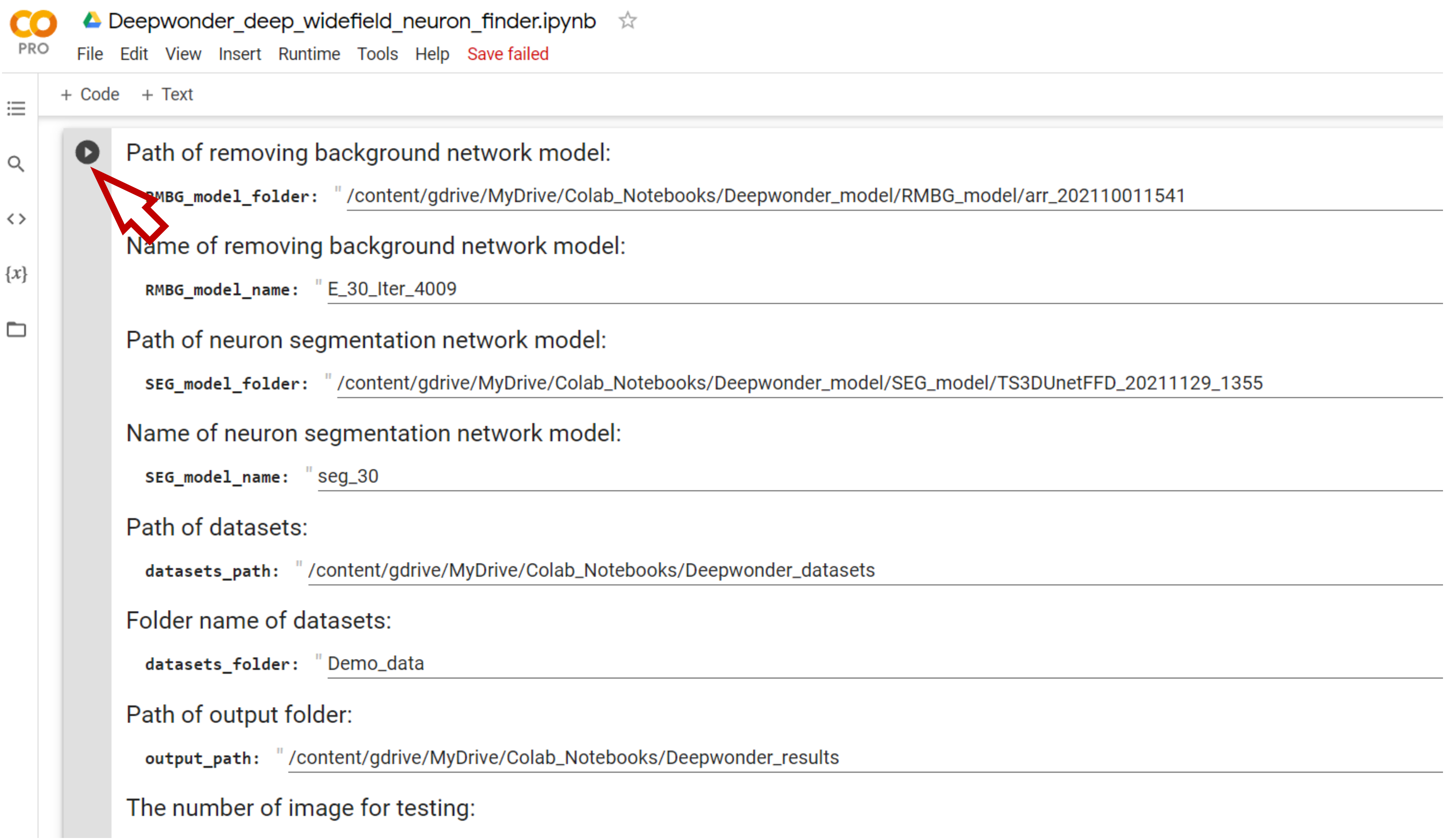
Deepwonder_deep_widefield_neuron_finder.ipynb
PRO
File Edit View Insert Runtime Tools Help Save failed
+ Code + Text
Path of removing background network model:
RMBG_model_folder: /content/gdrive/MyDrive/Colab_Notebooks/Deepwonder_model/RMBG_model/arr_202110011541
Name of removing background network model:
RMBG_model_name: E_30_Iter_4009
Path of neuron segmentation network model:
SEG_model_folder: /content/gdrive/MyDrive/Colab_Notebooks/Deepwonder_model/SEG_model/TS3DUnetFFD_20211129_1355
Name of neuron segmentation network model:
SEG_model_name: seg_30
Path of datasets:
datasets_path: /content/gdrive/MyDrive/Colab_Notebooks/Deepwonder_datasets
Folder name of datasets:
datasets_folder: Demo_data
Path of output folder:
output_path: /content/gdrive/MyDrive/Colab_Notebooks/Deepwonder_results
The number of image for testing:
