# Supplementary figures and images for: Rapid detection of neurons in widefield calcium imaging datasets after training with synthetic data
Source: Nat Methods. 2023 Apr 1;20(5):747–54. doi: 10.1038/s41592-023-01838-7 (PMC10172132; doi:10.1038/s41592-023-01838-7)

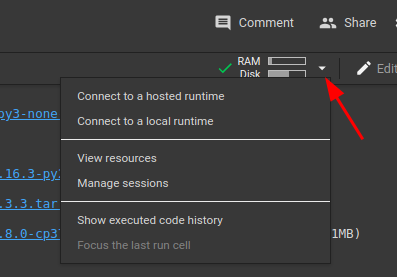

Supplement: Supplementary file 3 — DeepWonder and NAOMi1p computational pipeline, with inline documentation, and demo scripts. See https://github.com/yuanlong-o/Deep_widefield_cal_inferecefor for future updates. [file 41592_2023_1838_MOESM3_ESM.zip › img/connect_to_hosted.png]

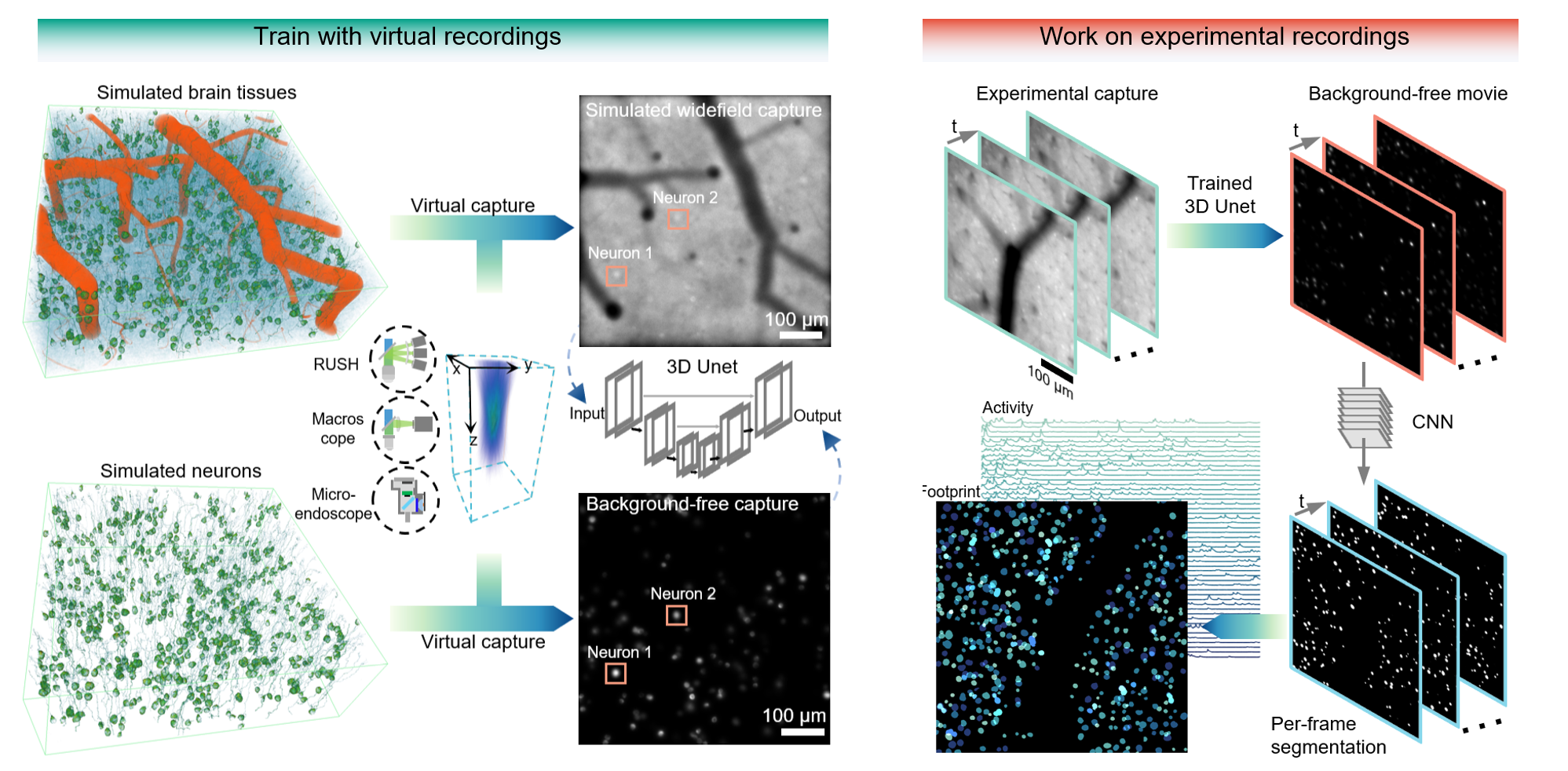

Supplement: Supplementary file 3 — DeepWonder and NAOMi1p computational pipeline, with inline documentation, and demo scripts. See https://github.com/yuanlong-o/Deep_widefield_cal_inferecefor for future updates. [file 41592_2023_1838_MOESM3_ESM.zip › img/Workflow.png]

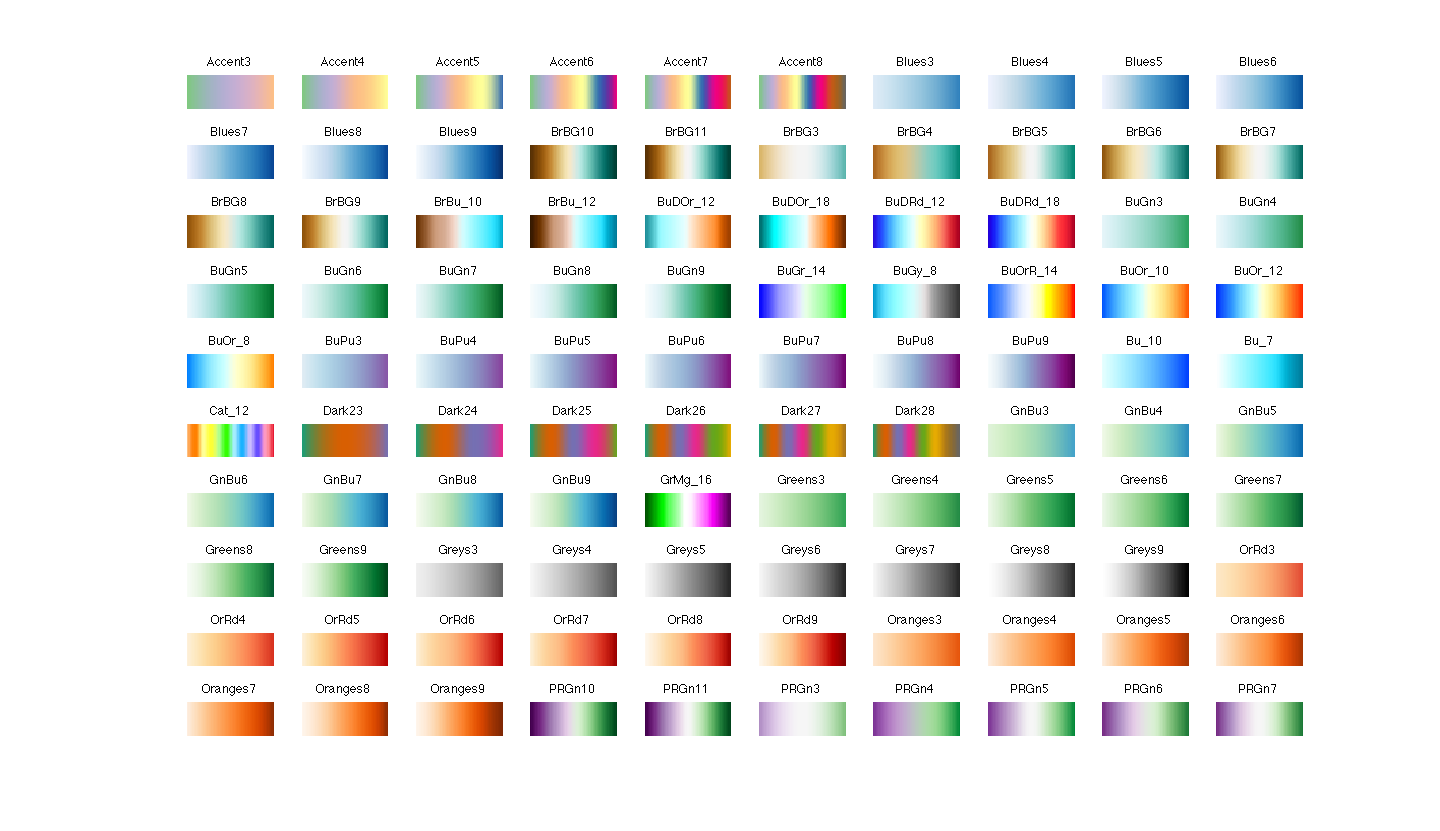

Supplement: Supplementary file 3 — DeepWonder and NAOMi1p computational pipeline, with inline documentation, and demo scripts. See https://github.com/yuanlong-o/Deep_widefield_cal_inferecefor for future updates. [file 41592_2023_1838_MOESM3_ESM.zip › NAOMi1p/ExternalPackages/othercolor/othercolor1-100.png]

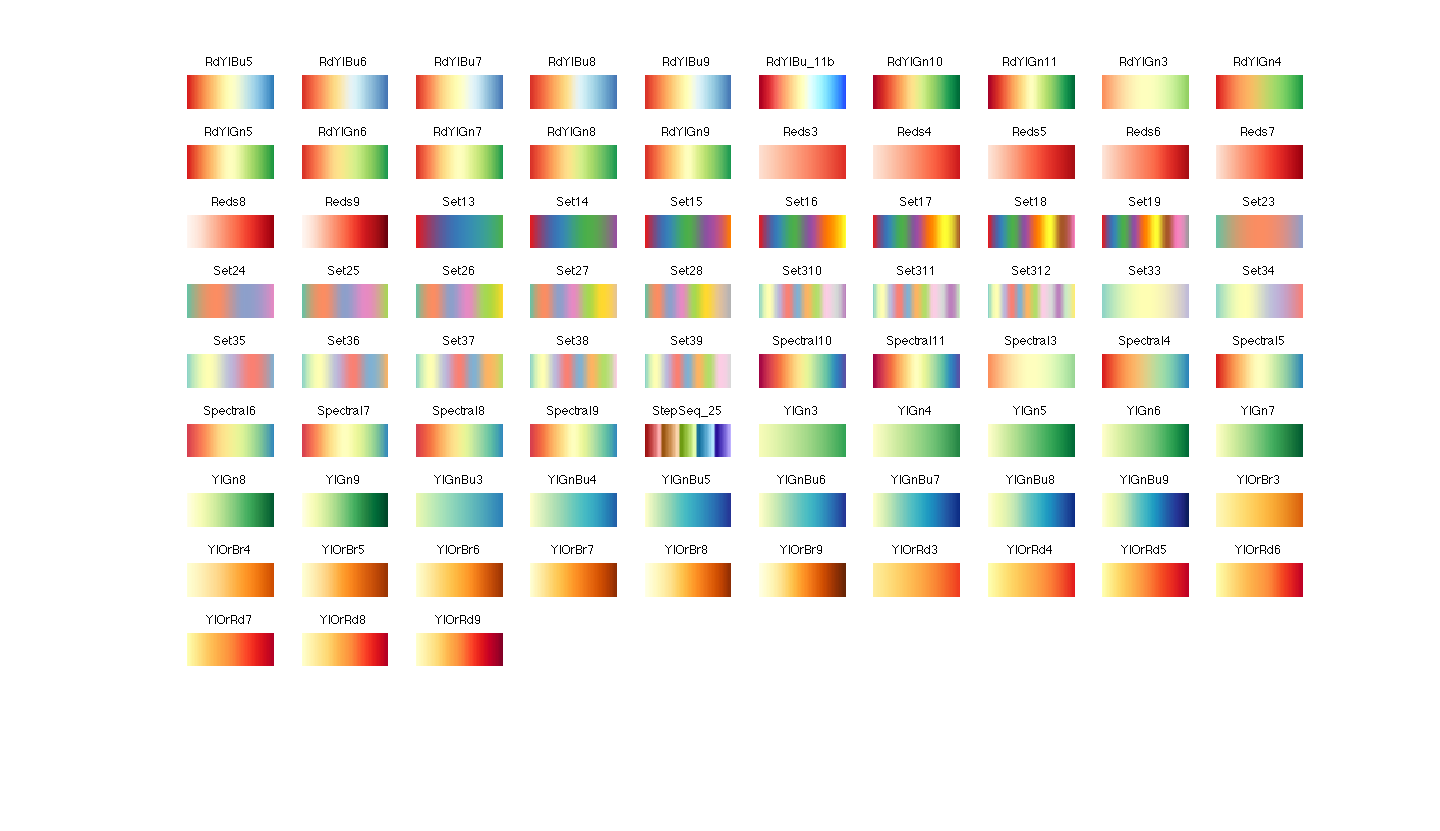

Supplement: Supplementary file 3 — DeepWonder and NAOMi1p computational pipeline, with inline documentation, and demo scripts. See https://github.com/yuanlong-o/Deep_widefield_cal_inferecefor for future updates. [file 41592_2023_1838_MOESM3_ESM.zip › NAOMi1p/ExternalPackages/othercolor/othercolor201-283.png]

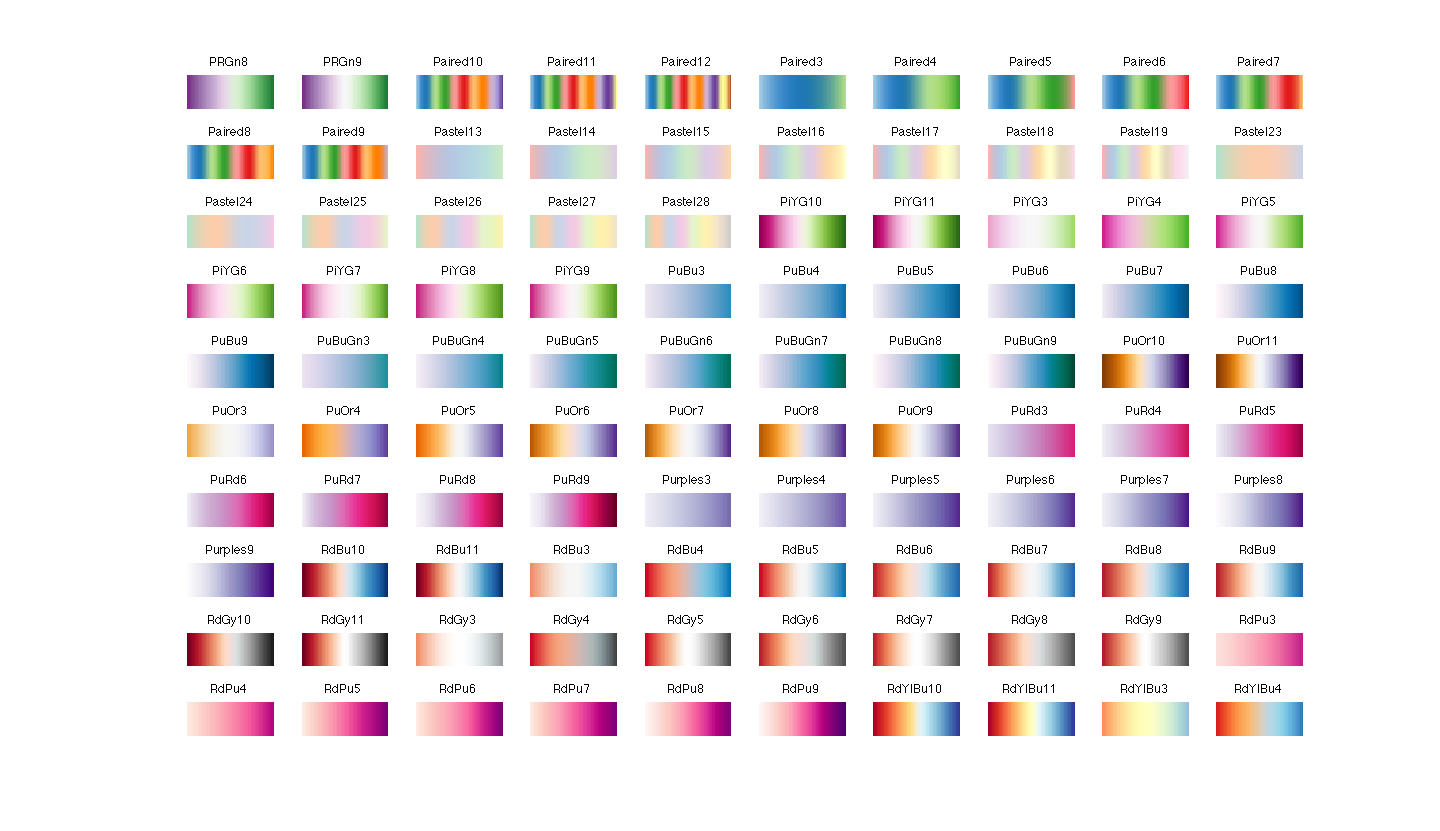

Supplement: Supplementary file 3 — DeepWonder and NAOMi1p computational pipeline, with inline documentation, and demo scripts. See https://github.com/yuanlong-o/Deep_widefield_cal_inferecefor for future updates. [file 41592_2023_1838_MOESM3_ESM.zip › NAOMi1p/ExternalPackages/othercolor/othercolor_101-200.png]
